# Tumor Immunometabolism Characterization in Ovarian Cancer With Prognostic and Therapeutic Implications

**DOI:** 10.3389/fonc.2021.622752

**Published:** 2021-03-16

**Authors:** Miner Yang, Gaowen Chen, Kunjie Gao, Yifeng Wang

**Affiliations:** Department of Gynecology, Obstetrics and Gynecology Center, Zhujiang Hospital, Southern Medical University, Guangzhou, China

**Keywords:** ovarian cancer, metabolism reprogramming, epinephrine biosynthesis, major histocompatibility complex, immune microenvironment

## Abstract

Metabolic dysregulation in the tumor microenvironment has significant impact on immune infiltration and immune responses. However, interaction between immunity and metabolism in the ovarian microenvironment requires further exploration. We constructed an immunometabolism gene set and ovarian cancer cohort from The Cancer Genome Atlas (TCGA) and classified these into three immunometabolism subtypes. We explored the relationships between immune infiltration and metabolic reprogramming. Additionally, we built risk score and nomogram as prognostic signatures. Three distinctive immunometabolism subtypes were identified with therapeutic and prognostic implications. Subtype 1, the “immune suppressive-glycan metabolism subtype,” featured high levels of immunosuppressive cell infiltration and glycan metabolism activation; Subtype 2, the “immune inflamed-amino acid metabolism subtype,” showed abundant adaptive immune cell infiltration and amino acid metabolism activation; Subtype 3, the “immune desert-endocrine subtype,” was characterized by low immune cell infiltration and upregulation of hormone biosynthesis. Furthermore, we found that epinephrine biosynthesis displayed a significantly negative correlation with MHC molecules, which may result in defective antigen presentation. We proposed immunometabolism subtypes with prognostic implications and provided new perspectives for the ovarian cancer microenvironment.

## Introduction

Ovarian cancer is the common cause of death related to gynecological cancer (1, 2). The standard treatment of ovarian cancer is surgical resection with cisplatin-based chemotherapy (3). However, about 70% of patients will experience a recurrence within 3 years after first-line treatment (4).

In the past decade, chemotherapy has maintained its pivotal role in drug therapy for ovarian cancer. Very few new drug strategies have been approved. Recently, poly ADP ribose polymerase (PARP) inhibitors have been approved for maintenance therapy (5). More therapeutic perspectives should be proposed to optimize ovarian cancer treatment. Immunotherapy is one of the most effective new treatment strategies, including immune checkpoint blockades, cancer vaccines, and cell-based therapy (6). It has showed efficiency in melanoma (7) and non-small-cell lung cancer (NSCLC) (8). Immunotherapy in ovarian cancer is undergoing clinical trials, but so far, the results of these clinical trials are not satisfying, and the underlying mechanisms and effective immunotherapy subgroup have not been elucidated (9). Infiltration of immune cells in the ovarian cancer microenvironment is one of the key factors for immunotherapy response. Further exploration of the immune microenvironment and screening of biomarkers is needed.

Accumulating research has indicated that microenvironment cells, including immune cells and stromal cells, are important components of the tumor microenvironment (10). They are associated with prognosis and immunotherapy response in multiple malignant tumors, such as melanoma, gastric cancer, lung cancer, and breast cancer. For example, CD8^+^ cytotoxic T cells can effectively kill tumor cells (11); dendritic cells can capture tumor antigen and urge effective immune response of T cells (12); cancer associated fibroblasts (CAFs), as important components of tumor stroma, indirectly regulate migration and invasion of tumor cells by remodeling tumor matrix (13). Different patterns of immune infiltration, including immune inflamed, immune suppressive, and immune desert have been widely known and have important effects on anti-tumor immune responses (14).

The field of immunometabolism has emerged in recent years and focuses on the interaction between the immune microenvironment and metabolism processes. Evidence shows that dysregulated metabolism of cancer cells and metabolite accumulation may suppress immune cell activation, causing impaired anti-tumor immune responses (15). For example, lactic acid produced by tumor cells *via* glycolysis regulated expression of granulocyte colony-stimulating factor (G-CSF) and granulocyte-macrophage colony-stimulating factor (GM-CSF), promoting myeloid-derived suppressor cells (MDSCs) and inhibiting the maturation of dendritic cells (16). Drugs targeting tumor metabolism can synergistically enhance immunotherapy *via* metabolic reprogramming (17). Thus, targeted strategies based on the interaction of metabolism and immunity might facilitate immunotherapy.

To investigate immune infiltration and metabolic reprogramming in ovarian cancer, we defined an immunometabolism gene set, clustered The Cancer Genome Atlas (TCGA) ovarian cohort into three immunometabolism subtypes, and explored interactions between immune infiltration and metabolic reprogramming. This research provides insights into individual therapy for ovarian cancer and new perspectives for identifying potential groups that would benefit from immunotherapy.

## Materials and Methods

### Patients and Samples

Gene expression profiles of human ovarian cancer were obtained from The Cancer Genome Atlas Project (TCGA) (https://portal.gdc.cancer.gov/) and GEO datasets (https://www.ncbi.nlm.nih.gov/geo/). Transcriptome raw count data of TCGA-OV cohort were downloaded from the GDC data portal with 379 samples including 374 primary and five recurrent tumor samples. Full clinical characteristics of ovarian cancer patients were downloaded from cBioPortal (https://www.cbioportal.org/). For TCGA data set, RNA-sequencing data (count values) were transformed into transcripts per kilobase million (TPM) values. The GSE9891, GSE18520, GSE19829, GSE26193, GSE30161, GSE63885, and GSE115635 from GPL570, the GSE73614 from GPL6480 and the GSE140082 from GPL14951 were downloaded from the GEO database (**Supplementary Table S1**). Based on the annotation of GPL6480, GPL14951, and GPL570, probe mapping was applied to genes. If various probes matched to one gene, we took the median and deleted probes matched to multiple genes. The validation cohort 1 (GSE9891, GSE18520, GSE19829, GSE26193, GSE30161, and GSE63885 from GPL570) and the validation cohort 2 (GSE73614 from GPL6480) were external cohorts. The validation cohort 1 was preprocessed with batch effect removal using sva package (18) and standardization algorithm. Immunohistochemistry (IHC) staining images of ovarian cancer were extracted from the Human Protein Atlas (19) (http://www.proteinatlas.org).

### Identification of Immunometabolism Gene Set and Subtype Analysis

We downloaded 1784 metabolic genes from the Kyoto Encyclopedia of Genes and Genomes (KEGG) database and 1811 immune-related genes from the Immport database, which includes 17 immune categories in terms of different functions. The univariate Cox proportional hazards model was used to assess their association with overall survival. Eventually, genes meet the requirements of HR > 1.2 or HR < 0.8 and *P* values < 0.05 were used for sample clustering. Consequently, unsupervised consensus clustering was performed using R package ConsensusClusterPlus (20), and this method was applied to validation cohort 1, validation cohort 2, and GSE140082 with the same gene set. The values of k where the magnitude of the cophenetic correlation coefficient began to fall were chosen as the optimal number of clusters. Hierarchical clustering was performed by the hclust function. Boruta, a novel random forest algorithm-based feature selection method, was used to select characteristic genes of the immunometabolism gene set (21).

### Differentially Expressed Gene Analysis and Functional Enrichment Analysis

The differentially expressed genes (DEGs) among ovarian cancer subtypes were validated using the DEseq2 package in R (22). The genes with an absolute Log2 (fold change) > 1 and *P* < 0.05 were defined as DEGs. The gene set “hallmark gene set,” “KEGG gene set,” “GO biological processes,” “GO cellular components,” and “GO molecular functions,” downloaded from the Molecular Signatures Database (MsigDB, https://www.gsea-msigdb.org/gsea/msigdb), were used for functional enrichment analysis using clusterprofiler package (23). Eventually, significantly enriched pathways (*P* < 0.05) were ordered based on consensus scores. Top 10 pathways with the highest consensus scores were selected for each subtype and used for heatmap visualization.

### Immune Cell Infiltration and Metabolic Pathway Scores

Single sample gene set enrichment analysis (ssGSEA) with GSVA R package (24) was employed to evaluate the enrichment scores of 23 immune cells and two stromal cells for each sample based on the expression profiles. Genes representing 23 immune cell and two stromal cell signatures were downloaded from Yi Xiao’s research (25). Subsequently, differential analysis of the enrichment scores was performed using the limma package (26). Metabolic pathway gene sets were downloaded from KEGG and classified into major categories. ssGSEA was used to calculate the enrichment scores of metabolic pathways. Differential analysis of enrichment scores of each metabolic pathway was performed using the limma package.

### Development of the Prognostic Gene Expression Signature

The genes in the immunometabolism gene set were selected as prognostic genes using Lasso-Cox penalized algorithm (27). Risk scores were calculated based on the expression values of genes and Lasso-penalized regression coefficients and its prognostic value was tested using ROC curves with survivalROC package (28).

### Bioinformatic Methods and Statistical Analysis

Function enrichment analysis was performed using GSEA Java (ver 1.3.0). According to the Genomics of Drug Sensitivity in Cancer (GDSC, https://www.cancerrxgene.org/) database, the IC_50_ for each subtype was estimated using the pRRophetic R package (29). For comparisons of the three groups, Kruskal-Wallis tests and one-way analysis of variance (ANOVA) were employed as nonparametric and parametric methods, respectively. Correlation coefficients were calculated using Spearman analysis. Chi-square and Fisher’s test were used for contingency table variables. Overall survival time was used as primary clinical endpoint and progression free survival was the secondary. Overall survival was chosen as it is the gold standard for measuring the clinical benefits of drugs in clinical trials. Survival analysis was performed using Kaplan-Meier. PROC package (30) was employed to plot receiver operating characteristic curves and calculated the area under the curve. Tumor Immune Dysfunction and Exclusion (TIDE, http://tide.dfci.harvard.edu/) and submap (https://cloud.genepattern.org/gp/pages/index.jsf) were used for immune checkpoint blocks (29). All statistical analyses were performed using R software v3.5.0. A *P* value < 0.05 was considered statistically significant.

## Results

### Identification of Immunometabolism Subtypes With Prognosis Value in Ovarian Cancer

Lymphocytes, such as CD8^+^, CD4^+^ T cells, and natural killer (NK) cells, infiltrating the tumor is a prerequisite for a successful anti-tumor immune response (31). The emerging field of immunometabolism provides new perspectives for regulating immune processes. Immune responses are influenced by tumor metabolism, such as nutrient consumption, increased oxygen consumption, and the production of reactive nitrogen and oxygen intermediates (32).

**Figure 1:**
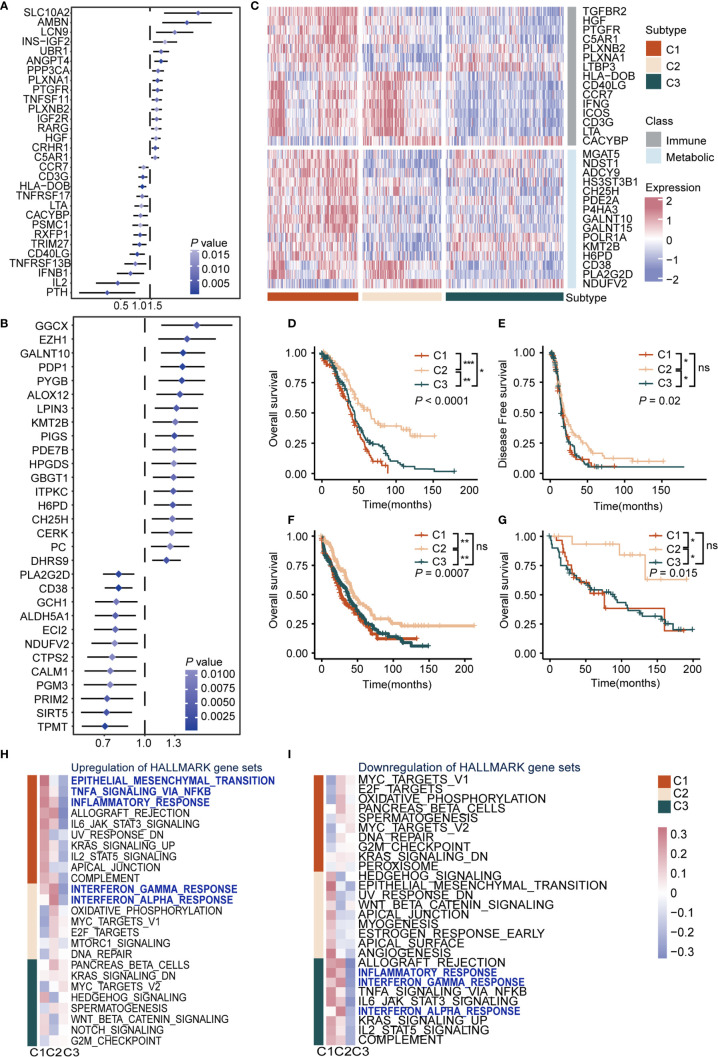
Definition of the immunometabolism gene set and identification of immunometabolism subtypes in ovarian cancer **(A)** Hazard ratio of top 30 immune genes meet the requirements of HR < 0.8 or HR > 1.2 and *P* < 0.05 associated with overall survival. **(B)** Hazard ratio of top 30 metabolic genes meet the requirements of HR < 0.8 or HR > 1.2 and *P* < 0.05 associated with overall survival. **(C)** Unsupervised clustering based on the 170 selected immunometabolism genes, with 30 genes defined as characteristic genes for 374 patients in TCGA cohort. **(D, E)** Kaplan-Meier curves of overall survival **(D)**, Log-rank test: *P* < 0.0001, and disease-free survival **(E)**, Log-rank test: *P* = 0.02 among the subtypes in the TCGA cohort. **(F, G)** Kaplan-Meier curves of overall survival among the subtypes in validation cohort 1 **(F)**, Log-rank test: *P* = 0.0007, and validation cohort 2 **(G)**, Log-rank test: *P* = 0.015. **(H, I)** Hallmark pathways from GSEA database were applied in enrichment analysis of DEGs in the three subtypes. Significant upregulated **(H)** or downregulated **(I)** pathways were demonstrated. Heatmap showed mean pathway scores constructed using the GSVA algorithm. (*P < 0.05; **P < 0.01; ***P < 0.001; ^ns^ *P* > 0.05).

To understand the impact of immune cells as well as metabolic reprogramming, univariate Cox proportional hazards regression model analysis was used to evaluate the prognostic value of 1784 metabolic genes in 113 KEGG pathways and 1811 immune genes from the Immport database. Results showed immune-related genes *SCLC10A2* (HR = 5.58, *P* = 0.005, 95% CI, 1.65–18.8), *AMBN* (HR = 3.74, *P* = 0.003, 95% CI, 1.15–9.01), and *LCN9* (HR = 2.42, *P* = 0.009, 95% CI, 1.25–4.70) were the top three significant factors implicating unfavorable prognosis, and *PTH* (HR = 0.22, *P* = 0.003, 95% CI, 0.08–0.59), *IL2* (HR = 0.32, *P* = 0.004, 95% CI, 0.15–0.69), and *IFNB1* (HR = 0.50, *P* = 0.005, 95% CI, 0.31–0.81) were significant protective factors (**Figure 1A** and **Supplementary Table S2**). Previous reports showed that *SCLC10A2* (33) is associated with tumor proliferation in breast cancer, while *IL2* plays a major role in promoting the cytotoxic activity of CD8^+^ T cells against tumor cells (34).

As for metabolic genes, *GGCX* (HR = 1.57, *P* = 0.004, 95% CI, 1.16–2.16), *EZH1* (HR = 1.45, *P* = 0.004, 95% CI, 1.13–1.87), and *GALNT10* (HR = 1.40, *P* = 0.007, 95% CI, 1.15–1.70) were the top three risk factors. Meanwhile, *TPMT* (HR = 0.70, *P* = 0.007, 95% CI, 0.57–0.86), *SIRT5* (HR = 0.71, *P* = 0.003, 95% CI, 0.57–0.89), and *PRIM2* (HR = 0.72, *P* = 0.005, 95% CI, 0.57–0.90) were the top three protective factors (**Figure 1B** and **Supplementary Table S2**). It is reported that *EZH1* promotes renal cell carcinoma proliferation and is used as a prognostic indicator and therapeutic target (35). In addition, high levels of *SIRT5* are reportedly associated with improved outcomes for ovarian cancer patients (36), which was in accordance with our results. Furthermore, we screened genes from KEGG and the Immport database and retained genes that met the requirements of HR > 1.2 or HR < 0.8 and *P* < 0.05 in the Cox regression model to construct an immune and metabolic gene set (a 170-gene set named the immunometabolism gene set, including 97 metabolic genes and 73 immune genes) (**Supplementary Table S3**). The feature selection method Boruta was used to determine 30 genes with the highest importance. In addition, we compared the importance of immune and metabolic genes in the classifier. Both in the 170 gene set and the top 30 gene subset, the proportion and average importance scores were similar, and no significant differences were detected, indicating equally important roles of immune and metabolic genes in constructing the classifier (**Supplementary Figures 2A–D**).

To summarize immunometabolism characteristics of the tumor microenvironment, we performed consensus clustering for TCGA cohorts. The optimal cluster of three was estimated by the consensus clustering matrix and NbClust test (**Supplementary Figures 1B–E**). Patients (374) were divided into three clusters, namely C1, C2, and C3 (**Figure 1C**), and survival analysis was performed. Results indicated that overall survival (OS) and disease-free survival (DFS) differed significantly among these subtypes (OS, C1 *vs* C2: *P* < 0.0001, C1 *vs* C3: *P* < 0.05, C2 *vs* C3: *P* < 0.001; median OS, C1: 39.0, C2: 65.5, C3: 43.9; DFS, C1 *vs* C2: *P* < 0.05, C1 *vs* C3: ns, C2 *vs* C3: *P* < 0.05; median DFS, C1: 16.8, C2: 19.1, C3: 15.1). C2 exhibited the best survival among subtypes in OS (C1, HR: 1.70, 95% CI: 1.29–2.25; C2: HR: 0.45, 95% CI: 0.32–0.62; C3: HR: 1.22, 95% CI: 0.94–1.58) and DFS (C1, HR: 1.19, 95% CI: 0.90–1.57; C2: HR: 0.67, 95% CI: 0.50–0.88; C3: HR: 1.24, 95% CI: 0.96–1.62), followed by C1 and C3 (*P* < 0.05) (**Figures 1D, E**). Furthermore, we validated the repeatability of our clustering results with the expression profiles of validation cohort 1 and 2 (**Supplementary Figures 2E–H**). Results showed that there were significant differences in prognosis among subtypes in validation cohort 1 (*P* < 0.001, C1: median OS: 27, HR: 1.35, 95% CI: 1.07–1.70; C2: median OS: 45, HR: 0.63, 95% CI: 0.48–0.81; C3: median OS: 37, HR: 1.13, 95% CI: 0.91–1.39) (**Figure 1F**) and validation cohort 2 (*P* = 0.015, C1: median OS: 15, HR: 1.26, 95% CI: 0.69–2.30; C2: HR: 0.21, 95% CI: 0.07–0.68; C3: median OS: 90, HR: 1.63, 95% CI: 0.93–2.86) (**Figure 1G**). Regarding the prognostic independence of the subtypes, multivariate Cox regression analysis was performed on the TCGA cohort with multiple factors, including age, clinical stage, and immunometabolism subtype. Results showed that immunometabolism subtypes were independent prognostic factors for ovarian cancer (C1 and C3 vs. C2, HR = 2.225, 95% CI: 1.603–3.089, *P* < 0.001) (**Supplementary Table S4**).

We also found the differentially expressed immune and metabolic genes among immunometabolism subtypes at the protein expression level. Immunohistochemistry (IHC) from the human protein atlas was used to explore the protein expression of 19 of the top 30 immunometabolism genes in ovarian cancer. Cluster analysis using the hclust algorithm showed that 13 patients were divided into three clusters (**Supplementary Figure 3A**). Immune proteins (IFNG, HLA-DOB) were significantly overexpressed in C1 and metabolic proteins (GALNT10, POLR1A) were significantly overexpressed in C1 and C2, while we observed low expression levels of immune and metabolic proteins in C3 (**Supplementary Figure 3B**).

We analyzed the DEGs among subtypes to explore the biological characteristics of the three subtypes (**Supplementary Table S5**). Gene set enrichment analysis was performed with the MsigDB Hallmark, KEGG, and Gene Ontology (GO) gene sets for DEGs. We observed upregulation of immune activation pathways in C1 and C2, including inflammatory response pathways, chemokine signaling pathways, and antigen presentation pathways, whereas these were downregulated in C3. Interestingly, oncogenic pathways were also upregulated in C1 and C3, including epithelial-mesenchymal transition pathway, cell adhesion pathway, and MYC and WNT pathways (**Figures 1H, I**, **Supplementary Figures 4A, B**, and **Supplementary Table S6**). Taken together, we conducted a comprehensive immunity and metabolism assessment of ovarian cancer patients, and we proposed an immunometabolism clustering of ovarian cancer with prognosis implications.

### Characterization of Immune Microenvironments of Immunometabolism Subtypes

Considering different activation levels of immune response pathways among subtypes in function enrichment analysis, we estimated the immune microenvironment and anti-tumor immune response levels in three subtypes. In general, the anti-tumor immune response requires several steps (37), and we performed analysis from four aspects: immune cell infiltration; antigen presentation pathway, expression level of co-stimulator and co-inhibitor molecules; immune response-related cytokines (38).

**Figure 2:**
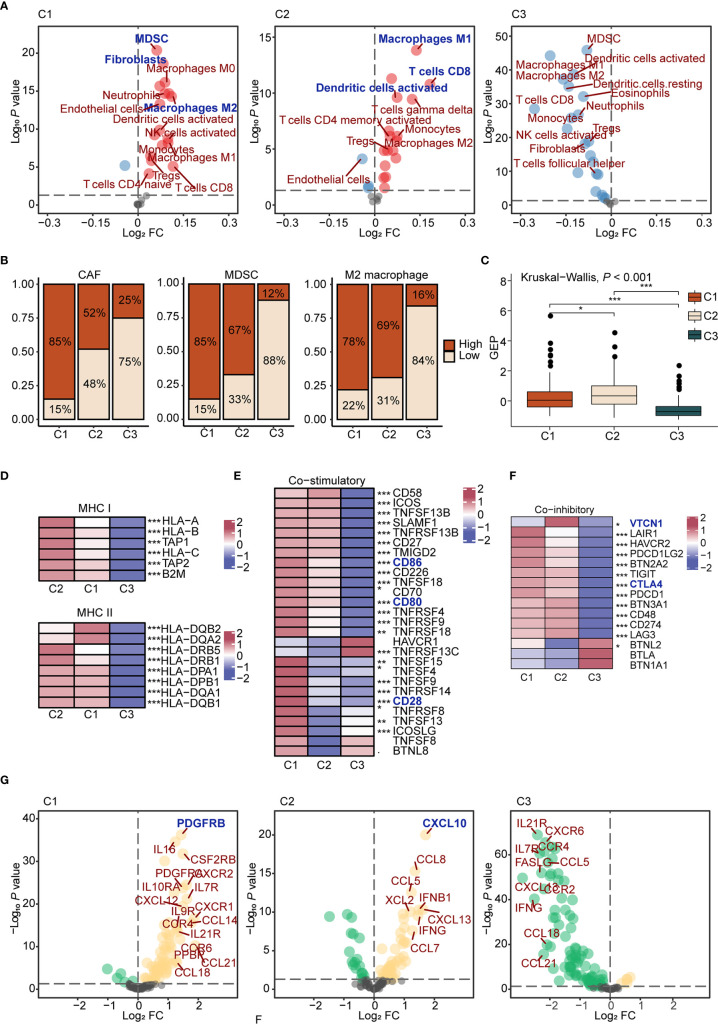
The characteristics of immune microenvironment of immunometabolism subtypes **(A)** The volcano plots of enriched and depleted immune and stromal cells for each subtype compared with other subtypes with limma package. Red: enriched; Blue: depleted. **(B)** Patients were classed according to the median infiltration scores of immunosuppressive cells (CAFs, MDSC, and M2 macrophages), and the percentages of the high score group and the low score group were calculated. **(C)** GEP scores differences in three subtypes. The differences were compared using the Kruskal-Wallis test. **(D)** Differences of MHC molecules expression level in three subtypes. The differences among each subtype were compared by Kruskal-Wallis test. **(E, F)** Differences of co-stimulatory **(E)** and co-inhibitory **(F)** molecules expression level in three subtypes. **(G)** The volcano plots of enriched and depleted expression of chemokines and cytokines for each subtype compared with other subtypes with limma package. Yellow: enriched; Green: depleted. The differences among each subtype were compared by Kruskal-Wallis test. (*P < 0.05; **P < 0.01; ***P < 0.001).

First, we estimated the abundance of 25 cells using the ssGSEA algorithm in each sample of TCGA ovarian cancer cohort, and comparisons were carried out among subtypes. Results showed that C1 displayed high infiltration of innate immune cells, such as M0 macrophages and dendritic cells, and immunosuppressive cells, like CAFs, MDSCs, and M2 macrophages, while C2 displayed abundant adaptive immune cell infiltration, such as M1 macrophages and CD8^+^ T cells. Besides, C3 was characterized by low infiltration of immune and stromal cells (**Figures 2A, B**). To further interrogate the function of T cells, we evaluated IFN-γ-related gene expression profiles (GEP) in each subtype. The GEP score in C2 was higher than other subtypes (*P* < 2.2e-16; **Figure 2C**), suggesting T cell activation and effective immune response in C2 (39, 40). Thus, C1 was preliminary considered as an immune-suppressive subtype, with C2 as the immune-inflamed subtype, and C3 as the immune-desert subtype. Second, we analyzed the expression of antigen presentation molecules. Expression of MHC molecules in C3 was significantly lower than other subtypes (*P* < 0.001, **Figure 2D**, and **Supplementary Figure 5A**), which may dampen the immune response (41), while the immune-desert microenvironment was observed in C3.

Regarding co-stimulatory molecules, significantly lower expressions were detected in C3 compared to other subtypes, such as CD80 (*P* < 0.001), CD86 (*P* < 0.001), and CD28 (*P* < 0.001) (**Figure 2E**), which were confirmed as important molecules delivered by antigen presenting cells to expand T cell activation. In addition, high expression levels of co-inhibitory molecules, such as CTLA4 and VTCN1, were found in C2 (**Figure 2F**), suggesting that C2 may benefit from immune checkpoint inhibitors (42). Finally, differences regarding cytokines and chemokines among groups were analyzed. In C1, various cytokines were upregulated, such as CCL4, TNF, and IL6, which could be recognized as a cytokine storm (43). Previous reports showed that a cytokine storm may lead to immune tolerance after counteracting between positive and negative mediators. For example, a cytokine storm induced by M2 macrophages upon new-adjuvant treatment in ovarian cancer promotes tumor growth and progression (44). As for specific upregulated cytokines, we found that PDGFRB, a molecular marker of CAFs (45), was highly expressed in C1 (*P* < 0.001). CXCL10, upregulated in C2 (*P* < 0.001), is a pro-inflammatory cytokine involved in the chemotactic recruitment of macrophages, natural killer cells, dendritic cells, and active T lymphocytes to tumor cells (46). Cytokine expression levels were low in C3, in accordance with the immune-desert tumor microenvironment (**Figure 2G** and **Supplementary Figure 5B**).

Considering the importance of microenvironment cells to prognosis, we next explored the prognostic significance of immune and stromal cells both in the entire cohort and in each subtype (**Supplementary Table S7**). High immune cell infiltration played a protective role regarding prognosis in the entire cohort. Interestingly, different cells diversely influenced the prognosis among three subtypes. For example, M1 macrophages played a protective role in C2 (HR = 0.60, *P* < 0.05, 95% CI, 0.38–0.94), which promotes immune response. M2 macrophages (HR = 1.50, *P* < 0.01, 95% CI, 1.13–1.98) reportedly exert pro-tumorigenic functions by inhibiting the immune response (47), which predicted worse prognosis in C3 (**Supplementary Figure 5C**).

In summary, we assessed the immune microenvironment and identified C1 as the immune suppressive subtype, characterized by infiltration of innate immune cells, immunosuppressive cells, and a cytokine storm, C2 as the immune inflamed subtype, which features adaptive immune cell infiltration, and C3 as the immune desert subtype, which displayed low infiltration of microenvironment cells and reduced expression of antigen presentation molecules.

### Metabolic Reprogramming Patterns Among Subtypes and Interactions With Immune Infiltration

**Figure 3:**
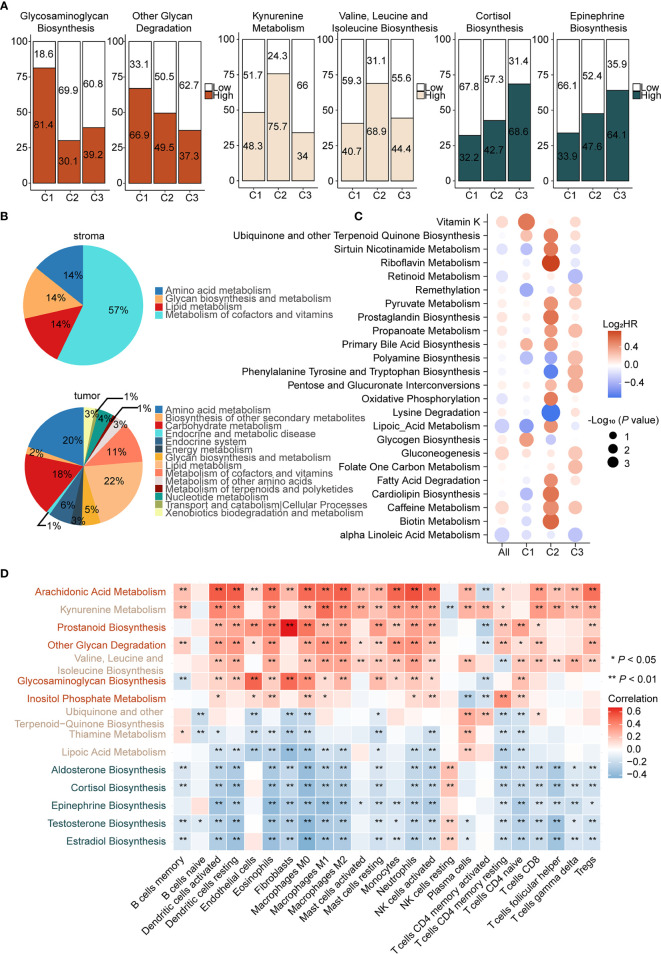
Different pattern of metabolism reprogramming among subtypes and its relationship with immune infiltration **(A)** Enrichment scores of metabolic pathways were calculated by ssGSEA and then the differential analysis was performed among three subtypes. Significantly upregulated metabolic pathways were selected based on Log2 (fold change). Patients were classed according to the median enrichment scores of featured metabolic pathways of each subtype, and the percentages of the high and the low score group were calculated (left: C1, middle: C2, right: C3). **(B)** Enrichment scores of metabolic pathways of stroma and tumor samples in GSE115635 were calculated and then the differential analysis was performed (FDR < 0.05 and Log2 (fold change) > 0 was consider significant). Significantly upregulated metabolic pathways were classified into metabolic patterns defined by KEGG. The upregulated metabolic pathways belonging to each category, divided by the total upregulated metabolic pathways, are the percentages of the metabolic pattern in pie charts. **(C)** Prognostic value of metabolic pathways estimated by the univariate Cox proportional hazards model for OS in TCGA cohort and each subtype. The color represents the hazard ratio, the size of circles represents -Log10 (*P* value). **(D)** Spearman correlation matrix of characteristic metabolic pathways and enrichment scores of immune cells in the whole cohort. Correlation coefficients are represented in the form of heatmap using colored scale ranging from blue (minimum correlation) to red (maximum correlation). (*P < 0.05; **P < 0.01).

Metabolic factors are important attributes for distinguishing subtypes. In the above analysis, immunometabolism subtypes displayed distinctive immune microenvironments and previous studies have demonstrated that metabolic flux could influence immune infiltration (15). However, the specific regulatory relationship between metabolic factors and immune infiltration remains to be explored. To gain insights into metabolic heterogeneity among subtypes, we calculated enrichment scores of 113 metabolic pathways from the KEGG and differential analysis was subsequently performed among subtypes. Furthermore, we ranked the differential metabolic pathways of each subtype according to Log2 (fold change) and selected the top five metabolic pathways. We found that glycosaminoglycan biosynthesis and other glycan degradation were significantly upregulated in C1 (**Figure 3A** left and **Supplementary Figure 6A**), which belongs to glycan metabolism pathways. In C2, kynurenine metabolism and valine, leucine, and isoleucine biosynthesis, from amino acid metabolism, were upregulated (**Figure 3A** middle and **Supplementary Figure 6B**). In C3, testosterone, estradiol, and epinephrine biosynthesis, pertaining to hormone metabolism, were specifically upregulated (**Figure 3A** right and **Supplementary Figure 6C**). Therefore, we considered C1 as the “glycan metabolism subtype,” C2 as the “amino acid metabolism subtype,” and C3 as the “endocrine subtype.”

Increasing evidence suggested that metabolic reprogramming participated in the regulation of immune cell infiltration and function. The metabolic activity of tumor cells has an important role in shaping the immune microenvironment. Notably, the effect of stromal cells cannot be ignored (48). To assess the role of the stroma and tumor tissue in metabolic reprogramming, we calculated enrichment scores of 113 metabolic pathways of the laser micro-dissected cancer-associated stroma and tumor samples of ovarian cancer in GSE115635 (49). Thereafter, differential analysis was performed between stroma and tumor samples and the significantly upregulated metabolic pathways were classified into metabolic patterns based on KEGG. In the stroma tissue, metabolism of cofactors and vitamins, lipid metabolism, amino acid metabolism, and glycan biosynthesis and metabolism patterns were upregulated (**Figure 3B** up). In the tumor tissue, lipid metabolism, amino acid metabolism, carbohydrate metabolism, metabolism of cofactors and vitamins, and endocrine system were upregulated (**Figure 3B** down). Though it appears that amino acid metabolism pathways were shared by tumor and stroma tissues, differential analysis showed 95% (20/21) of these were significantly upregulated in tumor tissues compared with stroma tissues (**Supplementary Figure 6C**). Therefore, we speculated that upregulation of amino acid metabolism pathways in C2 and endocrine metabolism in C3 may be mediated by tumor cells. On the contrary, our results indicated that glycan biosynthesis and metabolism pathways were primarily upregulated in stromal cells, which suggested C1 as the stroma-abundant subtype. This is also consistent with CAF infiltration in C1, as CAFs reportedly produced ECM proteoglycans through glycan metabolism (50).

Given the close relationship between metabolic reprogramming and immune cell infiltration, correlations between featured metabolic pathways and infiltration of immune and stromal cells were analyzed (**Figure 3D**). First, glycosaminoglycan biosynthesis, upregulated in C1, displayed significant positive correlations with endothelial cells (*P* < 0.001, r = 0.51), CAFs (*P* < 0.001, r = 0.49) and M0 macrophages (*P* < 0.001, r = 0.38), suggesting an immune suppressive phenotype in C1 was correlated with activation of glycosaminoglycan biosynthesis. Second, there were significant positive correlations between kynurenine metabolism, a featured pathway of C2, and multiple immune cells, such as dendritic cells (*P* < 0.001, r = 0.40) and M1 macrophages (*P* < 0.001, r = 0.48). Studies reported that *IDO*, the key gene of kynurenine metabolism, accompanied by an immune inflammatory response, may be an immune escape mechanism by tumors after sensing IFN-γ (51). Third, significant negative correlations were found between epinephrine biosynthesis, a featured pathway of C3, and multiple immune cells, such as dendritic cells (*P* < 0.001, r = −0.33) and M1 macrophages (*P* < 0.001, r = −0.37), which suggested that epinephrine biosynthesis may contribute to the immune desert phenotype.

We next determined the prognosis value of metabolic pathways in ovarian cancer (**Supplementary Table S8**). Univariate Cox analysis was employed to screen metabolic pathways with prognostic value (HR > 1.2 or HR < 0.8 and *P* < 0.05) across subtypes. Considering there were various pathways with significant prognostic value, two major criteria were chosen to select prognostic-associated metabolic pathways, namely metabolic pathways distinctly activated in each subtype and important oncogenic pathways reported in previous studies were also considered. The prognostic metabolic pathways are further elucidated below. In C1, glycogen biosynthesis, a featured metabolic pathway of C1, was associated with poor prognosis (HR = 1.33, *P* < 0.05, 95% CI, 1.03–1.73). In C2, two metabolic pathways were associated with prognosis. Lysine degradation was a protective factor (HR = 0.61, *P* < 0.01, 95% CI, 0.46–0.82) and oxidative phosphorylation was a risk factor (HR = 1.46, *P* < 0.05, 95% CI, 1.02–2.12). Lysine degradation exhibited the lowest HR in univariate Cox regression analysis. Lysine is associated with various carcinogenic pathways and further lead to tumor proliferation. Therefore, lysine degradation is a protective factor in C2, consistent with previous reports. As for oxidative phosphorylation, it has been reported that inhibition of oxidative phosphorylation could improve hypoxia and increase treatment effects (52). Finally, propanoate metabolism (HR = 1.22, *P* < 0.05, 95% CI, 1.01–1.49) was significantly upregulated in C3, despite not being a characteristic metabolic pathway of C3 (**Figure 3C**). However, it has been reported that the propanoate metabolism pathway may play an important role in TSA inhibition in gastric cancer and could be a potential therapeutic target for it (53).

In short, we assessed metabolic differences among subtypes and considered C1 as the immune suppressive-glycan metabolism subtype, C2 as the immune inflamed-amino acid metabolism subtype, and C3 as the immune desert-endocrine subtype. Metabolism-immune microenvironment interactions were further elucidated. Glycosaminoglycan biosynthesis displays a positive correlation with CAFs in C1, activation of kynurenine metabolism was synchronized with immune inflamed microenvironment in C2, and hormone metabolism correlated with the desert phenotype in C3.

### Activation of Epinephrine Biosynthesis Correlates Inversely With Expression of MHC Molecules and Antigen Presentation

**Figure 4:**
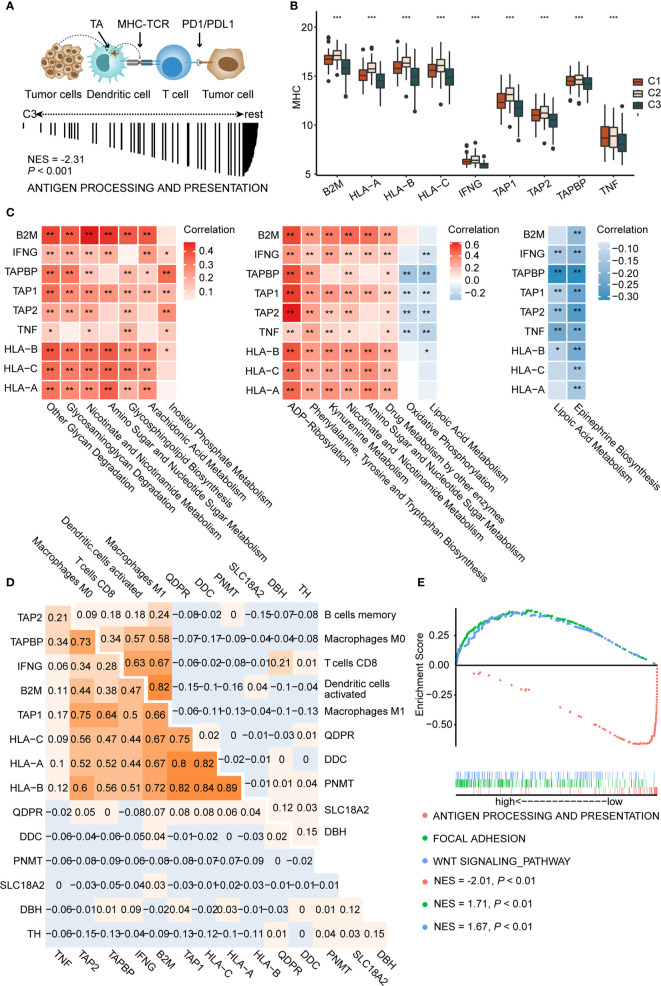
Activation of epinephrine biosynthesis correlates inversely with expression of MHC molecules and antigen presentation **(A)** Schematic diagram of antigen presentation. First, tumor antigens released by tumor cells are captured by dendritic cells. Next, dendritic cells present the captured antigens on MHC-I molecules to T cells, leading to activation of T cells. Activated T cells infiltrate the tumor bed and kill tumor cells. TA, tumor antigen (**A**, up). GSEA plot showing the negative enrichment of antigen processing and presentation in C3 compared to C1 and C2 (**A**, down). **(B)** The boxplot of MHC molecules in three subtypes. *P* values are the results of Kruskal-Wallis test for three subtypes. **(C)** Correlation matrix of characteristic metabolic pathways of each subtype and expression of MHC-I molecules (left, C1, middle, C2, right, C3.) Correlation coefficients are represented in the form of heatmap using colored scale ranging from blue (minimum correlation) to red (maximum correlation). **(D)** Correlations between genes in epinephrine biosynthesis pathway and MHC-I molecules (**D**, left) and antigen presentation cells (**D**, right) were determined by Spearman correlation analysis. The numbers are the correlation coefficient. **(E)** Grouping the patients in the TCGA cohort according to the median expression of TH. GSEA plot showing the negative enrichment of antigen processing and presentation in the high TH expression group compared with low TH expression group. (*P < 0.05; ** *P* < 0.01; ***P < 0.001).

Antigen presentation, the key step in the cancer immunity cycle (37), starts by capturing and processing new tumor antigens, and then presents tumor antigens bound with MHC peptides to T cells, initiating the effector T cell response against tumor-specific antigens (**Figure 4A** above) (54). Antigen presentation genes were downregulated in C3 (NES = −2.31, *P* < 0.001, **Figure 4A** below) compared to C1 and C2 in GSEA. MHC molecules are functional executants of antigen presentation (54). The expression of MHC molecules was analyzed in each subtype and results indicated reduced expression of these in C3 (*P* < 0.001), consistent with the above-mentioned results, suggesting that antigen presentation might be impaired (**Figure 4B**).

Recently, David O’Sullivan’s research found that the process of antigen presentation may be affected by metabolites of the tumor microenvironment (55). For example, the accumulation of lactic acid in the tumor microenvironment could interrupt the maturation of dendritic cells (15). Therefore, we speculated that antigen presentation differences among each subtype may be partially attributed to metabolic reprogramming. We conducted correlation analysis to identify the metabolic pathways that may affect the expression of MHC molecules. Top 30 differential metabolic pathways among subtypes were included. Metabolic pathways, such as other glycan degradation (with HLA-B, *P* < 0.001, r = 0.39), glycosaminoglycan degradation (with HLA-A, *P* < 0.001, r = 0.33), and kynurenine metabolism (with HLA-B, *P* < 0.001, r = 0.35), display significant positive correlations with MHC molecules (**Figure 4C** left and middle). Interestingly, we found that epinephrine biosynthesis, a featured pathway of C3, negatively correlated with most MHC molecules, such as HLA-A (*P* < 0.01, r = −0.14), HLA-B (*P* < 0.001, r = −0.22), and HLA-C (*P* < 0.01, r = −0.15) (**Figure 4C** right). Furthermore, correlation analysis also revealed that epinephrine biosynthesis negatively correlated with APCs, including M1 macrophages (*P* < 0.001, r = −0.37) and dendritic cells (*P* < 0.001, r = −0.33) (**Supplementary Figures 7A, B** and **Supplementary Table S9**).

We next tested genes in the epinephrine biosynthesis pathway for correlation with both MHC and APCs. Among them, the *TH* gene displayed significantly negative correlations with HLA-B (*P* < 0.05, r = −0.11), HLA-C (*P* < 0.05, r = −0.12), and M1 Macrophages (*P* < 0.01, r = −0.13) (**Figure 4D** and **Supplementary Figure 7C**). Additionally, GSEA analysis showed reduced antigen presentation (NES = −2.01, *P* < 0.01) and enriched WNT (NES = 1.71, *P* < 0.01) and focal adhesion (NES = 1.67, *P* < 0.01) in the high *TH* group compared to the low TH group, suggesting that antigen presentation pathways were negatively correlated with *TH* expression (**Figure 4E**). Based on the results above, we proposed that over-expression of TH may participate in suppressing antigen presentation and activation of T cells, which may consequently inhibit tumor immune response. In total, we found key metabolic factors in the regulation of antigen presentation. Upregulation of the epinephrine biosynthesis pathway, especially the *TH* gene, exhibited a significant negative correlation with MHC molecules and APCs, which may restrict an effective immune response.

### Immunometabolism Subtypes Can Predict the Treatment Efficacy and Survival of Ovarian Cancer Patients

**Figure 5:**
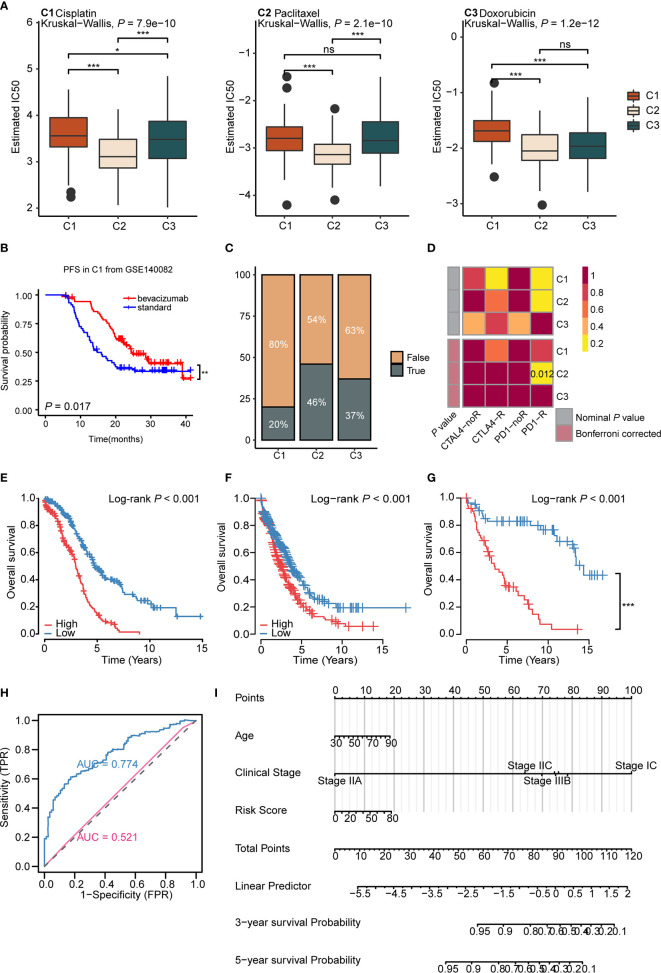
Immunometabolism subtypes could predict the treatment efficacy and survival of ovarian cancer patients **(A)** The boxplots of the estimated IC50 for cisplatin, paclitaxel, and doxorubicin for three subtypes based on GDSC database. *P* values were calculated using Kruskal-Wallis test for three subtypes. **(B)** Kaplan-Meier curves of progression free survival (PFS) for bevacizumab *versus* standard treatment stratified in C1 from GSE140082 (*P* < 0.05). **(C)** The predicted response rate of immunotherapy (TRUE/FALSE) to anti-PD-L1 among three subtypes in the TCGA ovarian cancer cohort. Fisher exact test, *P* < 0.001. **(D)** Submap analysis of the response to PD-L1 inhibitor among three subtypes. (Bonferroni corrected *P* value: 0.012). **(E–G)** Kaplan-Meier curves of OS for high and low risk score group in the TCGA ovarian cancer cohort **(E)**, validation cohort 1 **(F)**, validation cohort 2 **(G)**. **(H)** ROC curves measuring the predictive value of risk score and clinical stage. The area under the ROC curve was 0.774 and 0.521 for the risk score and clinical stage, respectively. **(I)** The nomogram for predicting probability of survival at 3 and 5 year in patients. (*P < 0.05; **P < 0.01; ***P < 0.001, ^ns^ *P* > 0.05).

Drug therapy for ovarian cancer includes chemotherapy, targeted therapy, and immunotherapy. Chemotherapy is first-line treatment for ovarian cancer, while targeted therapy is usually used for maintenance therapy (56). However, immunotherapy, such as immune checkpoint inhibitor monotherapy, still displays a poor complete response rate (~10%) and requires further exploration (9). As for chemotherapy, cisplatin, paclitaxel, and doxorubicin are important first-line drugs for ovarian cancer (3, 57, 58). We used the predictive model of the three drugs based on Xiaofan Lu’s research to estimate the IC_50_ for each subtype (29). Results showed that C2 was more sensitive to chemotherapy compared to C1 and C3 (cisplatin, *P* < 2.2e-16; paclitaxel, *P* = 0.00061; doxorubicin, *P* = 1.2e-12) (**Figure 5A**).

Bevacizumab, the vascular endothelial growth factor (VEGF) inhibitor, is currently approved for maintenance therapy of ovarian cancer (59). We analyzed the validation cohort GSE140082 with bevacizumab treatment information. Results demonstrated that only C1 gained progression free survival (PFS) benefit from bevacizumab combined with chemotherapy (*P* = 0.017, bevacizumab: median OS: 24.8, HR: 0.58, 95% CI: 0.36–0.91; standard: median OS: 12.6, HR: 1.72, 95% CI: 1.72–2.70) (**Figure 5B** and **Supplementary Figures 8B, C**), suggesting us bevacizumab combined with chemotherapy treatment may improve the prognosis in C1 (**Supplementary Figure 8A**). The results above indicated that immunometabolism subtypes could screen potential groups that would benefit from bevacizumab combined with chemotherapy.

Though maintenance therapy for ovarian cancer has rapidly developed, patients who suffer from locally advanced or metastatic ovarian cancer rarely experience satisfying clinical outcomes (60). To identify potential groups that may benefit from immunotherapy, we used the TIDE algorithm to predict the response of immunotherapy in different subtypes (29). Results showed that C2 (46%) displayed a significantly better response to immunotherapy compared to C1 (20%) and C3 (37%) (*P* = 0.000184) (**Figure 5C**). The submap algorithm showed similar results. C2 displayed a higher response rate to PD-1 inhibitors (Bonferroni adjusted *P* = 0.012) (**Figure 5D**), suggesting that C2 may benefit from immunotherapy. Taken together, these data imply that the application of immunometabolism subtypes can identify potential groups that would benefit from immune checkpoint inhibitors. Simultaneously, for further verification, we used the Submap algorithm to predict the response of immunotherapy in GSE73614 cohort. Results showed C2 displayed a higher response to PD-1 inhibitors (Bonferroni adjusted *P* = 0.012) (**Supplementary Figure 8D**).

Furthermore, we established a risk signature including 27 genes (**Supplementary Table S10**), based on the immunometabolism gene set using the Lasso-Cox algorithm (27). The high-risk group exhibited a worse prognosis compared to the low-risk group (Log rank *P <*0.001, median OS: high risk: 2.98, low risk: 4.92; HR: 0.33, 95% CI: 0.25–0.48) (**Figure 5E**). Next, we validated the repeatability of our results with the expression profiles of validation cohort 1 (Log rank *P* < 0.001, median OS: high risk: 2.50, low risk: 3.75; HR: 0.65, 95% CI: 0.53–0.80) (**Figure 5F**) and validation cohort 2 (Log rank *P* < 0.001, median OS: high risk: 3.42, low risk: 14.33; HR: 0.16, 95% CI: 0.09-0.30) (**Figure 5G**). Further, the time-dependent area under the curve (AUC) demonstrated that the prognostic efficacy of the risk score was higher than the clinical stage (AUC: 0.775 *vs* 0.521) (**Figure 5H** and **Supplementary Figure 8E**). To further assess the application value of the above results, nomogram prediction for 3- and 5-year survival probability was established based on age, clinical stage, subtype, and risk score (**Figure 5I**). The calibration plot demonstrated good consistency between the prediction by nomogram and actual observation of the 3- and 5-year survival in ovarian cancer (**Supplementary Figure 8F**). In total, immunometabolism subtypes have predictive value for therapy stratification, especially in terms of chemotherapy and immunotherapy. In addition, we proposed a risk score and nomogram as new clinical prognostic indicators from the immunometabolism perspective.

## Discussion

It has been reported that tumor metabolism could affect immune cells and lead to immune evasion *via* local nutrient reduction and production of metabolic excreta (15). In this study, we defined an immunometabolism gene set of 170 genes based on Immport and KEGG databases, and then clustered patients into three subtypes, with validation on external datasets. The three immunometabolism subtypes displayed significant differences in prognosis, tumor microenvironment, efficacy of chemotherapy, and potential response to immunotherapy. Definition of immunometabolism subtypes of ovarian cancer may help unveil interactions between metabolic reprogramming and immune cell infiltration, putting forward new biomarkers for stratified prognosis and providing a new direction for screening out potential immunotherapy.

Immune phenotype can be stratified into three main types: immune inflamed type, characterized by the presence of CD4^+^ T cells and CD8^+^ T cells with myeloid cells and monocytes, immune suppressive type, characterized by the presence of innate immune cells and stromal cells, and immune desert type, characterized by the absence of immune cells (14). Research has shown that metabolism of tumor cells influences the function of immune cells, thereby affecting anti-tumor immune responses and promoting immune evasion (15). The immune subtype in ovarian cancer has provided insights into the tumor microenvironment (61). However, clustering based on the interplay between metabolic flux and immunology remains unclear. Here we presented immunometabolism subtypes of ovarian cancer and explored the relationship between immunity and metabolism to find potential therapeutic targets.

Glycosaminoglycan biosynthesis, kynurenine metabolism, and epinephrine biosynthesis were characteristic metabolic pathways of three subtypes, respectively. We found that metabolic characteristics of subtypes exhibited close relationships with immune phenotypes. Glycosaminoglycans, metabolites of glycosaminoglycan biosynthesis, are attached to the core protein to form proteoglycans (62). Proteoglycans, such as versican, are associated with recruitment of MDSCs (63), which may be the reason for high levels of MDSCs in the immune suppressive-glycan metabolism subtype. Kynurenine metabolism displayed positive correlation with APCs, including dendritic cells and M1 macrophages. It is reported that the kynurenine pathway is a regulator of adaptive immune responses and may serve as a negative feedback mediator of Th1 activation (51). Therefore, we speculated that the positive correlation between the kynurenine metabolism pathway and APCs may be due to the feedback mechanism. Interestingly, we found that epinephrine biosynthesis was one of the characteristic metabolic pathways of the immune desert-endocrine subtype and exhibited negative correlation with APCs. Further, epinephrine is a species of catecholamine. The primate ovary has a catecholamine-producing system for catecholamine biosynthesis. Panina-Bordignon et al. have shown that β-adrenergic receptors could promote Th2 cell development, which could suppress activated immunity (64). Thus, we considered that epinephrine biosynthesis upregulation may lead to the immune desert phenotype in C3.

Antigen presentation is a process including presentation of the MHC complex by APCs to naive T cells, and activation of CD8^+^ T cells (54). It is a key step in the tumor immune cycle. As a result, low or no expression of MHC molecules could lead to defective tumor antigen presentation. Excrescent metabolites produced by tumor cells may affect the expression of MHC molecules (65). It has been reported that adrenergic signaling can inhibit activation of T cells (66). Consistent with this, we found that MHC molecules were negatively correlated with epinephrine biosynthesis. We considered that epinephrine biosynthesis may reduce expression levels of MHC molecules in APCs, block antigen presentation, and subsequently cause inactivation of T cells. TH is the rate-limiting enzyme in the synthesis of catecholamines (67). Huang et al. reported that overexpressed TH promotes the differentiation of CD4^+^ T cells to Th2 cells (68), which suggested a possible role of the *TH* gene in regulating immune cell function. Notably, we found that *TH* was consistently negatively correlated with MHC molecules at the gene level, highlighting the importance of the *TH* gene in antigen presentation.

The first-line treatment of ovarian cancer includes cytoreductive surgery and platinum-based chemotherapy (56). Using GDSC, we considered that the immune inflamed-amino acid metabolism subtype may be more sensitive to commonly used chemotherapy drugs, which could lead to an improved prognosis. Analysis of the external validation cohort reveals bevacizumab combined with chemotherapy drugs could extend the PFS for patients in C1. Considering the important role played by angiogenesis in the tumor microenvironment (69), we speculated bevacizumab may improve the immune suppressive microenvironment in C1, which may provide favorable conditions for bevacizumab combined with immunotherapy. Based on two algorithms predicting immunotherapy efficacy, we considered that the immune inflamed-amino acid metabolism subtype displays a better response to immunotherapy. Thus, we speculated that the immune inflamed-amino acid metabolism subtype group may benefit from immunotherapy.

Overall, this study represents a novel perspective in ovarian cancer immunometabolism, and the subtypes could be applied to therapy and prognosis prediction. Further, we proposed an intimate relationship between epinephrine biosynthesis and the immune desert phenotype, and thus provide potential metabolic targets to reshape the immune microenvironment.

## Data Availability Statement

The data that support the findings of this study are available in TCGA, and GSE9891, GSE18520, GSE19829, GSE26193, GSE30161, GSE63885, GSE73614, GSE140082, and GSE115635 in GEO database and in The Human Protein Atlas. Data were derived from the following resources available in the public domain: http://cancergenome.nih.gov, https://cbioportal.org, https://www.ncbi.nlm.nih.gov/gds and http://www.proteinatlas.org.

## Author Contributions

MY designed, analyzed the data, and wrote the manuscript. YW conceptualized and developed an outline for the manuscript and revised the manuscript. GC and KG generated the figures and tables. All authors contributed to the article and approved the submitted version.

## Conflict of Interest

The authors declare that the research was conducted in the absence of any commercial or financial relationships that could be construed as a potential conflict of interest.
