# Supplementary material for: Tumor Immunometabolism Characterization in Ovarian Cancer With Prognostic and Therapeutic Implications
Source: Front Oncol. 2021 Mar 16;11:622752. doi: 10.3389/fonc.2021.622752 (PMC8008085; doi:10.3389/fonc.2021.622752)
Supplement: Supplementary file 1 [file DataSheet_1.doc]

**Supplementary Figure Legends**

**Figure S1 Consensus clustering of immunometabolism subtypes in TCGA ovarian cancer cohort**

A, The work flow of this study. B-C, Consensus matrixes of TCGA cohorts for each k (k = 2-3), exhibiting the cluster stability with 1000 iterations of consensus clustering. D, The relative change in area under the CDF curve of k = 2-6. E, NbClust analysis of ovarian cancer immunometabolism subtypes.

**Figure S2 The equally important roles of immune and metabolic genes in constructing the classifier and consensus clustering of immunometabolism subtypes in validation cohort 1 and validation cohort 2**

A, The importance scores of 170 immunometabolism gene set. Orange: immune genes, yellow: metabolic genes. B, Important scores have no differences between the 97 metabolic genes and 73 immune genes. The differences were compared using the Wilcoxon test. C, The importance scores of the top 30 immunometabolism gene subset. Orange: immune genes; yellow: metabolism genes. D: Important scores have no differences between the 15 metabolic genes and 15 immune genes. The differences were compared using the Wilcoxon test. E, Consensus matrixes of validation cohort 1 for k (k = 3), exhibiting the cluster stability with 1000 iterations of consensus clustering. F, The relative change in area under the CDF curve of k = 2-6 in validation cohort 1. G, Consensus matrixes of validation cohort 2 for k (k = 3), exhibiting the cluster stability with 1000 iterations of consensus clustering. H, The relative change in area under the CDF curve of k = 2-6 in validation cohort 2.

**Figure S3 Immunometabolism subtypes of ovarian cancer in protein level**

A, The heatmap of the immune and metabolic protein levels in 13 patients. B, The immune and metabolic protein levels in ovarian cancer tissues in The Human Protein Atlas database.

**Figure S4 Functional enrichment analysis of DEGs**

A-B, KEGG pathways from GSEA database were applied in enrichment analysis of DEGs in the three subtypes. Top 10 upregulated (A) or downregulated (B) pathways were demonstrated. Heatmap showed mean pathway scores constructed with GSVA algorithm.

**Figure S5 Characteristics of tumor immune microenvironment in ovarian cancer**

A, Prognostic value of immune cells infiltration estimated by the univariate Cox proportional hazards model for OS in the whole cohort and each subtype. B-C, Differences of other MHC molecules (B) and interferons and receptors (C) expression levels in three subtypes. The differences among each subtype were calculated with Kruskal-Wallis test. (*, *P* < 0.05; **, *P* < 0.01; ***, *P* < 0.001)

**Figure S6 Other featured metabolic pathways of subtypes**

A-C, Significantly upregulated metabolic pathways were selected based on Log_2_ (fold change). Patients were classed according to the median enrichment scores of featured metabolic pathways of each subtype, and the percentages of the high score group and the low score group were calculated in C1 (A), C2 (B) and C3 (C). D, The volcano plots of amino acid metabolism pathways in stroma and tumor tissues compared with each other with limma package. Red: upregulated; blue: downregulated.

**Figure S7 Correlation heatmap between characteristic metabolic pathways and MHC-I molecules and APCs.**

Correlations between characteristic metabolic pathways and MHC-I molecules (A) and antigen presentation cells (B) were determined by Spearman correlation analysis. Correlation coefficient are represented in the form of heatmap using colored scale ranging from blue (correlation coefficient = -1) to orange (correlation coefficient = 1).

C, Correlations between TH and 25 microenvironment cells. The size of each circle represents correlations calculated by Spearman correlation analysis and the color represents the *P* value.

**Figure S8 Immunometabolism subtypes could predict the treatment efficacy and survival of ovarian cancer patients.**

A, Kaplan-Meier curves of overall survival among the subtypes in GSE140082 cohort (Log-rank *P* = 0.037). B-C, Kaplan-Meier curves and log rank test of PFS for bevacizumab versus standard treatment stratified by immunometabolism subtypes from GSE140082 in C2 (B) and C3 (C). D, Submap analysis of the response to PD-L1 inhibitor among three subtypes. (Bonferroni corrected P value: 0.012) D, The curves of AUC of risk score changes with the increases in the number of included genes ranked by frequency. When number of genes reached 27, the AUC reached 0.775. E, The calibration curves demonstrated the agreement between the predicted and observed 3- and 5-year survival probability.
