# Supplementary figures and images for: Tumor Immunometabolism Characterization in Ovarian Cancer With Prognostic and Therapeutic Implications
Source: Front Oncol. 2021 Mar 16;11:622752. doi: 10.3389/fonc.2021.622752 (PMC8008085; doi:10.3389/fonc.2021.622752)

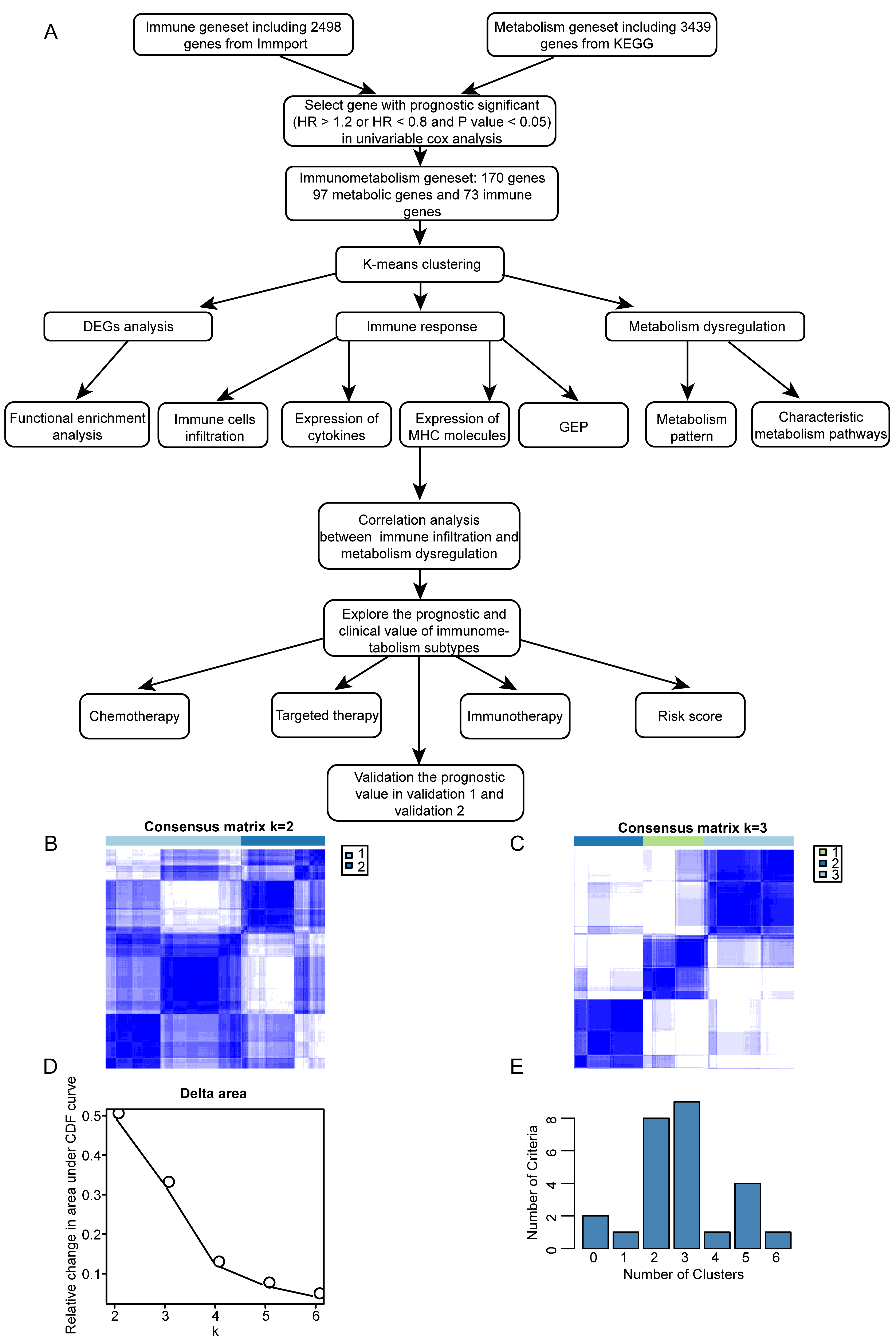

Supplement: Supplementary file 2 [file Image_1.tif]

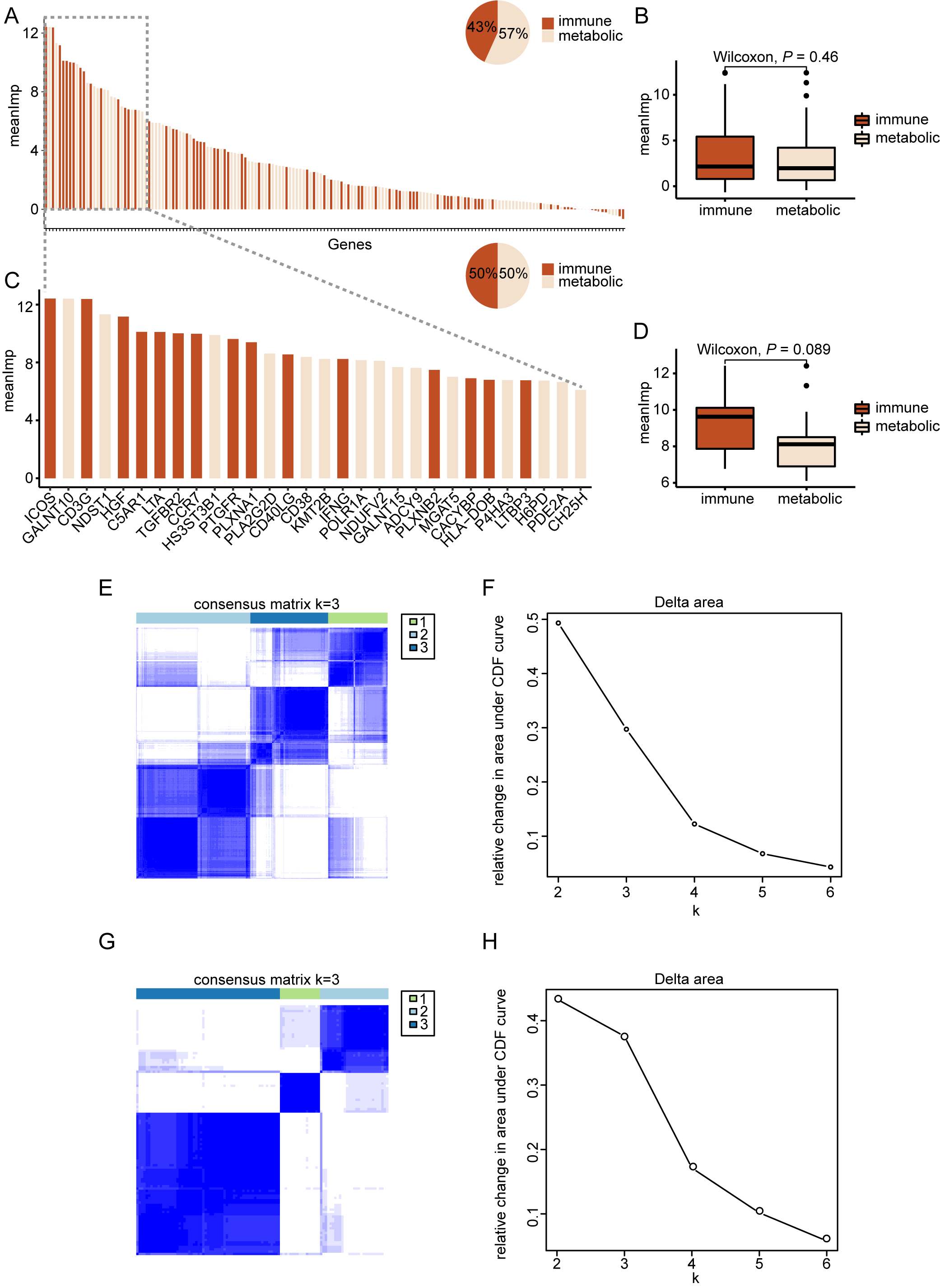

Supplement: Supplementary file 3 [file Image_2.tif]

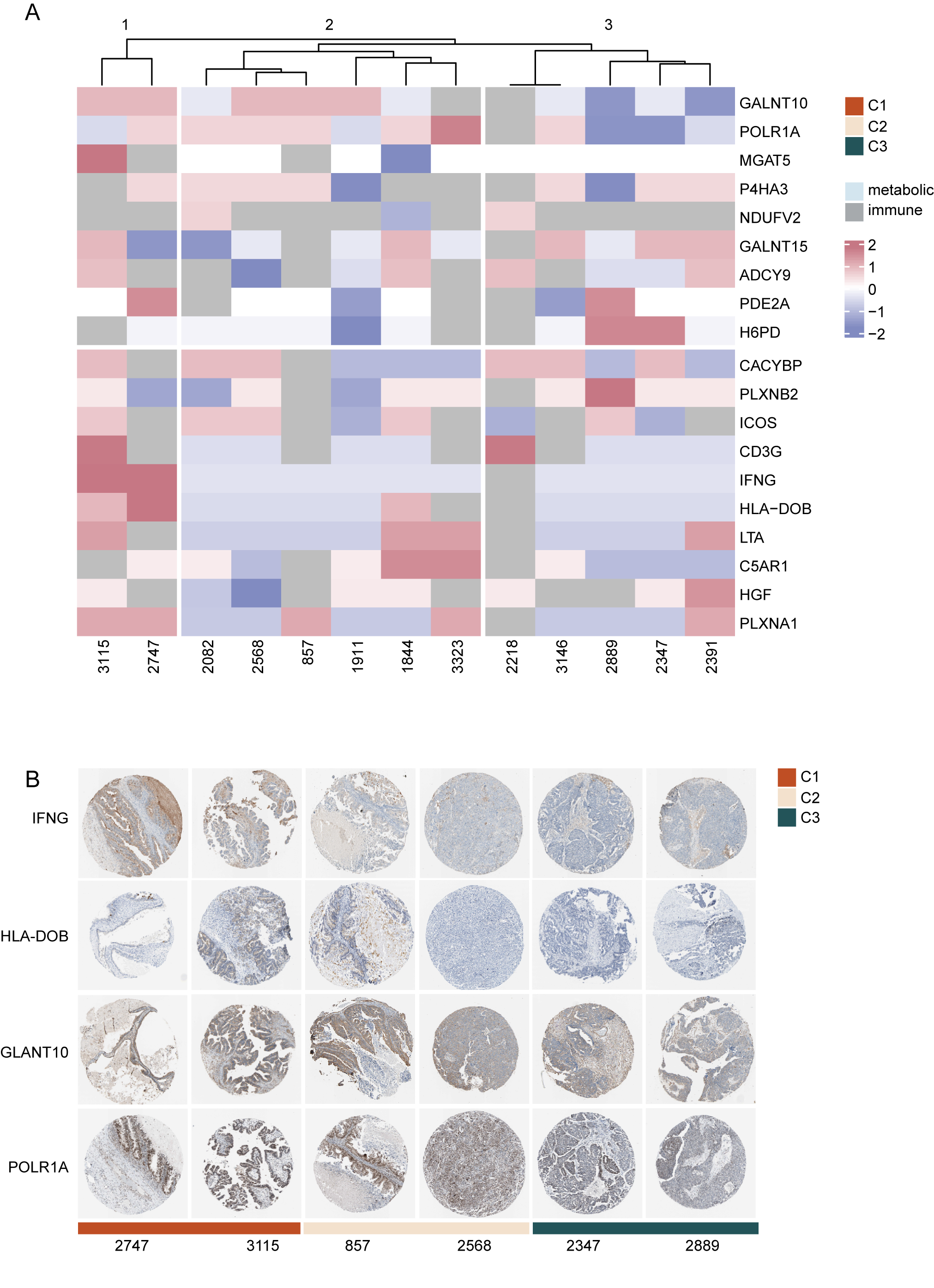

Supplement: Supplementary file 4 [file Image_3.tif]

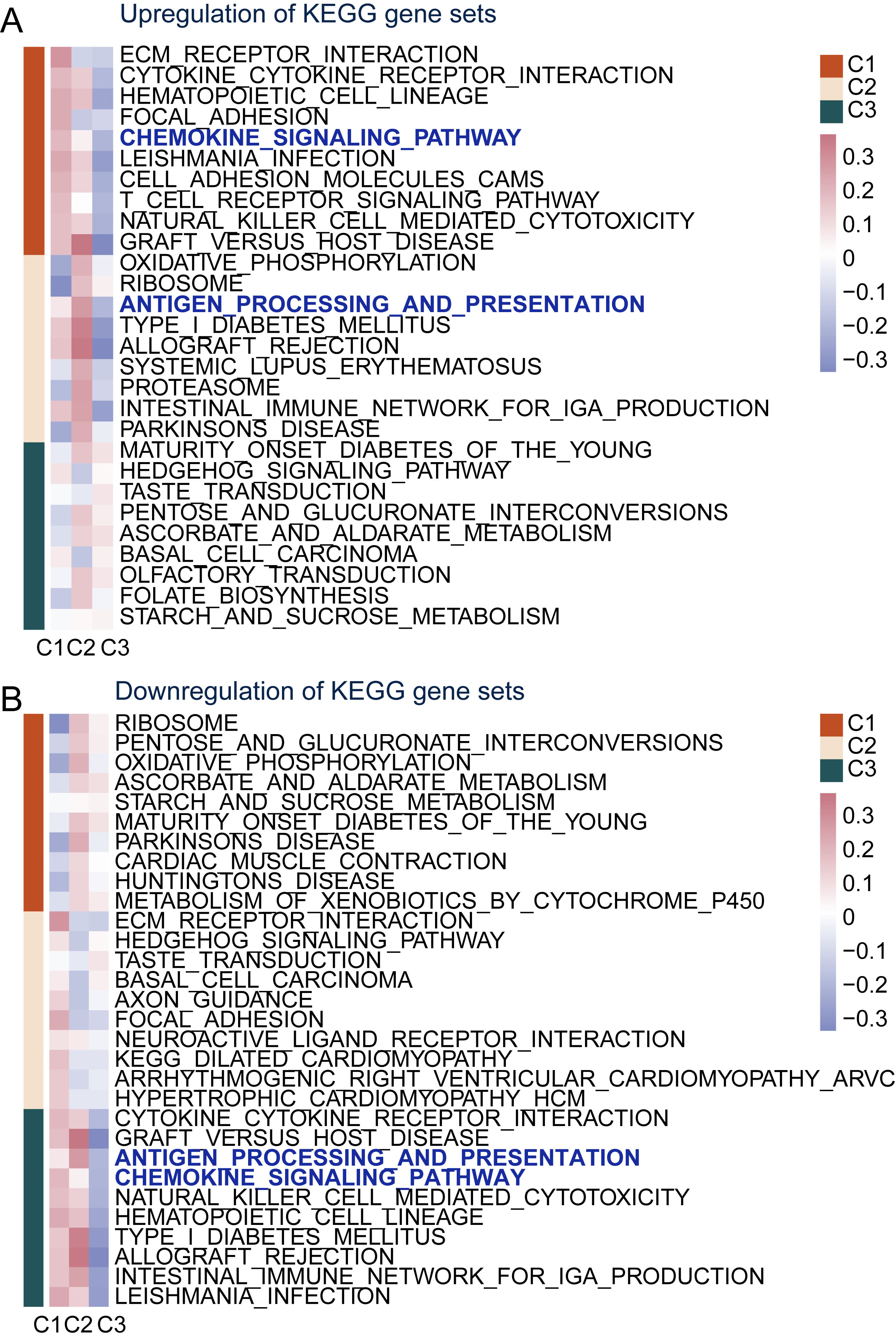

Supplement: Supplementary file 5 [file Image_4.tif]

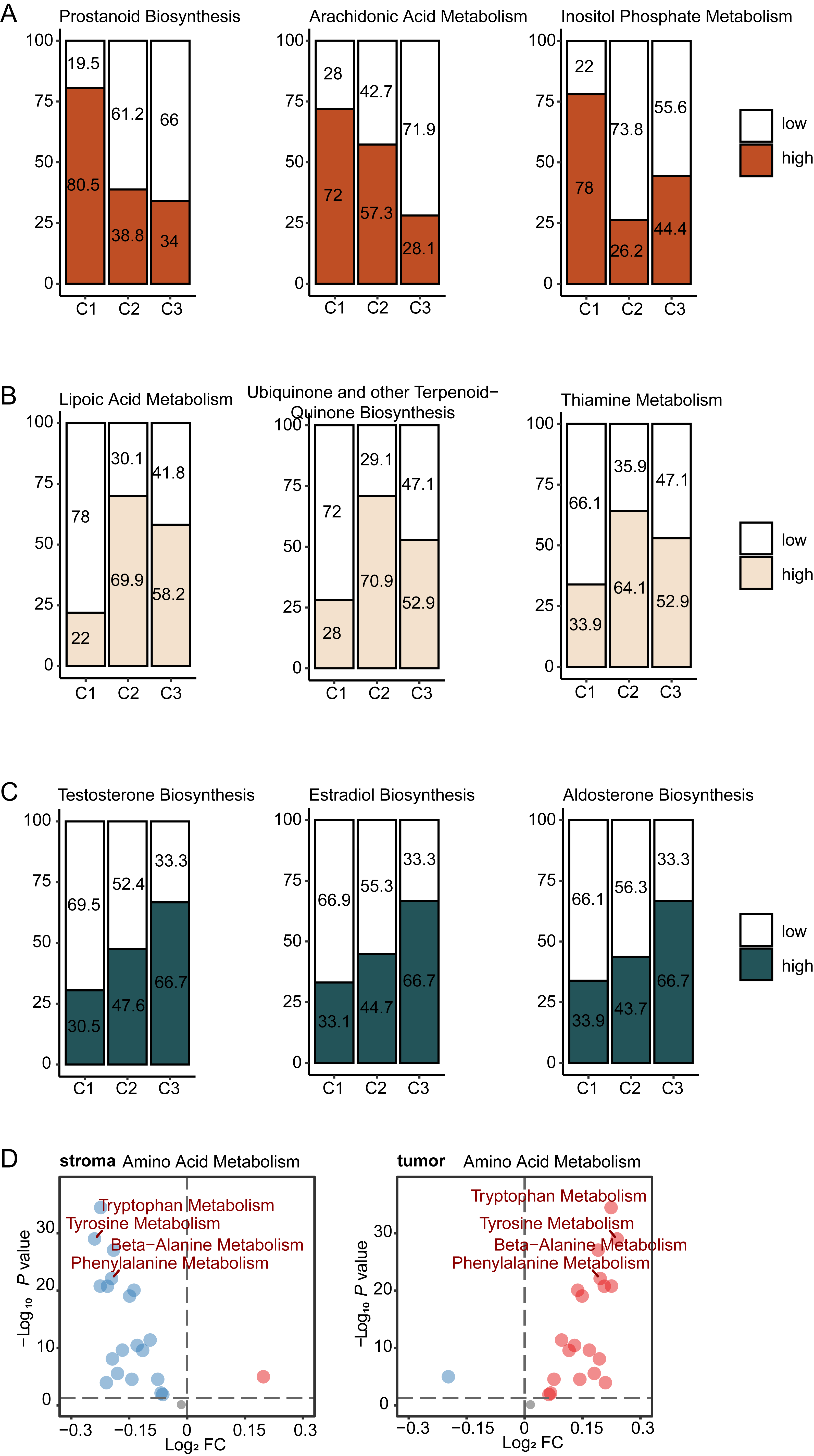

Supplement: Supplementary file 7 [file Image_6.tif]

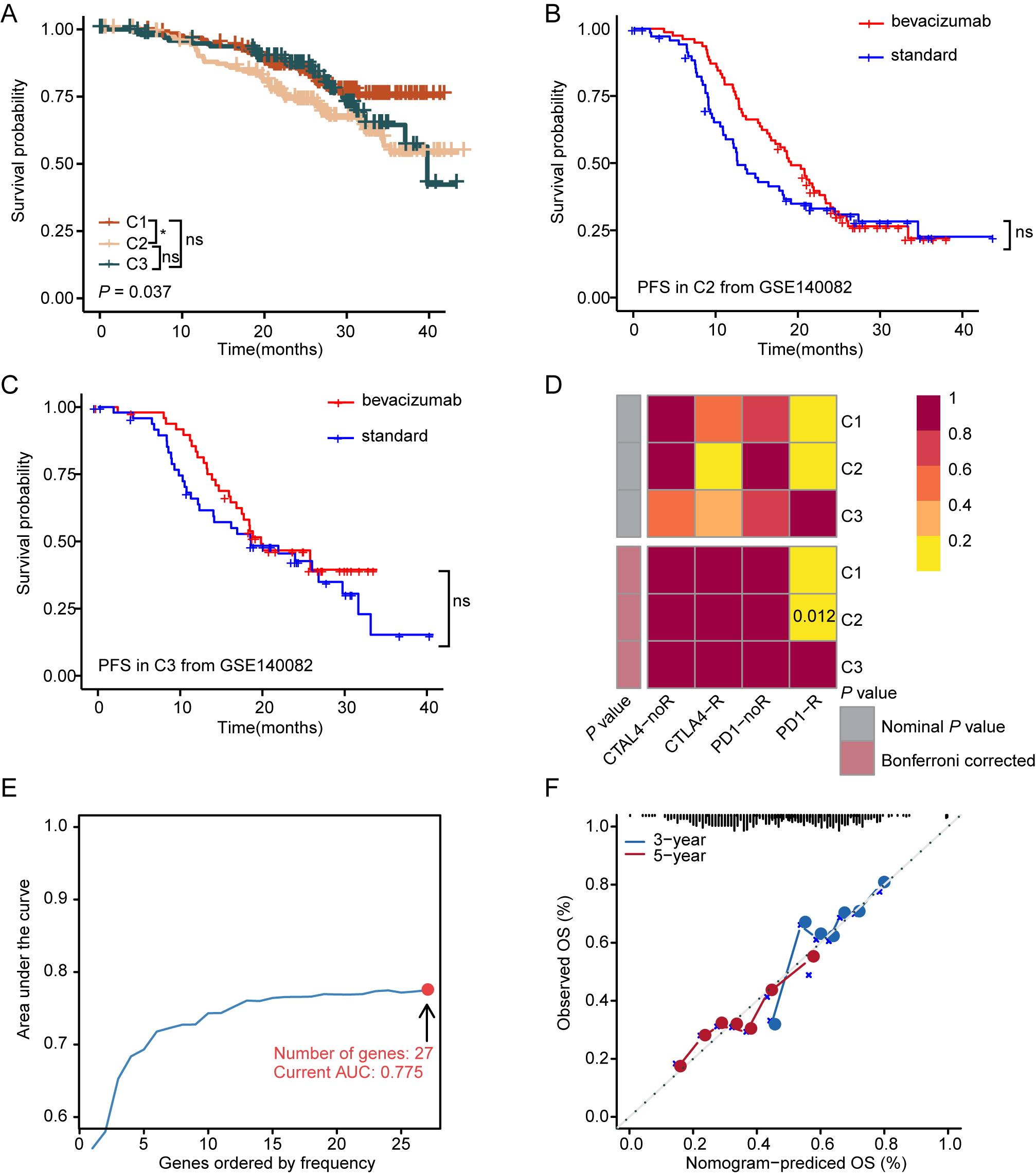

Supplement: Supplementary file 9 [file Image_8.tif]
