# Supplementary material for: Tumor Immunometabolism Characterization in Ovarian Cancer With Prognostic and Therapeutic Implications
Source: Front Oncol. 2021 Mar 16;11:622752. doi: 10.3389/fonc.2021.622752 (PMC8008085; doi:10.3389/fonc.2021.622752)
Supplement: Supplementary file 10 [file Table_1.doc]

**Supplementary Table S1: Data Source**

| **Dataset** | **Platform** | **Sample** | **Sample type** |
| --- | --- | --- | --- |
| TCGA | Affymetrix Human Genome U133A Array | 374 | tumor |
| GSE9891 | GPL570 | 280 | tumor |
| GSE18520 | GPL570 | 53 | tumor |
| GSE19829 | GPL570 | 28 | tumor |
| GSE26193 | GPL570 | 107 | tumor |
| GSE63885 | GPL570 | 75 | tumor |
| GSE73614 | GPL6480 | 107 | tumor |
| GSE140082 | GPL14951 | 380 | tumor |
| GSE115635 | GPL570 | 96 | tumor and stroma |

Validation cohort 1: GSE9891, GSE18520, GSE19829, GSE26193, GSE30161 and GSE63885 Validation cohort 2: GSE73614
