# Supplementary material for: Tumor Immunometabolism Characterization in Ovarian Cancer With Prognostic and Therapeutic Implications
Source: Front Oncol. 2021 Mar 16;11:622752. doi: 10.3389/fonc.2021.622752 (PMC8008085; doi:10.3389/fonc.2021.622752)
Supplement: Supplementary file 11 [file Table_2.doc]

**Supplementary Table S2: The prognostic value of immune and metabolic genes in TCGA OV OS**

| **The prognostic value of immune genes** | | | |
| --- | --- | --- | --- |
| **Gene** | **HR (95%CI)** | **z** | ***P* value** |
| HLA-DOB | 0.7618(0.6609-0.8781) | -3.7533 | 2.00E-04 |
| CD40LG | 0.6355(0.484-0.8345) | -3.2619 | 0.0011 |
| ANGPT4 | 1.4839(1.1701-1.882) | 3.2554 | 0.0011 |
| CD3G | 0.7764(0.6612-0.9116) | -3.0894 | 0.002 |
| PTH | 0.218(0.08-0.5941) | -2.9782 | 0.0029 |
| PLXNA1 | 1.311(1.0959-1.5684) | 2.9615 | 0.0031 |
| RXFP1 | 0.7026(0.5559-0.8879) | -2.9548 | 0.0031 |
| AMBN | 3.7376(1.5501-9.0118) | 2.9361 | 0.0033 |
| TRIM27 | 0.6889(0.5367-0.8841) | -2.9271 | 0.0034 |
| CRHR1 | 1.2106(1.0649-1.3761) | 2.9212 | 0.0035 |
| IL2 | 0.3174(0.1459-0.6902) | -2.8953 | 0.0038 |
| PTGFR | 1.3088(1.0854-1.5782) | 2.8177 | 0.0048 |
| IFNB1 | 0.4996(0.3081-0.8102) | -2.8134 | 0.0049 |
| SLC10A2 | 5.5774(1.6548-18.7985) | 2.7724 | 0.0056 |
| UBR1 | 1.4883(1.1204-1.9771) | 2.7446 | 0.0061 |
| PPP3CA | 1.3341(1.0816-1.6456) | 2.693 | 0.0071 |
| TNFSF11 | 1.2752(1.0656-1.5261) | 2.6534 | 0.008 |
| IGF2R | 1.2679(1.0636-1.5115) | 2.6477 | 0.0081 |
| TNFRSF13B | 0.5936(0.402-0.8764) | -2.6233 | 0.0087 |
| TNFRSF17 | 0.7583(0.6165-0.9328) | -2.6179 | 0.0088 |
| LCN9 | 2.4205(1.2477-4.6959) | 2.6144 | 0.0089 |
| C5AR1 | 1.2032(1.0465-1.3834) | 2.5983 | 0.0094 |
| CACYBP | 0.7138(0.5528-0.9218) | -2.5845 | 0.0098 |
| HGF | 1.2383(1.0487-1.4623) | 2.5207 | 0.0117 |
| CCR7 | 0.7968(0.6667-0.9522) | -2.4981 | 0.0125 |
| PLXNB2 | 1.2689(1.0525-1.5298) | 2.4963 | 0.0125 |
| LTA | 0.7358(0.5758-0.9403) | -2.4521 | 0.0142 |
| INS-IGF2 | 1.7096(1.1122-2.628) | 2.4447 | 0.0145 |
| RARG | 1.2539(1.0439-1.5061) | 2.4194 | 0.0155 |
| PSMC1 | 0.7091(0.5366-0.937) | -2.4179 | 0.0156 |
| PPARA | 1.305(1.0514-1.6198) | 2.4143 | 0.0158 |
| DKK1 | 1.2207(1.0375-1.4362) | 2.4042 | 0.0162 |
| ICOS | 0.7938(0.6571-0.959) | -2.3935 | 0.0167 |
| TRIM5 | 0.7496(0.5906-0.9515) | -2.3686 | 0.0179 |
| SH3BP2 | 1.2218(1.0334-1.4444) | 2.3448 | 0.019 |
| AKT1 | 0.7716(0.6204-0.9595) | -2.3317 | 0.0197 |
| IL26 | 0.3492(0.1437-0.8488) | -2.3217 | 0.0202 |
| MALT1 | 0.7821(0.6338-0.9651) | -2.2916 | 0.0219 |
| CETP | 0.7261(0.5519-0.9554) | -2.2854 | 0.0223 |
| LCN6 | 1.5094(1.0595-2.1504) | 2.28 | 0.0226 |
| AKT2 | 1.2275(1.0288-1.4644) | 2.2757 | 0.0229 |
| RLN3 | 1.3762(1.0445-1.8133) | 2.2691 | 0.0233 |
| RAF1 | 1.4399(1.0505-1.9736) | 2.2663 | 0.0234 |
| VAV2 | 1.2046(1.0254-1.4153) | 2.2646 | 0.0235 |
| LTBP3 | 1.2536(1.0296-1.5263) | 2.2505 | 0.0244 |
| LRSAM1 | 1.3493(1.0376-1.7546) | 2.2353 | 0.0254 |
| TGFBR2 | 1.2234(1.0244-1.461) | 2.2264 | 0.026 |
| GDF6 | 1.2068(1.0227-1.424) | 2.226 | 0.026 |
| UCN2 | 0.6768(0.4787-0.9569) | -2.2094 | 0.0271 |
| CMTM4 | 1.2299(1.022-1.4801) | 2.1903 | 0.0285 |
| NR1D1 | 1.2265(1.0213-1.473) | 2.1857 | 0.0288 |
| FGF23 | 1.5801(1.048-2.3822) | 2.184 | 0.029 |
| FABP9 | 2.8436(1.1019-7.3385) | 2.1606 | 0.0307 |
| RETN | 1.4582(1.0323-2.0598) | 2.1404 | 0.0323 |
| CLDN4 | 1.2011(1.0152-1.4211) | 2.1364 | 0.0327 |
| FCGR3B | 1.2072(1.0129-1.4388) | 2.1026 | 0.0355 |
| AP3B1 | 1.3937(1.0222-1.9003) | 2.0985 | 0.0359 |
| SEMA4F | 1.2742(1.0149-1.5996) | 2.0877 | 0.0368 |
| SEMA6B | 1.2365(1.0097-1.5142) | 2.0534 | 0.04 |
| IL24 | 0.7722(0.6022-0.9902) | -2.0378 | 0.0416 |
| IFNG | 0.7273(0.5349-0.9891) | -2.03 | 0.0424 |
| NFYC | 0.7566(0.5775-0.9911) | -2.0247 | 0.0429 |
| ELANE | 1.9838(1.0178-3.8668) | 2.0117 | 0.0443 |
| RXRA | 1.2167(1.0048-1.4733) | 2.0093 | 0.0445 |
| HSPA1L | 0.768(0.5933-0.9941) | -2.0051 | 0.045 |
| FGF22 | 1.3697(1.0069-1.8632) | 2.0037 | 0.0451 |
| NFKB1 | 1.2348(1.0046-1.5178) | 2.0034 | 0.0451 |
| IL31RA | 0.5984(0.3619-0.9895) | -2.001 | 0.0454 |
| TGFBR1 | 1.3113(1.0052-1.7106) | 1.9985 | 0.0457 |
| HSP90AB1 | 0.7758(0.6034-0.9975) | -1.9796 | 0.0477 |
| DEFB119 | 2.1781(1.0067-4.7124) | 1.977 | 0.048 |
| ZC3HAV1 | 0.7546(0.57-0.9991) | -1.9663 | 0.0493 |
| OPRD1 | 0.6938(0.4816-0.9995) | -1.9626 | 0.0497 |
| ELAVL1 | 0.705(0.4972-0.9998) | -1.9613 | 0.0498 |

| **The prognostic value of metabolic genes** | | | |
| --- | --- | --- | --- |
| **Gene** | **HR (95%CI)** | **z** | ***P* value** |
| CD38 | 0.794(0.7028-0.897) | -3.7058 | 2.00E-04 |
| GALNT10 | 1.3991(1.1522-1.6988) | 3.3906 | 7.00E-04 |
| TPMT | 0.7045(0.5749-0.8634) | -3.3752 | 7.00E-04 |
| PDP1 | 1.3924(1.1471-1.6902) | 3.3485 | 8.00E-04 |
| PLA2G2D | 0.7944(0.6921-0.9119) | -3.2702 | 0.0011 |
| PYGB | 1.3841(1.1284-1.6976) | 3.1199 | 0.0018 |
| PIGS | 1.2964(1.0948-1.5352) | 3.0101 | 0.0026 |
| DHRS9 | 1.2094(1.0665-1.3714) | 2.9639 | 0.003 |
| SIRT5 | 0.7139(0.5703-0.8936) | -2.9424 | 0.0033 |
| ALOX12 | 1.3609(1.1069-1.6733) | 2.923 | 0.0035 |
| H6PD | 1.2786(1.0824-1.5102) | 2.8921 | 0.0038 |
| EZH1 | 1.4492(1.1254-1.866) | 2.876 | 0.004 |
| GGCX | 1.5786(1.155-2.1574) | 2.8643 | 0.0042 |
| LPIN3 | 1.3199(1.0913-1.5964) | 2.8602 | 0.0042 |
| PRIM2 | 0.7175(0.5705-0.9024) | -2.8378 | 0.0045 |
| ITPKC | 1.2832(1.0793-1.5257) | 2.8242 | 0.0047 |
| ALDH5A1 | 0.7718(0.6445-0.9242) | -2.8169 | 0.0048 |
| GBGT1 | 1.2871(1.0727-1.5444) | 2.7149 | 0.0066 |
| ECI2 | 0.7716(0.6396-0.9309) | -2.7079 | 0.0068 |
| GCH1 | 0.7782(0.6459-0.9375) | -2.6385 | 0.0083 |
| KMT2B | 1.3068(1.0712-1.5941) | 2.6385 | 0.0083 |
| CALM1 | 0.738(0.5884-0.9256) | -2.6289 | 0.0086 |
| PDE7B | 1.2949(1.0678-1.5704) | 2.6264 | 0.0086 |
| CH25H | 1.2733(1.0632-1.5248) | 2.6262 | 0.0086 |
| PC | 1.2489(1.0554-1.4779) | 2.5876 | 0.0097 |
| PGM3 | 0.7372(0.5846-0.9296) | -2.5768 | 0.01 |
| CERK | 1.2665(1.0577-1.5165) | 2.57 | 0.0102 |
| CTPS2 | 0.7505(0.6028-0.9345) | -2.5658 | 0.0103 |
| NDUFV2 | 0.7673(0.6259-0.9408) | -2.547 | 0.0109 |
| HPGDS | 1.2906(1.0604-1.5708) | 2.5445 | 0.0109 |
| UST | 1.2233(1.0473-1.4289) | 2.5426 | 0.011 |
| ST6GALNAC6 | 1.273(1.0562-1.5343) | 2.5337 | 0.0113 |
| SIRT2 | 1.2893(1.0587-1.5701) | 2.5273 | 0.0115 |
| ALG8 | 0.7692(0.6273-0.9432) | -2.5217 | 0.0117 |
| GALNT15 | 1.2102(1.0433-1.4038) | 2.5191 | 0.0118 |
| PCYT1A | 1.3713(1.0698-1.7577) | 2.4926 | 0.0127 |
| OAZ3 | 0.6448(0.4564-0.911) | -2.4888 | 0.0128 |
| GGT7 | 1.242(1.0471-1.4732) | 2.4879 | 0.0129 |
| GPAT4 | 1.3745(1.0697-1.7661) | 2.4864 | 0.0129 |
| PLA2G12A | 0.7774(0.6371-0.9485) | -2.4807 | 0.0131 |
| TH | 1.2643(1.049-1.5237) | 2.4621 | 0.0138 |
| ADCY9 | 1.2348(1.0439-1.4605) | 2.462 | 0.0138 |
| PDIA4 | 0.7824(0.6434-0.9514) | -2.4598 | 0.0139 |
| SYNJ2 | 1.2858(1.0519-1.5717) | 2.4542 | 0.0141 |
| HS3ST3B1 | 1.2346(1.0418-1.463) | 2.4322 | 0.015 |
| PDE1C | 1.2856(1.0483-1.5766) | 2.4131 | 0.0158 |
| MGAT5 | 1.2434(1.0408-1.4853) | 2.401 | 0.0164 |
| LPCAT3 | 1.2716(1.0421-1.5517) | 2.3661 | 0.018 |
| ASL | 1.2522(1.0392-1.5089) | 2.3645 | 0.0181 |
| MGAM | 1.3067(1.0453-1.6335) | 2.349 | 0.0188 |
| BAAT | 0.539(0.3206-0.906) | -2.3323 | 0.0197 |
| POLR1A | 1.3535(1.0472-1.7493) | 2.3122 | 0.0208 |
| DNAJB11 | 0.7744(0.6233-0.9622) | -2.3074 | 0.021 |
| ACSM1 | 0.736(0.5657-0.9576) | -2.283 | 0.0224 |
| PRDM6 | 1.2309(1.0298-1.4712) | 2.2828 | 0.0224 |
| MVK | 1.2782(1.0343-1.5796) | 2.2723 | 0.0231 |
| AUH | 1.3032(1.0369-1.6379) | 2.2703 | 0.0232 |
| HS6ST3 | 1.2312(1.0285-1.4739) | 2.2664 | 0.0234 |
| PRIM1 | 0.7984(0.6567-0.9708) | -2.2574 | 0.024 |
| KYAT1 | 1.3066(1.0349-1.6495) | 2.2486 | 0.0245 |
| ALG12 | 1.3236(1.0365-1.6903) | 2.2475 | 0.0246 |
| B4GALT5 | 1.284(1.0323-1.5972) | 2.2454 | 0.0247 |
| ST6GALNAC4 | 1.226(1.0255-1.4657) | 2.2358 | 0.0254 |
| UGT2B4 | 1.8352(1.0771-3.1269) | 2.2333 | 0.0255 |
| P4HA3 | 1.2545(1.0269-1.5326) | 2.2201 | 0.0264 |
| PDE2A | 1.2125(1.022-1.4385) | 2.209 | 0.0272 |
| LTA4H | 1.2516(1.0242-1.5295) | 2.1938 | 0.0282 |
| NDST1 | 1.2087(1.0201-1.4321) | 2.1894 | 0.0286 |
| LIPT2 | 0.7848(0.6307-0.9767) | -2.171 | 0.0299 |
| UXS1 | 0.7273(0.5455-0.9697) | -2.1692 | 0.0301 |
| UGT1A6 | 0.5018(0.2688-0.9369) | -2.1647 | 0.0304 |
| ACACA | 1.2471(1.0211-1.5232) | 2.1645 | 0.0304 |
| AZIN2 | 1.2644(1.022-1.5643) | 2.1608 | 0.0307 |
| PAICS | 0.7857(0.6306-0.979) | -2.1495 | 0.0316 |
| RDH12 | 0.6782(0.476-0.9664) | -2.1492 | 0.0316 |
| GPAT3 | 1.2344(1.0187-1.4957) | 2.1487 | 0.0317 |
| POLR3H | 1.3344(1.0242-1.7387) | 2.1368 | 0.0326 |
| ACLY | 1.3102(1.0225-1.6787) | 2.136 | 0.0327 |
| ATP6V1B2 | 1.3056(1.0213-1.6689) | 2.1284 | 0.0333 |
| CA5A | 1.8216(1.0485-3.1646) | 2.1281 | 0.0333 |
| SUV39H2 | 0.7823(0.6238-0.9811) | -2.1252 | 0.0336 |
| MAN1A2 | 0.7477(0.5715-0.9781) | -2.1212 | 0.0339 |
| HMBS | 0.7874(0.6312-0.9823) | -2.1184 | 0.0341 |
| MTMR14 | 1.3779(1.0225-1.857) | 2.1059 | 0.0352 |
| HIBCH | 0.7673(0.5993-0.9824) | -2.1012 | 0.0356 |
| AGK | 0.7147(0.5208-0.9808) | -2.0802 | 0.0375 |
| ENPP7 | 0.5367(0.2977-0.9676) | -2.0695 | 0.0385 |
| TPH1 | 1.3802(1.0162-1.8745) | 2.063 | 0.0391 |
| MAN2A1 | 1.2327(1.0103-1.5042) | 2.0606 | 0.0393 |
| AMPD1 | 0.7087(0.51-0.9848) | -2.0513 | 0.0402 |
| POLD2 | 0.7996(0.6456-0.9902) | -2.0499 | 0.0404 |
| GLUD1 | 1.2557(1.0065-1.5666) | 2.0176 | 0.0436 |
| CPT1A | 1.2179(1.0043-1.477) | 2.0035 | 0.0451 |
| ETNPPL | 0.694(0.4847-0.9938) | -1.994 | 0.0462 |
| CHKA | 1.2164(1.0032-1.4749) | 1.9922 | 0.0464 |
| ZC3HAV1 | 0.7546(0.57-0.9991) | -1.9663 | 0.0493 |
| ALG11 | 1.3304(1.0007-1.7687) | 1.9651 | 0.0494 |
