# Supplementary material for: Tumor Immunometabolism Characterization in Ovarian Cancer With Prognostic and Therapeutic Implications
Source: Front Oncol. 2021 Mar 16;11:622752. doi: 10.3389/fonc.2021.622752 (PMC8008085; doi:10.3389/fonc.2021.622752)
Supplement: Supplementary file 12 [file Table_3.doc]

**Supplementary Table S3: Immunometabolism gene signature.**

| **Symbol** | **Class** |
| --- | --- |
| HLA-DOB | immune |
| CD40LG | immune |
| ANGPT4 | immune |
| CD3G | immune |
| AMBN | immune |
| PTH | immune |
| TRIM27 | immune |
| IL2 | immune |
| RXFP1 | immune |
| PLXNA1 | immune |
| CRHR1 | immune |
| IFNB1 | immune |
| SLC10A2 | immune |
| PPP3CA | immune |
| UBR1 | immune |
| PTGFR | immune |
| TNFRSF13B | immune |
| TNFSF11 | immune |
| LCN9 | immune |
| CCR7 | immune |
| IGF2R | immune |
| TNFRSF17 | immune |
| CACYBP | immune |
| INS-IGF2 | immune |
| HGF | immune |
| PSMC1 | immune |
| PLXNB2 | immune |
| RARG | immune |
| LTA | immune |
| TRIM5 | immune |
| ICOS | immune |
| PPARA | immune |
| DKK1 | immune |
| SH3BP2 | immune |
| CETP | immune |
| AKT1 | immune |
| MALT1 | immune |
| RAF1 | immune |
| AKT2 | immune |
| IL26 | immune |
| LCN6 | immune |
| VAV2 | immune |
| NR1D1 | immune |
| UCN2 | immune |
| RLN3 | immune |
| FGF23 | immune |
| FABP9 | immune |
| LRSAM1 | immune |
| LTBP3 | immune |
| NTF4 | immune |
| CLDN4 | immune |
| CMTM4 | immune |
| TGFBR2 | immune |
| IL24 | immune |
| SEMA4F | immune |
| FGF22 | immune |
| RETN | immune |
| AP3B1 | immune |
| IFNG | immune |
| RXRA | immune |
| HSPA1L | immune |
| NFYC | immune |
| DEFB119 | immune |
| CD38 | metabolic |
| PDP1 | metabolic |
| TPMT | metabolic |
| PLA2G2D | metabolic |
| GALNT10 | metabolic |
| PYGB | metabolic |
| PIGS | metabolic |
| SIRT5 | metabolic |
| ALOX12 | metabolic |
| GGCX | metabolic |
| H6PD | metabolic |
| DHRS9 | metabolic |
| EZH1 | metabolic |
| LPIN3 | metabolic |
| ALDH5A1 | metabolic |
| ITPKC | metabolic |
| PRIM2 | metabolic |
| ECI2 | metabolic |
| KMT2B | metabolic |
| GCH1 | metabolic |
| CTPS2 | metabolic |
| GBGT1 | metabolic |
| PGM3 | metabolic |
| CALM1 | metabolic |
| PDE7B | metabolic |
| HPGDS | metabolic |
| PC | metabolic |
| UST | metabolic |
| SIRT2 | metabolic |
| GGT7 | metabolic |
| CH25H | metabolic |
| CERK | metabolic |
| PDIA4 | metabolic |
| NDUFV2 | metabolic |
| GPAT4 | metabolic |
| PLA2G12A | metabolic |
| ALG8 | metabolic |
| PCYT1A | metabolic |
| ST6GALNAC6 | metabolic |
| TH | metabolic |
| OAZ3 | metabolic |
| ADCY9 | metabolic |
| SYNJ2 | metabolic |
| GALNT15 | metabolic |
| LPCAT3 | metabolic |
| BAAT | metabolic |
| HS3ST3B1 | metabolic |
| PDE1C | metabolic |
| MGAM | metabolic |
| MGAT5 | metabolic |
| AUH | metabolic |
| ASL | metabolic |
| MVK | metabolic |
| DNAJB11 | metabolic |
| KYAT1 | metabolic |
| ACSM1 | metabolic |
| POLR1A | metabolic |
| PRDM6 | metabolic |
| UGT2B4 | metabolic |
| UXS1 | metabolic |
| HS6ST3 | metabolic |
| ST6GALNAC4 | metabolic |
| ALG12 | metabolic |
| B4GALT5 | metabolic |
| TPH1 | metabolic |
| LTA4H | metabolic |
| MTMR14 | metabolic |
| UGT1A6 | metabolic |
| SUV39H2 | metabolic |
| LIPT2 | metabolic |
| POLR3H | metabolic |
| RDH12 | metabolic |
| ACACA | metabolic |
| NDST1 | metabolic |
| PDE2A | metabolic |
| CA5A | metabolic |
| AZIN2 | metabolic |
| MAN1A2 | metabolic |
| ATP6V1B2 | metabolic |
| GPAT3 | metabolic |
| PAICS | metabolic |
| ACLY | metabolic |
| HMBS | metabolic |
| HIBCH | metabolic |
| P4HA3 | metabolic |
| AMPD1 | metabolic |
| AGK | metabolic |
| MAN2A1 | metabolic |
| CHKA | metabolic |
| PTDSS1 | metabolic |
| HGSNAT | metabolic |
| ZC3HAV1 | metabolic |
| GLUD1 | metabolic |
| RCE1 | metabolic |
| ENPP7 | metabolic |
| ETNPPL | metabolic |
| CPT1A | metabolic |
| ALG11 | metabolic |
