# Supplementary material for: Tumor Immunometabolism Characterization in Ovarian Cancer With Prognostic and Therapeutic Implications
Source: Front Oncol. 2021 Mar 16;11:622752. doi: 10.3389/fonc.2021.622752 (PMC8008085; doi:10.3389/fonc.2021.622752)
Supplement: Supplementary file 13 [file Table_4.doc]

**Supplementary Table S4: Survival statistics, univariable and multivariable analysis with overall survival in TCGA cohort and independent validation cohort.**

|  | **C1 vs. C2** | **C1 vs. C3** | **C2 vs. C3** |
| --- | --- | --- | --- |
| TCGA | ***P* < 0.0001** | ***P* = 0.039** | ***P* = 0.0001** |
| Validation 1 | ***P* = 0.001** | *P* = 0.210 | ***P* = 0.004** |
| Validation 2 | ***P* = 0.011** | *P* = 0.983 | ***P* = 0.011** |

| **TCGA** | **Median OS** | **HR (95%CI)** | ***P* value** |
| --- | --- | --- | --- |
| C1 | 39 | 1.70(1.29-2.25) | **0.0002** |
| C2 | 65.5 | 0.45(0.32-0.62) | **<0.0001** |
| C3 | 43.9 | 1.22(0.94-1.58) | 0.1379 |

| **Validation 1** | **Median OS** | **HR (95%CI)** | ***P* value** |
| --- | --- | --- | --- |
| C1 | 27 | 1.35(1.07-1.70) | **0.0125** |
| C2 | 45 | 0.63(0.48-0.81) | **0.0004** |
| C3 | 37 | 1.13(0.91-1.39) | 0.2698 |

| **Validation 2** | **Median OS** | **HR (95%CI)** | ***P* value** |
| --- | --- | --- | --- |
| C1 | 76 | 1.26(0.69-2.30) | 0.456 |
| C2 | NA | 0.21(0.07-0.68) | **0.009** |
| C3 | 90 | 1.63(0.93-2.86) | 0.085 |

| **TCGA** | **Univariate analysis** | | **Multivariate analysis** | |
| --- | --- | --- | --- | --- |
|  | **HR (95%CI)** | ***P* value** | **HR (95%CI)** | ***P* value** |
| Subtype (C1 and C3 vs. C2) | 2.225(1.603-3.089) | **0** | 2.193(1.579-3.044) | **< 0.0001** |
| Stage (III and IV vs.  I and II) | 1.994(0.885-4.493) | **0.096** | 1.947(0.864-4.387) | 0.108 |
| Age (≥median vs.  <median) | 1.316 (1.015-1.707) | **0.0386** | 1.275(0.982-1.654) | **0.068** |

| **Validation 1** | **Univariate analysis** | | **Multivariate analysis** | |
| --- | --- | --- | --- | --- |
|  | **HR (95%CI)** | ***P* value** | **HR (95%CI)** | ***P* value** |
| Subtype (C1 and C3 vs. C2) | 1.600 (1.233-2.076) | **0.0004** | 1.598(1.231-2.073) | **0.0004** |
| Stage (III and IV vs.  I and II) | 2.478 (1.476-4.160) | **0.0006** | 2.474(1.473-4.153) | **0.0006** |

| **Validation 2** | **Univariate analysis** | | **Multivariate analysis** | |
| --- | --- | --- | --- | --- |
|  | **HR (95%CI)** | ***P* value** | **HR (95%CI)** | ***P* value** |
| Subtype (C1 and C3 vs. C2) | 4.742 (1.479-15.204) | **0.0088** | 3.129(0.953-10.273) | 0.06 |
| Stage (III and IV vs.  I and II) | 2.963 (1.659-5.292) | **0.0002** | 2.546(1.411-4.594) | **0.002** |
| Age (≥median vs.  <median) | 1.786 (1.054-3.027) | **0.031** | 1.58(0.927-2.691) | 0.093 |
