# Supplementary material for: Tumor Immunometabolism Characterization in Ovarian Cancer With Prognostic and Therapeutic Implications
Source: Front Oncol. 2021 Mar 16;11:622752. doi: 10.3389/fonc.2021.622752 (PMC8008085; doi:10.3389/fonc.2021.622752)
Supplement: Supplementary file 14 [file Table_5.doc]

**Supplementary Table S5: DEGs analysis of subtypes**

| **DEGs analysis of C1 with other subtypes** | | | | |
| --- | --- | --- | --- | --- |
| **Gene** | **Mean** | **Log_2_FC** | ***P* value** | **FDR** |
| CD93 | 1641.82 | 1.16 | 2.47E-58 | 4.83E-54 |
| ARHGAP31 | 869.23 | 1.28 | 6.49E-57 | 6.34E-53 |
| MRVI1 | 807.84 | 1.62 | 6.64E-54 | 4.32E-50 |
| ZEB2 | 1184.6 | 1.33 | 1.88E-53 | 9.17E-50 |
| STARD8 | 301.43 | 1.14 | 7.44E-53 | 2.91E-49 |
| TGFBI | 10241.4 | 1.59 | 2.43E-51 | 7.91E-48 |
| ADGRA2 | 1754.15 | 1.57 | 1.05E-49 | 2.92E-46 |
| ZNF469 | 1526.78 | 1.88 | 2.17E-48 | 5.30E-45 |
| ANTXR2 | 853.92 | 1.37 | 8.50E-47 | 1.84E-43 |
| FBN1 | 3275.26 | 1.88 | 1.88E-45 | 3.67E-42 |
| FLI1 | 502.49 | 1.11 | 3.97E-45 | 6.57E-42 |
| ITGA5 | 3691.97 | 1.29 | 1.01E-44 | 1.42E-41 |
| ZCCHC24 | 1293.87 | 1.33 | 1.35E-44 | 1.76E-41 |
| FN1 | 91847.54 | 2.07 | 3.09E-44 | 3.77E-41 |
| AEBP1 | 20880.47 | 1.81 | 5.89E-44 | 6.77E-41 |
| PRDM8 | 65.35 | 1.46 | 1.13E-42 | 1.23E-39 |
| ARHGEF6 | 564.12 | 1.12 | 4.18E-42 | 4.30E-39 |
| ITGA11 | 2260.55 | 2.07 | 8.52E-42 | 8.32E-39 |
| LRP1 | 17366.13 | 1.2 | 1.03E-41 | 9.60E-39 |
| THBD | 694.48 | 1.45 | 1.66E-41 | 1.47E-38 |
| GIMAP8 | 260.66 | 1.21 | 2.43E-41 | 2.06E-38 |
| COL5A2 | 7781.85 | 1.98 | 7.60E-41 | 6.06E-38 |
| COL5A1 | 19936.34 | 2.02 | 7.76E-41 | 6.06E-38 |
| F13A1 | 1793.16 | 1.72 | 1.05E-40 | 7.85E-38 |
| FZD1 | 2376.72 | 1.19 | 1.43E-40 | 1.04E-37 |
| IFFO1 | 360.7 | 1.19 | 2.00E-40 | 1.40E-37 |
| MYH11 | 1254.83 | 2.17 | 3.18E-40 | 2.14E-37 |
| GALNT15 | 149.57 | 1.79 | 3.92E-40 | 2.55E-37 |
| EMILIN1 | 4090.39 | 1.63 | 4.43E-40 | 2.79E-37 |
| PDGFRB | 3976.87 | 1.43 | 9.92E-40 | 5.87E-37 |
| PODN | 1187.38 | 1.89 | 1.63E-39 | 9.37E-37 |
| HIC1 | 714.3 | 1.38 | 6.02E-39 | 3.36E-36 |
| RASGRF2 | 362.98 | 1.31 | 9.10E-39 | 4.93E-36 |
| COL6A3 | 21920.76 | 1.69 | 1.37E-38 | 7.22E-36 |
| ANTXR1 | 8702.01 | 1.36 | 2.28E-38 | 1.17E-35 |
| RAB31 | 3312.21 | 1.12 | 4.30E-38 | 2.15E-35 |
| IL16 | 546.48 | 1.23 | 4.83E-38 | 2.30E-35 |
| GNG2 | 472.03 | 1.14 | 5.89E-38 | 2.74E-35 |
| COL16A1 | 2300.45 | 1.45 | 6.82E-38 | 3.10E-35 |
| CRISPLD2 | 2251.64 | 1.79 | 1.87E-37 | 8.28E-35 |
| WDFY4 | 384.29 | 1.44 | 3.00E-37 | 1.30E-34 |
| DAB2 | 2654.04 | 1.05 | 3.58E-37 | 1.52E-34 |
| WISP1 | 548.18 | 1.67 | 4.14E-37 | 1.72E-34 |
| ADAMTS2 | 1716.26 | 1.8 | 5.82E-37 | 2.37E-34 |
| FRMD6 | 912.33 | 1.23 | 5.94E-37 | 2.37E-34 |
| LMOD1 | 642.29 | 1.53 | 7.79E-37 | 3.04E-34 |
| GPR68 | 163.19 | 1.5 | 8.66E-37 | 3.32E-34 |
| HTR7 | 21.49 | 1.63 | 2.52E-36 | 9.27E-34 |
| COLEC12 | 1132 | 1.56 | 3.46E-36 | 1.25E-33 |
| NLRP3 | 170.77 | 1.27 | 3.80E-36 | 1.35E-33 |
| LOXL2 | 2509.96 | 1.24 | 7.47E-36 | 2.60E-33 |
| DOCK10 | 493.18 | 1.12 | 1.16E-35 | 3.98E-33 |
| SYNE1 | 1008.21 | 1.05 | 1.31E-35 | 4.33E-33 |
| CHSY3 | 137.79 | 1.59 | 1.33E-35 | 4.33E-33 |
| SH3PXD2A | 4171.48 | 1.08 | 3.05E-35 | 9.61E-33 |
| COL8A1 | 3603.14 | 1.92 | 5.26E-35 | 1.62E-32 |
| CSF2RB | 384.05 | 1.48 | 5.30E-35 | 1.62E-32 |
| MMP2 | 16409.41 | 1.6 | 9.48E-35 | 2.85E-32 |
| PAPSS2 | 768.98 | 1.16 | 2.03E-34 | 5.91E-32 |
| ADGRE2 | 233.26 | 1.38 | 2.21E-34 | 6.35E-32 |
| EHD2 | 5000.85 | 1.16 | 2.54E-34 | 7.20E-32 |
| ANGPTL2 | 1887.63 | 1.25 | 2.74E-34 | 7.65E-32 |
| KIF26B | 1504.39 | 1.79 | 2.79E-34 | 7.68E-32 |
| SLCO2B1 | 2373.36 | 1.41 | 3.96E-34 | 1.06E-31 |
| THBS2 | 7369.97 | 2 | 4.54E-34 | 1.20E-31 |
| CNRIP1 | 363.15 | 1.17 | 4.66E-34 | 1.21E-31 |
| VCAN | 6224.22 | 1.83 | 5.91E-34 | 1.52E-31 |
| SFRP4 | 3772.67 | 2.12 | 6.42E-34 | 1.63E-31 |
| PIK3R5 | 391.96 | 1.18 | 7.87E-34 | 1.97E-31 |
| STAB1 | 3731.79 | 1.27 | 1.09E-33 | 2.67E-31 |
| MMP14 | 13475.36 | 1.26 | 1.09E-33 | 2.67E-31 |
| ITGA4 | 362.44 | 1.3 | 1.21E-33 | 2.90E-31 |
| GAB3 | 165.91 | 1.16 | 2.02E-33 | 4.76E-31 |
| GFPT2 | 595.6 | 1.44 | 2.85E-33 | 6.54E-31 |
| CSF1R | 4058.39 | 1.4 | 4.66E-33 | 1.05E-30 |
| LHFPL6 | 902.37 | 1.19 | 5.20E-33 | 1.15E-30 |
| DOCK2 | 618.99 | 1.32 | 9.88E-33 | 2.17E-30 |
| MYO1F | 992.45 | 1.11 | 1.53E-32 | 3.33E-30 |
| BGN | 27686.9 | 1.35 | 1.76E-32 | 3.78E-30 |
| COL1A2 | 124520 | 1.54 | 1.88E-32 | 3.99E-30 |
| NID1 | 2569.94 | 1.19 | 2.40E-32 | 4.98E-30 |
| ECM2 | 420.93 | 1.46 | 2.90E-32 | 5.93E-30 |
| COL1A1 | 297196 | 1.73 | 2.91E-32 | 5.93E-30 |
| OLFML2B | 1857.35 | 1.57 | 3.04E-32 | 6.13E-30 |
| GALNT5 | 200.8 | 1.73 | 4.69E-32 | 9.26E-30 |
| AOC3 | 468.88 | 1.59 | 1.25E-31 | 2.43E-29 |
| FNDC1 | 2421.18 | 1.77 | 1.97E-31 | 3.81E-29 |
| HEG1 | 3981.28 | 1.23 | 2.27E-31 | 4.34E-29 |
| COL6A2 | 30375.59 | 1.39 | 2.47E-31 | 4.68E-29 |
| MRC1 | 463.42 | 1.5 | 2.69E-31 | 5.06E-29 |
| ZNF366 | 42.44 | 1.02 | 5.02E-31 | 9.34E-29 |
| VGLL3 | 422.58 | 1.77 | 6.88E-31 | 1.27E-28 |
| CDH11 | 4004.35 | 1.5 | 8.18E-31 | 1.48E-28 |
| ZEB1 | 680.7 | 1.15 | 8.57E-31 | 1.54E-28 |
| P4HA3 | 131.93 | 1.26 | 1.02E-30 | 1.80E-28 |
| ABCA6 | 105.44 | 1.58 | 1.02E-30 | 1.80E-28 |
| COL3A1 | 94374.8 | 1.79 | 1.04E-30 | 1.81E-28 |
| ADAM12 | 1710.93 | 1.87 | 1.60E-30 | 2.76E-28 |
| GLIPR1 | 832.08 | 1.03 | 1.64E-30 | 2.81E-28 |
| CHRD | 232.68 | 1.36 | 1.80E-30 | 3.06E-28 |
| TIMP3 | 913.47 | 1.77 | 1.89E-30 | 3.18E-28 |

| **DEGs analysis of C2 with other subtypes** | | | | |
| --- | --- | --- | --- | --- |
| **Gene** | **Mean** | **Log_2_FC** | ***P* value** | **FDR** |
| PYY | 116.31 | 4.04 | 2.03E-56 | 1.32E-52 |
| ATP5ME | 6500.17 | 1.25 | 1.85E-28 | 2.12E-25 |
| NME1 | 5404.63 | 1.01 | 9.59E-27 | 6.46E-24 |
| COX7A2 | 10174.28 | 1 | 5.44E-26 | 3.02E-23 |
| PSMB9 | 3987.03 | 1.32 | 6.07E-24 | 2.16E-21 |
| HLA-DOB | 230.14 | 1.37 | 1.62E-23 | 4.66E-21 |
| CD52 | 1000.48 | 1.38 | 2.16E-23 | 5.95E-21 |
| CXCL11 | 1023.98 | 1.82 | 2.25E-23 | 6.02E-21 |
| CXCL10 | 4492.1 | 1.7 | 3.92E-23 | 9.81E-21 |
| APOC1 | 2431.21 | 1.23 | 2.97E-21 | 4.90E-19 |
| DIO1 | 89.89 | 1.94 | 1.35E-20 | 1.78E-18 |
| C4orf51 | 8.48 | 1.58 | 2.47E-18 | 1.90E-16 |
| CCL8 | 316.8 | 1.35 | 6.68E-18 | 4.57E-16 |
| HLA-G | 149.98 | 1.5 | 6.38E-17 | 3.47E-15 |
| ISG15 | 11204.21 | 1.27 | 1.05E-16 | 5.50E-15 |
| CD38 | 410.47 | 1.3 | 1.06E-16 | 5.50E-15 |
| CENPW | 1614.14 | 1.24 | 1.07E-16 | 5.55E-15 |
| HLA-F | 2268.6 | 1 | 1.13E-15 | 4.71E-14 |
| APOBEC3A | 102.44 | 1.29 | 1.44E-15 | 5.81E-14 |
| IFI27 | 42265.42 | 1.25 | 3.21E-15 | 1.20E-13 |
| CD3D | 144.63 | 1.38 | 4.41E-15 | 1.59E-13 |
| UBD | 269.19 | 1.72 | 4.78E-15 | 1.71E-13 |
| CD48 | 697.09 | 1.09 | 5.57E-15 | 1.96E-13 |
| CCL5 | 1779.59 | 1.23 | 8.47E-15 | 2.83E-13 |
| GZMB | 265.26 | 1.41 | 2.70E-14 | 8.05E-13 |
| CR1L | 36.21 | 1.33 | 3.19E-14 | 9.38E-13 |
| ANXA10 | 9.57 | 1.63 | 1.16E-13 | 3.07E-12 |
| IFI6 | 65287.39 | 1.22 | 1.98E-13 | 5.05E-12 |
| PMCH | 2.5 | 1.5 | 2.05E-13 | 5.18E-12 |
| SELL | 430.51 | 1 | 1.28E-12 | 2.74E-11 |
| SIT1 | 132.85 | 1.09 | 1.38E-12 | 2.92E-11 |
| CXCL13 | 408.88 | 1.62 | 2.27E-12 | 4.58E-11 |
| HLA-DRB1 | 25742.62 | 1 | 2.40E-12 | 4.80E-11 |
| IFNB1 | 4.75 | 1.48 | 2.68E-12 | 5.32E-11 |
| C4BPA | 84.89 | 1.83 | 3.28E-12 | 6.41E-11 |
| XCL2 | 56.8 | 1.2 | 4.72E-12 | 8.99E-11 |
| GBP4 | 3298.69 | 1.04 | 6.01E-12 | 1.12E-10 |
| RLN1 | 27.73 | 1.12 | 7.28E-12 | 1.34E-10 |
| NKG7 | 551.42 | 1.13 | 1.02E-11 | 1.81E-10 |
| DEFB123 | 5.85 | 2.53 | 1.22E-11 | 2.12E-10 |
| IFNG | 14.17 | 1.45 | 1.68E-11 | 2.82E-10 |
| GZMM | 42 | 1.03 | 2.41E-11 | 3.91E-10 |
| CAGE1 | 17.09 | 1.3 | 2.67E-11 | 4.28E-10 |
| SPINK2 | 22.49 | 1.18 | 3.16E-11 | 4.97E-10 |
| GZMA | 219.07 | 1.09 | 3.73E-11 | 5.79E-10 |
| CLEC4E | 154.19 | 1.14 | 5.08E-11 | 7.70E-10 |
| GPR171 | 58.21 | 1.12 | 6.17E-11 | 9.15E-10 |
| PPP1R14D | 51.22 | 1.24 | 6.23E-11 | 9.21E-10 |
| ANKRD22 | 167.69 | 1 | 7.33E-11 | 1.07E-09 |
| STAP1 | 23.41 | 1.04 | 1.20E-10 | 1.68E-09 |
| SH2D1A | 58.69 | 1.14 | 1.27E-10 | 1.76E-09 |
| CD2 | 427.47 | 1.1 | 1.76E-10 | 2.37E-09 |
| SLAMF7 | 568.28 | 1.19 | 2.79E-10 | 3.59E-09 |
| TNFRSF17 | 38.32 | 1.46 | 3.03E-10 | 3.88E-09 |
| IL2 | 1.19 | 1.53 | 3.71E-10 | 4.67E-09 |
| CSAG3 | 36.28 | 1.76 | 3.75E-10 | 4.70E-09 |
| GTSF1L | 2.43 | 1.67 | 3.85E-10 | 4.81E-09 |
| TRAT1 | 41.65 | 1.17 | 4.95E-10 | 6.04E-09 |
| HLA-DRB5 | 7538.6 | 1.2 | 6.59E-10 | 7.85E-09 |
| CXCR3 | 201.07 | 1 | 8.61E-10 | 9.96E-09 |
| PYHIN1 | 63.2 | 1.06 | 8.82E-10 | 1.02E-08 |
| CD3E | 552.97 | 1.03 | 9.96E-10 | 1.14E-08 |
| CLPSL2 | 16.78 | 1.32 | 1.08E-09 | 1.23E-08 |
| NCR3 | 25.66 | 1.03 | 1.09E-09 | 1.24E-08 |
| ICOS | 57.99 | 1.11 | 1.25E-09 | 1.40E-08 |
| HLA-DQA1 | 7664.67 | 1.02 | 1.26E-09 | 1.41E-08 |
| SIRPG | 93.45 | 1.06 | 1.62E-09 | 1.77E-08 |
| PLA2G2D | 117.23 | 1.47 | 1.69E-09 | 1.85E-08 |
| AIM2 | 97.86 | 1.1 | 1.72E-09 | 1.87E-08 |
| CCL13 | 61.54 | 1.38 | 2.14E-09 | 2.28E-08 |
| CCL7 | 61.69 | 1.27 | 2.35E-09 | 2.47E-08 |
| CXCL9 | 2286.8 | 1.33 | 2.98E-09 | 3.07E-08 |
| MZB1 | 507.91 | 1.36 | 4.17E-09 | 4.17E-08 |
| PAGE4 | 11.54 | 2.43 | 5.14E-09 | 5.06E-08 |
| LIPM | 7.81 | 1.03 | 5.49E-09 | 5.36E-08 |
| JSRP1 | 129.59 | 1.08 | 6.00E-09 | 5.82E-08 |
| DAPL1 | 5907.69 | 1.25 | 9.44E-09 | 8.78E-08 |
| GZMH | 91.89 | 1.02 | 1.70E-08 | 1.50E-07 |
| KHDC1L | 8.62 | 1.53 | 3.37E-08 | 2.80E-07 |
| CARD17 | 8.59 | 1.07 | 4.65E-08 | 3.74E-07 |
| JCHAIN | 4168.55 | 1.39 | 4.92E-08 | 3.93E-07 |
| CD79A | 388.27 | 1.25 | 5.23E-08 | 4.15E-07 |
| MT1M | 313.92 | 1.16 | 5.24E-08 | 4.16E-07 |
| MT1A | 162.35 | 1.13 | 6.85E-08 | 5.29E-07 |
| ADAMDEC1 | 379.5 | 1.17 | 8.24E-08 | 6.28E-07 |
| S100P | 80.17 | 1.12 | 8.26E-08 | 6.30E-07 |
| CCL17 | 23.46 | 1.06 | 1.23E-07 | 9.04E-07 |
| KIR2DL4 | 26.29 | 1 | 2.33E-07 | 1.62E-06 |
| STRA8 | 54.26 | 1.23 | 2.44E-07 | 1.69E-06 |
| SPINK8 | 2.58 | 1.55 | 2.53E-07 | 1.75E-06 |
| KLRC1 | 24.66 | 1.08 | 4.41E-07 | 2.91E-06 |
| HIST1H1A | 13.83 | 1.14 | 7.21E-07 | 4.53E-06 |
| TAC1 | 17.29 | 1.79 | 1.16E-06 | 6.94E-06 |
| SST | 8393.42 | 1.43 | 1.30E-06 | 7.71E-06 |
| CSAG2 | 16.73 | 1.44 | 1.41E-06 | 8.26E-06 |
| TEX48 | 1.98 | 1.29 | 1.46E-06 | 8.55E-06 |
| DEFB4A | 13.83 | 1.58 | 1.47E-06 | 8.58E-06 |
| SOHLH2 | 23.05 | 1.04 | 1.83E-06 | 1.05E-05 |
| UTS2 | 11.51 | 1.22 | 1.98E-06 | 1.12E-05 |
| PCK1 | 1766.49 | 1.18 | 2.04E-06 | 1.15E-05 |
| HMHB1 | 0.89 | 1.43 | 3.00E-06 | 1.63E-05 |

| **DEGs analysis of C3 with other subtypes** | | | | |
| --- | --- | --- | --- | --- |
| **Gene** | **Mean** | **Log_2_FC** | ***P* value** | **FDR** |
| FGF17 | 550.26 | 4.66 | 1.97E-73 | 3.49E-70 |
| CHRNA4 | 65.75 | 3.36 | 1.97E-36 | 1.37E-34 |
| SCG3 | 48.57 | 2.56 | 1.02E-30 | 5.46E-29 |
| CAMKV | 28.88 | 2.56 | 2.05E-28 | 9.64E-27 |
| HPSE2 | 114.05 | 3.29 | 5.48E-28 | 2.52E-26 |
| CELF3 | 20.66 | 2.53 | 3.77E-27 | 1.65E-25 |
| HNF1A | 12.12 | 2.52 | 4.31E-27 | 1.88E-25 |
| CTXND1 | 102.97 | 2.41 | 6.51E-27 | 2.83E-25 |
| MYH6 | 102.11 | 2.96 | 8.85E-27 | 3.81E-25 |
| SEC14L5 | 64.95 | 1.7 | 1.51E-26 | 6.42E-25 |
| DISP3 | 129.61 | 2.15 | 2.34E-26 | 9.82E-25 |
| RCOR2 | 1305.71 | 1.43 | 6.06E-26 | 2.50E-24 |
| BSN | 469.95 | 1.3 | 6.39E-25 | 2.46E-23 |
| SPINK1 | 44.58 | 2.43 | 1.25E-24 | 4.69E-23 |
| KCNC1 | 25.34 | 1.84 | 4.12E-24 | 1.51E-22 |
| DPYSL5 | 308.72 | 2.97 | 9.60E-24 | 3.43E-22 |
| TMEM145 | 45.71 | 1.64 | 3.78E-23 | 1.32E-21 |
| ELAVL3 | 52.12 | 2.33 | 1.50E-22 | 5.07E-21 |
| VIL1 | 36.79 | 1.96 | 2.79E-22 | 9.26E-21 |
| GPR17 | 73.75 | 1.75 | 7.71E-22 | 2.50E-20 |
| GDF10 | 112.96 | 2.42 | 1.77E-21 | 5.63E-20 |
| CUX2 | 117.9 | 2.37 | 1.99E-21 | 6.33E-20 |
| TDRD12 | 102.43 | 1.97 | 4.01E-21 | 1.25E-19 |
| CRYGB | 33.17 | 2.86 | 5.42E-21 | 1.68E-19 |
| ITIH2 | 52.33 | 1.37 | 1.09E-20 | 3.29E-19 |
| RSPH10B | 15.56 | 1.33 | 1.58E-20 | 4.76E-19 |
| SLC39A5 | 38.24 | 1.54 | 2.08E-20 | 6.16E-19 |
| ANKRD33 | 37.89 | 2.33 | 2.77E-20 | 8.16E-19 |
| CA14 | 181.48 | 1.1 | 3.35E-20 | 9.81E-19 |
| PRLHR | 3.51 | 3.01 | 4.01E-20 | 1.17E-18 |
| SP5 | 527.52 | 1.71 | 4.86E-20 | 1.41E-18 |
| PHF21B | 53.85 | 2.16 | 6.75E-20 | 1.94E-18 |
| BTBD17 | 31.84 | 3.08 | 8.53E-20 | 2.42E-18 |
| LRP4 | 993.58 | 1.67 | 9.29E-20 | 2.64E-18 |
| CARMIL3 | 125.54 | 1.14 | 2.43E-18 | 6.50E-17 |
| KIRREL3 | 123.26 | 1.61 | 2.69E-18 | 7.20E-17 |
| TMEM132B | 417.65 | 1.6 | 3.73E-18 | 9.90E-17 |
| KIRREL2 | 163.37 | 1.86 | 4.01E-18 | 1.06E-16 |
| GNG3 | 29.66 | 1.03 | 5.37E-18 | 1.40E-16 |
| NPHS1 | 85.93 | 2.02 | 5.58E-18 | 1.45E-16 |
| PRKAG3 | 11.01 | 1.83 | 9.09E-18 | 2.33E-16 |
| SEP14 | 8.54 | 2.78 | 1.03E-17 | 2.63E-16 |
| GJC2 | 387.4 | 1.31 | 1.12E-17 | 2.83E-16 |
| HHATL | 17.07 | 2.07 | 1.15E-17 | 2.90E-16 |
| ASTL | 67.16 | 1.23 | 1.91E-17 | 4.76E-16 |
| CHRND | 19.09 | 2.04 | 2.24E-17 | 5.55E-16 |
| TMEM221 | 468.94 | 1.32 | 5.75E-17 | 1.38E-15 |
| SHH | 74.26 | 2.23 | 6.51E-17 | 1.56E-15 |
| COL9A3 | 833.47 | 1.83 | 6.97E-17 | 1.66E-15 |
| CYP2W1 | 170.06 | 1.92 | 8.04E-17 | 1.90E-15 |
| UMODL1 | 89.55 | 1.74 | 8.63E-17 | 2.04E-15 |
| ANKDD1B | 28.26 | 1.2 | 1.09E-16 | 2.54E-15 |
| CACNA2D2 | 921.44 | 1.3 | 2.20E-16 | 5.04E-15 |
| PDZD3 | 20.34 | 1.24 | 2.75E-16 | 6.28E-15 |
| BRSK2 | 169.39 | 1.54 | 3.35E-16 | 7.61E-15 |
| DNAH6 | 235.39 | 1.12 | 8.72E-16 | 1.92E-14 |
| SLC26A7 | 345.17 | 1.56 | 9.20E-16 | 2.02E-14 |
| FBN3 | 1916.37 | 1.52 | 9.31E-16 | 2.05E-14 |
| CLDN19 | 204.4 | 2.35 | 9.37E-16 | 2.05E-14 |
| ATP4B | 10.24 | 1.63 | 9.59E-16 | 2.10E-14 |
| IHH | 31.91 | 2.2 | 1.35E-15 | 2.92E-14 |
| COL20A1 | 13.61 | 1.5 | 1.57E-15 | 3.38E-14 |
| CPA1 | 63.99 | 1.79 | 1.70E-15 | 3.65E-14 |
| CFAP61 | 104.64 | 1.22 | 1.83E-15 | 3.93E-14 |
| LKAAEAR1 | 85.2 | 1.31 | 1.99E-15 | 4.25E-14 |
| CYYR1 | 1658.99 | 1.3 | 2.98E-15 | 6.27E-14 |
| GAS2L2 | 74.78 | 1.64 | 3.71E-15 | 7.77E-14 |
| CROCC2 | 105.12 | 1.86 | 4.17E-15 | 8.69E-14 |
| FGF19 | 143.98 | 2.53 | 5.03E-15 | 1.04E-13 |
| MASP1 | 66.58 | 1.29 | 7.09E-15 | 1.45E-13 |
| TMEM151B | 27.38 | 1.45 | 8.96E-15 | 1.81E-13 |
| ARID3C | 48.7 | 1.43 | 1.12E-14 | 2.24E-13 |
| ZNF423 | 1528.3 | 1.18 | 1.22E-14 | 2.45E-13 |
| TNNT2 | 515.47 | 1.51 | 1.62E-14 | 3.22E-13 |
| CRYGC | 37.53 | 2.28 | 1.68E-14 | 3.33E-13 |
| SPEF1 | 355.99 | 1.15 | 2.94E-14 | 5.73E-13 |
| FLRT1 | 78.36 | 1.39 | 3.01E-14 | 5.86E-13 |
| ACSL6 | 39.59 | 1.28 | 3.39E-14 | 6.55E-13 |
| CCDC146 | 3742.78 | 1.28 | 3.48E-14 | 6.71E-13 |
| TMEM72 | 40.01 | 1.81 | 4.21E-14 | 8.06E-13 |
| PTF1A | 13.32 | 2.81 | 5.43E-14 | 1.03E-12 |
| CCDC173 | 131.46 | 1.07 | 6.18E-14 | 1.17E-12 |
| HYDIN | 327.16 | 1.04 | 6.90E-14 | 1.30E-12 |
| CLVS1 | 107.17 | 1.23 | 7.56E-14 | 1.42E-12 |
| MSI1 | 1971.28 | 1.07 | 7.99E-14 | 1.49E-12 |
| DAW1 | 85.92 | 1.62 | 1.07E-13 | 1.97E-12 |
| SLC13A4 | 235.11 | 1.03 | 1.27E-13 | 2.33E-12 |
| SLC16A12 | 42.07 | 1.73 | 1.44E-13 | 2.63E-12 |
| HELT | 4.62 | 2.49 | 1.50E-13 | 2.75E-12 |
| CASQ1 | 75.45 | 1.16 | 1.52E-13 | 2.79E-12 |
| TCTE1 | 51.89 | 1.54 | 1.55E-13 | 2.83E-12 |
| COL2A1 | 649.19 | 1.95 | 1.77E-13 | 3.23E-12 |
| CTNNA2 | 488.7 | 1.27 | 1.85E-13 | 3.36E-12 |
| SOX3 | 51.49 | 2.03 | 2.00E-13 | 3.61E-12 |
| FREM2 | 162.25 | 1.67 | 2.29E-13 | 4.13E-12 |
| DNAH11 | 157.08 | 1.09 | 2.35E-13 | 4.23E-12 |
| SPTB | 252.22 | 1.07 | 2.73E-13 | 4.88E-12 |
| TUBB2B | 893.19 | 1.89 | 2.81E-13 | 5.02E-12 |
| HGFAC | 139.71 | 1.1 | 3.12E-13 | 5.53E-12 |
| CNNM1 | 72.66 | 1.43 | 3.80E-13 | 6.70E-12 |
| GCGR | 13.39 | 1.72 | 4.71E-13 | 8.25E-12 |
