# Supplementary material for: Tumor Immunometabolism Characterization in Ovarian Cancer With Prognostic and Therapeutic Implications
Source: Front Oncol. 2021 Mar 16;11:622752. doi: 10.3389/fonc.2021.622752 (PMC8008085; doi:10.3389/fonc.2021.622752)
Supplement: Supplementary file 15 [file Table_6.doc]

**Supplementary Table S6: Significantly enriched gene sets for each subtype.**

| **Mean GSVA scores of upregulated HALLMARK gene sets** | | | |
| --- | --- | --- | --- |
|  | **C1** | **C2** | **C3** |
| HALLMARK_EPITHELIAL_MESENCHYMAL_TRANSITION | 0.2949 | -0.0411 | -0.1939 |
| HALLMARK_TNFA_SIGNALING_VIA_NFKB | 0.2307 | 0.0475 | -0.2126 |
| HALLMARK_INFLAMMATORY_RESPONSE | 0.2583 | 0.1371 | -0.2549 |
| HALLMARK_ALLOGRAFT_REJECTION | 0.196 | 0.235 | -0.2895 |
| HALLMARK_IL6_JAK_STAT3_SIGNALING | 0.2373 | 0.1291 | -0.2544 |
| HALLMARK_UV_RESPONSE_DN | 0.197 | -0.1552 | -0.0736 |
| HALLMARK_KRAS_SIGNALING_UP | 0.1956 | 0.067 | -0.1723 |
| HALLMARK_IL2_STAT5_SIGNALING | 0.1894 | 0.0519 | -0.1922 |
| HALLMARK_APICAL_JUNCTION | 0.1992 | -0.0995 | -0.0901 |
| HALLMARK_COMPLEMENT | 0.1702 | 0.1082 | -0.1933 |
| HALLMARK_INTERFERON_GAMMA_RESPONSE | 0.1206 | 0.239 | -0.2476 |
| HALLMARK_INTERFERON_ALPHA_RESPONSE | 0.0189 | 0.2573 | -0.188 |
| HALLMARK_OXIDATIVE_PHOSPHORYLATION | -0.1973 | 0.1515 | -0.0237 |
| HALLMARK_MYC_TARGETS_V1 | -0.2158 | 0.1091 | 0.0327 |
| HALLMARK_E2F_TARGETS | -0.1768 | 0.1081 | 0.0238 |
| HALLMARK_MTORC1_SIGNALING | -0.0355 | 0.0631 | -0.0721 |
| HALLMARK_DNA_REPAIR | -0.157 | 0.063 | 0.0005 |
| HALLMARK_PANCREAS_BETA_CELLS | -0.0001 | 0.1496 | 0.0398 |
| HALLMARK_KRAS_SIGNALING_DN | 0.0279 | 0.0498 | 0.0382 |
| HALLMARK_MYC_TARGETS_V2 | -0.1853 | -0.0049 | 0.0705 |
| HALLMARK_HEDGEHOG_SIGNALING | 0.1751 | -0.1948 | 0.0069 |
| HALLMARK_SPERMATOGENESIS | -0.0946 | 0.0749 | 0.047 |
| HALLMARK_WNT_BETA_CATENIN_SIGNALING | 0.087 | -0.1832 | 0.0367 |
| HALLMARK_NOTCH_SIGNALING | 0.0926 | -0.1499 | -0.0017 |
| HALLMARK_G2M_CHECKPOINT | -0.1013 | 0.0246 | 0.0146 |

| **Mean GSVA scores of downregulation of HALLMARK gene sets** | | | |
| --- | --- | --- | --- |
|  | **C1** | **C2** | **C3** |
| HALLMARK_MYC_TARGETS_V1 | -0.2158 | 0.1091 | 0.0327 |
| HALLMARK_E2F_TARGETS | -0.1768 | 0.1081 | 0.0238 |
| HALLMARK_OXIDATIVE_PHOSPHORYLATION | -0.1973 | 0.1515 | -0.0237 |
| HALLMARK_PANCREAS_BETA_CELLS | -0.0001 | 0.1496 | 0.0398 |
| HALLMARK_SPERMATOGENESIS | -0.0946 | 0.0749 | 0.047 |
| HALLMARK_MYC_TARGETS_V2 | -0.1853 | -0.0049 | 0.0705 |
| HALLMARK_DNA_REPAIR | -0.157 | 0.063 | 0.0005 |
| HALLMARK_G2M_CHECKPOINT | -0.1013 | 0.0246 | 0.0146 |
| HALLMARK_KRAS_SIGNALING_DN | 0.0279 | 0.0498 | 0.0382 |
| HALLMARK_PEROXISOME | -0.0394 | 0.0412 | -0.0386 |
| HALLMARK_HEDGEHOG_SIGNALING | 0.1751 | -0.1948 | 0.0069 |
| HALLMARK_EPITHELIAL_MESENCHYMAL_TRANSITION | 0.2949 | -0.0411 | -0.1939 |
| HALLMARK_UV_RESPONSE_DN | 0.197 | -0.1552 | -0.0736 |
| HALLMARK_WNT_BETA_CATENIN_SIGNALING | 0.087 | -0.1832 | 0.0367 |
| HALLMARK_APICAL_JUNCTION | 0.1992 | -0.0995 | -0.0901 |
| HALLMARK_MYOGENESIS | 0.1354 | -0.0601 | -0.0451 |
| HALLMARK_ESTROGEN_RESPONSE_EARLY | 0.1069 | -0.0849 | -0.0289 |
| HALLMARK_APICAL_SURFACE | 0.1498 | -0.0613 | -0.0638 |
| HALLMARK_ANGIOGENESIS | 0.2684 | -0.0179 | -0.1339 |
| HALLMARK_ALLOGRAFT_REJECTION | 0.196 | 0.235 | -0.2895 |
| HALLMARK_INTERFERON_GAMMA_RESPONSE | 0.1206 | 0.239 | -0.2476 |
| HALLMARK_INFLAMMATORY_RESPONSE | 0.2583 | 0.1371 | -0.2549 |
| HALLMARK_TNFA_SIGNALING_VIA_NFKB | 0.2307 | 0.0475 | -0.2126 |
| HALLMARK_IL6_JAK_STAT3_SIGNALING | 0.2373 | 0.1291 | -0.2544 |
| HALLMARK_INTERFERON_ALPHA_RESPONSE | 0.0189 | 0.2573 | -0.188 |
| HALLMARK_KRAS_SIGNALING_UP | 0.1956 | 0.067 | -0.1723 |
| HALLMARK_IL2_STAT5_SIGNALING | 0.1894 | 0.0519 | -0.1922 |
| HALLMARK_COMPLEMENT | 0.1702 | 0.1082 | -0.1933 |

| **Mean GSVA scores of upregulation of KEGG gene sets** | | | |
| --- | --- | --- | --- |
|  | **C1** | **C2** | **C3** |
| KEGG_ECM_RECEPTOR_INTERACTION | 0.2903 | -0.1103 | -0.1208 |
| KEGG_CYTOKINE_CYTOKINE_RECEPTOR_INTERACTION | 0.1992 | 0.148 | -0.1863 |
| KEGG_HEMATOPOIETIC_CELL_LINEAGE | 0.2386 | 0.1758 | -0.2393 |
| KEGG_FOCAL_ADHESION | 0.2366 | -0.1363 | -0.0994 |
| KEGG_CHEMOKINE_SIGNALING_PATHWAY | 0.2022 | 0.0617 | -0.1981 |
| KEGG_LEISHMANIA_INFECTION | 0.2441 | 0.1444 | -0.2733 |
| KEGG_CELL_ADHESION_MOLECULES_CAMS | 0.214 | 0.1293 | -0.2134 |
| KEGG_GRAFT_VERSUS_HOST_DISEASE | 0.188 | 0.3724 | -0.3298 |
| KEGG_T_CELL_RECEPTOR_SIGNALING_PATHWAY | 0.1926 | 0.0153 | -0.1894 |
| KEGG_NATURAL_KILLER_CELL_MEDIATED_CYTOTOXICITY | 0.1895 | 0.137 | -0.2101 |
| KEGG_RIBOSOME | -0.3097 | 0.1853 | 0.0625 |
| KEGG_OXIDATIVE_PHOSPHORYLATION | -0.2371 | 0.2245 | -0.0268 |
| KEGG_ANTIGEN_PROCESSING_AND_PRESENTATION | 0.0796 | 0.2768 | -0.1949 |
| KEGG_ALLOGRAFT_REJECTION | 0.1746 | 0.3606 | -0.3271 |
| KEGG_TYPE_I_DIABETES_MELLITUS | 0.1647 | 0.3384 | -0.2873 |
| KEGG_SYSTEMIC_LUPUS_ERYTHEMATOSUS | -0.0694 | 0.2387 | -0.1224 |
| KEGG_PROTEASOME | -0.1809 | 0.266 | -0.1041 |
| KEGG_INTESTINAL_IMMUNE_NETWORK_FOR_IGA_PRODUCTION | 0.1748 | 0.2567 | -0.2629 |
| KEGG_AUTOIMMUNE_THYROID_DISEASE | 0.1558 | 0.315 | -0.2045 |
| KEGG_MATURITY_ONSET_DIABETES_OF_THE_YOUNG | -0.0406 | 0.1708 | 0.0983 |
| KEGG_HEDGEHOG_SIGNALING_PATHWAY | 0.0952 | -0.1394 | 0.0419 |
| KEGG_TASTE_TRANSDUCTION | 0.0005 | -0.0486 | 0.0914 |
| KEGG_PENTOSE_AND_GLUCURONATE_INTERCONVERSIONS | -0.1057 | 0.1676 | 0.0699 |
| KEGG_ASCORBATE_AND_ALDARATE_METABOLISM | -0.0769 | 0.1405 | 0.1018 |
| KEGG_OLFACTORY_TRANSDUCTION | -0.0106 | 0.1686 | 0.0841 |
| KEGG_BASAL_CELL_CARCINOMA | 0.0747 | -0.1514 | 0.0604 |
| KEGG_STARCH_AND_SUCROSE_METABOLISM | 0.0067 | 0.0413 | 0.0511 |

| **Mean GSVA scores of downregulation of KEGG gene sets** | | | |
| --- | --- | --- | --- |
|  | **C1** | **C2** | **C3** |
| KEGG_RIBOSOME | -0.3097 | 0.1853 | 0.0625 |
| KEGG_PENTOSE_AND_GLUCURONATE_INTERCONVERSIONS | -0.1057 | 0.1676 | 0.0699 |
| KEGG_OXIDATIVE_PHOSPHORYLATION | -0.2371 | 0.2245 | -0.0268 |
| KEGG_STARCH_AND_SUCROSE_METABOLISM | 0.0067 | 0.0413 | 0.0511 |
| KEGG_ASCORBATE_AND_ALDARATE_METABOLISM | -0.0769 | 0.1405 | 0.1018 |
| KEGG_MATURITY_ONSET_DIABETES_OF_THE_YOUNG | -0.0406 | 0.1708 | 0.0983 |
| KEGG_CARDIAC_MUSCLE_CONTRACTION | -0.1039 | 0.1242 | 0.0207 |
| KEGG_PARKINSONS_DISEASE | -0.2278 | 0.225 | -0.034 |
| KEGG_HUNTINGTONS_DISEASE | -0.1862 | 0.1279 | -0.0015 |
| KEGG_METABOLISM_OF_XENOBIOTICS_BY_CYTOCHROME_P450 | -0.0745 | 0.1324 | 0.0719 |
| KEGG_ECM_RECEPTOR_INTERACTION | 0.2903 | -0.1103 | -0.1208 |
| KEGG_HEDGEHOG_SIGNALING_PATHWAY | 0.0952 | -0.1394 | 0.0419 |
| KEGG_TASTE_TRANSDUCTION | 0.0005 | -0.0486 | 0.0914 |
| KEGG_BASAL_CELL_CARCINOMA | 0.0747 | -0.1514 | 0.0604 |
| KEGG_AXON_GUIDANCE | 0.1342 | -0.152 | -0.0097 |
| KEGG_FOCAL_ADHESION | 0.2366 | -0.1363 | -0.0994 |
| KEGG_NEUROACTIVE_LIGAND_RECEPTOR_INTERACTION | 0.0977 | 0.0754 | -0.0202 |
| KEGG_DILATED_CARDIOMYOPATHY | 0.1788 | -0.0614 | -0.0569 |
| KEGG_ARRHYTHMOGENIC_RIGHT_VENTRICULAR_CARDIOMYOPATHY_ARVC | 0.1605 | -0.0948 | -0.0355 |
| KEGG_HYPERTROPHIC_CARDIOMYOPATHY_HCM | 0.1586 | -0.051 | -0.0558 |
| KEGG_CYTOKINE_CYTOKINE_RECEPTOR_INTERACTION | 0.1992 | 0.148 | -0.1863 |
| KEGG_GRAFT_VERSUS_HOST_DISEASE | 0.188 | 0.3724 | -0.3298 |
| KEGG_HEMATOPOIETIC_CELL_LINEAGE | 0.2386 | 0.1758 | -0.2393 |
| KEGG_ANTIGEN_PROCESSING_AND_PRESENTATION | 0.0796 | 0.2768 | -0.1949 |
| KEGG_CHEMOKINE_SIGNALING_PATHWAY | 0.2022 | 0.0617 | -0.1981 |
| KEGG_NATURAL_KILLER_CELL_MEDIATED_CYTOTOXICITY | 0.1895 | 0.137 | -0.2101 |
| KEGG_INTESTINAL_IMMUNE_NETWORK_FOR_IGA_PRODUCTION | 0.1748 | 0.2567 | -0.2629 |
| KEGG_TYPE_I_DIABETES_MELLITUS | 0.1647 | 0.3384 | -0.2873 |
| KEGG_ALLOGRAFT_REJECTION | 0.1746 | 0.3606 | -0.3271 |
| KEGG_LEISHMANIA_INFECTION | 0.2441 | 0.1444 | -0.2733 |

| **Mean GSVA scores of upregulation of BP gene sets** | | | |
| --- | --- | --- | --- |
|  | **C1** | **C2** | **C3** |
| GO_EXTRACELLULAR_STRUCTURE_ORGANIZATION | 0.2488 | -0.0513 | -0.1245 |
| GO_LEUKOCYTE_MIGRATION | 0.2298 | 0.1001 | -0.214 |
| GO_COLLAGEN_FIBRIL_ORGANIZATION | 0.3119 | -0.0473 | -0.1905 |
| GO_CELL_CHEMOTAXIS | 0.2301 | 0.116 | -0.2107 |
| GO_MULTICELLULAR_ORGANISMAL_MACROMOLECULE_METABOLIC_PROCESS | 0.2685 | -0.0127 | -0.1348 |
| GO_ANGIOGENESIS | 0.2168 | -0.0715 | -0.119 |
| GO_REGULATION_OF_VASCULATURE_DEVELOPMENT | 0.2158 | 0.0049 | -0.1454 |
| GO_REGULATION_OF_CHEMOTAXIS | 0.22 | 0.037 | -0.1668 |
| GO_POSITIVE_REGULATION_OF_INFLAMMATORY_RESPONSE | 0.2168 | 0.1669 | -0.2242 |
| GO_POSITIVE_REGULATION_OF_VASCULATURE_DEVELOPMENT | 0.2336 | 0.0177 | -0.1665 |
| GO_OXIDATIVE_PHOSPHORYLATION | -0.2911 | 0.2548 | -0.0177 |
| GO_TRANSLATIONAL_TERMINATION | -0.2963 | 0.2336 | -0.0026 |
| GO_MITOCHONDRIAL_RESPIRATORY_CHAIN_COMPLEX_I_BIOGENESIS | -0.3142 | 0.2662 | -0.0086 |
| GO_ELECTRON_TRANSPORT_CHAIN | -0.261 | 0.2298 | -0.0178 |
| GO_MITOCHONDRIAL_TRANSLATION | -0.2816 | 0.2054 | 0.0046 |
| GO_MITOCHONDRIAL_RESPIRATORY_CHAIN_COMPLEX_ASSEMBLY | -0.3043 | 0.2433 | 0.0002 |
| GO_TRANSLATIONAL_ELONGATION | -0.2721 | 0.2065 | 0 |
| GO_ESTABLISHMENT_OF_PROTEIN_LOCALIZATION_TO_ENDOPLASMIC_RETICULUM | -0.2913 | 0.1685 | 0.0575 |
| GO_ANAPHASE_PROMOTING_COMPLEX_DEPENDENT_CATABOLIC_PROCESS | -0.2034 | 0.2081 | -0.055 |
| GO_RESPONSE_TO_INTERFERON_GAMMA | 0.15 | 0.2213 | -0.2394 |
| GO_MICROTUBULE_BUNDLE_FORMATION | -0.1336 | -0.1042 | 0.1095 |
| GO_AXONEME_ASSEMBLY | -0.1883 | -0.0561 | 0.126 |
| GO_CILIUM_MOVEMENT | -0.2175 | -0.0368 | 0.1291 |
| GO_CILIUM_ORGANIZATION | -0.1368 | -0.0806 | 0.0906 |
| GO_CILIUM_MORPHOGENESIS | -0.1218 | -0.0921 | 0.0872 |
| GO_ENDOCRINE_SYSTEM_DEVELOPMENT | 0.0469 | -0.0239 | 0.0598 |
| GO_AXONEMAL_DYNEIN_COMPLEX_ASSEMBLY | -0.1855 | -0.0327 | 0.1251 |
| GO_MICROTUBULE_BASED_MOVEMENT | -0.0895 | -0.0885 | 0.0671 |
| GO_ENTEROENDOCRINE_CELL_DIFFERENTIATION | 0.0393 | -0.0715 | 0.1109 |

| **Mean GSVA scores of downregulation of BP gene sets** | | | |
| --- | --- | --- | --- |
|  | **C1** | **C2** | **C3** |
| GO_ESTABLISHMENT_OF_PROTEIN_LOCALIZATION_TO_ENDOPLASMIC_RETICULUM | -0.2913 | 0.1685 | 0.0575 |
| GO_PROTEIN_LOCALIZATION_TO_ENDOPLASMIC_RETICULUM | -0.2579 | 0.1416 | 0.0493 |
| GO_MULTI_ORGANISM_METABOLIC_PROCESS | -0.2453 | 0.0799 | 0.0803 |
| GO_NUCLEAR_TRANSCRIBED_MRNA_CATABOLIC_PROCESS_NONSENSE_MEDIATED_DECAY | -0.2602 | 0.1095 | 0.0759 |
| GO_RRNA_METABOLIC_PROCESS | -0.2341 | 0.0954 | 0.062 |
| GO_RIBOSOME_BIOGENESIS | -0.2261 | 0.084 | 0.0619 |
| GO_TRANSLATIONAL_INITIATION | -0.2511 | 0.1211 | 0.066 |
| GO_OXIDATIVE_PHOSPHORYLATION | -0.2911 | 0.2548 | -0.0177 |
| GO_TRANSLATIONAL_TERMINATION | -0.2963 | 0.2336 | -0.0026 |
| GO_MITOCHONDRIAL_TRANSLATION | -0.2816 | 0.2054 | 0.0046 |
| GO_KERATINOCYTE_DIFFERENTIATION | 0.0603 | 0.0401 | 0.0336 |
| GO_PEPTIDE_CROSS_LINKING | 0.1034 | 0.1044 | 0.0068 |
| GO_EPIDERMAL_CELL_DIFFERENTIATION | 0.0425 | 0.0084 | 0.0414 |
| GO_KERATINIZATION | 0.0016 | 0.1073 | 0.0718 |
| GO_SKIN_DEVELOPMENT | 0.0884 | -0.0284 | 0.0261 |
| GO_EPIDERMIS_DEVELOPMENT | 0.0621 | -0.0091 | 0.0299 |
| GO_EPITHELIAL_CELL_DIFFERENTIATION | 0.0742 | -0.0218 | 0.0009 |
| GO_NEURON_PROJECTION_GUIDANCE | 0.1188 | -0.095 | 0.0235 |
| GO_NEPHRON_DEVELOPMENT | 0.1549 | -0.0747 | -0.0182 |
| GO_HOMOPHILIC_CELL_ADHESION_VIA_PLASMA_MEMBRANE_ADHESION_MOLECULES | 0.1047 | -0.0351 | 0.0229 |
| GO_ADAPTIVE_IMMUNE_RESPONSE | 0.2063 | 0.1755 | -0.2432 |
| GO_POSITIVE_REGULATION_OF_CELL_ACTIVATION | 0.2049 | 0.1293 | -0.231 |
| GO_INFLAMMATORY_RESPONSE | 0.2099 | 0.1091 | -0.1905 |
| GO_CELL_CHEMOTAXIS | 0.2301 | 0.116 | -0.2107 |
| GO_REGULATION_OF_CELL_ACTIVATION | 0.1894 | 0.1115 | -0.2133 |
| GO_POSITIVE_REGULATION_OF_DEFENSE_RESPONSE | 0.1388 | 0.1359 | -0.1949 |
| GO_POSITIVE_REGULATION_OF_INFLAMMATORY_RESPONSE | 0.2168 | 0.1669 | -0.2242 |
| GO_LEUKOCYTE_MIGRATION | 0.2298 | 0.1001 | -0.214 |
| GO_REGULATION_OF_LEUKOCYTE_MEDIATED_IMMUNITY | 0.1975 | 0.139 | -0.238 |
| GO_REGULATION_OF_HOMOTYPIC_CELL_CELL_ADHESION | 0.1834 | 0.1239 | -0.2205 |

| **Mean GSVA scores of upregulation of CC gene sets** | | | |
| --- | --- | --- | --- |
|  | **C1** | **C2** | **C3** |
| GO_PROTEINACEOUS_EXTRACELLULAR_MATRIX | 0.1973 | -0.0433 | -0.058 |
| GO_EXTRACELLULAR_MATRIX | 0.1847 | -0.036 | -0.0592 |
| GO_EXTRACELLULAR_MATRIX_COMPONENT | 0.2301 | -0.093 | -0.0764 |
| GO_EXTERNAL_SIDE_OF_PLASMA_MEMBRANE | 0.2254 | 0.1368 | -0.2198 |
| GO_COLLAGEN_TRIMER | 0.2332 | 0.0043 | -0.114 |
| GO_SIDE_OF_MEMBRANE | 0.1986 | 0.075 | -0.1861 |
| GO_COMPLEX_OF_COLLAGEN_TRIMERS | 0.3406 | -0.0833 | -0.1335 |
| GO_PROTEIN_COMPLEX_INVOLVED_IN_CELL_ADHESION | 0.3247 | -0.0491 | -0.2073 |
| GO_IMMUNOLOGICAL_SYNAPSE | 0.2326 | 0.1275 | -0.2823 |
| GO_BASEMENT_MEMBRANE | 0.2052 | -0.1041 | -0.0573 |
| GO_RESPIRATORY_CHAIN | -0.2897 | 0.258 | -0.0193 |
| GO_RIBOSOMAL_SUBUNIT | -0.3021 | 0.2134 | 0.0458 |
| GO_RIBOSOME | -0.28 | 0.1945 | 0.0408 |
| GO_INNER_MITOCHONDRIAL_MEMBRANE_PROTEIN_COMPLEX | -0.2849 | 0.2483 | -0.0114 |
| GO_LARGE_RIBOSOMAL_SUBUNIT | -0.3087 | 0.2182 | 0.0448 |
| GO_CYTOSOLIC_RIBOSOME | -0.285 | 0.17 | 0.0589 |
| GO_NADH_DEHYDROGENASE_COMPLEX | -0.328 | 0.2947 | -0.0236 |
| GO_MITOCHONDRIAL_PROTEIN_COMPLEX | -0.26 | 0.1985 | -0.0021 |
| GO_ORGANELLAR_RIBOSOME | -0.3173 | 0.2565 | -0.0023 |
| GO_MITOCHONDRIAL_MEMBRANE_PART | -0.2258 | 0.1729 | -0.0107 |
| GO_CILIARY_PLASM | -0.1252 | -0.1239 | 0.1076 |
| GO_CILIARY_PART | -0.0884 | -0.0878 | 0.0784 |
| GO_CILIUM | -0.0705 | -0.0779 | 0.0702 |
| GO_AXONEME_PART | -0.2054 | -0.0876 | 0.1561 |
| GO_MOTILE_CILIUM | -0.1219 | -0.0205 | 0.0832 |
| GO_AXONEMAL_DYNEIN_COMPLEX | -0.2015 | -0.0723 | 0.169 |
| GO_PHOTORECEPTOR_INNER_SEGMENT | -0.0369 | -0.049 | 0.0808 |
| GO_PRESYNAPTIC_ACTIVE_ZONE | 0.0343 | -0.1233 | 0.1032 |
| GO_SPERM_FLAGELLUM | -0.103 | -0.0344 | 0.063 |

| **Mean GSVA scores of downregulation of CC gene sets** | | | |
| --- | --- | --- | --- |
|  | **C1** | **C2** | **C3** |
| GO_RIBOSOMAL_SUBUNIT | -0.3021 | 0.2134 | 0.0458 |
| GO_RIBOSOME | -0.28 | 0.1945 | 0.0408 |
| GO_CYTOSOLIC_RIBOSOME | -0.285 | 0.17 | 0.0589 |
| GO_LARGE_RIBOSOMAL_SUBUNIT | -0.3087 | 0.2182 | 0.0448 |
| GO_CYTOSOLIC_LARGE_RIBOSOMAL_SUBUNIT | -0.3062 | 0.1677 | 0.0656 |
| GO_SMALL_RIBOSOMAL_SUBUNIT | -0.2952 | 0 | 0.0413 |
| GO_RESPIRATORY_CHAIN | -0.2897 | 0.258 | -0.0193 |
| GO_INNER_MITOCHONDRIAL_MEMBRANE_PROTEIN_COMPLEX | -0.2849 | 0.2483 | -0.0114 |
| GO_ORGANELLAR_RIBOSOME | -0.3173 | 0.2565 | -0.0023 |
| GO_MITOCHONDRIAL_PROTEIN_COMPLEX | -0.26 | 0.1985 | -0.0021 |
| GO_CORNIFIED_ENVELOPE | 0.0048 | 0.1266 | 0.0751 |
| GO_PROTEINACEOUS_EXTRACELLULAR_MATRIX | 0.1973 | -0.0433 | -0.058 |
| GO_EXTRACELLULAR_MATRIX | 0.1847 | -0.036 | -0.0592 |
| GO_EXTRACELLULAR_MATRIX_COMPONENT | 0.2301 | -0.093 | -0.0764 |
| GO_MAIN_AXON | 0.1521 | -0.1268 | 0.0182 |
| GO_COMPLEX_OF_COLLAGEN_TRIMERS | 0.3406 | -0.0833 | -0.1335 |
| GO_PRESYNAPTIC_MEMBRANE | 0.0929 | -0.0988 | 0.0367 |
| GO_CATION_CHANNEL_COMPLEX | 0.0675 | -0.026 | 0.0203 |
| GO_NEURON_SPINE | 0.0858 | -0.0961 | 0.0013 |
| GO_POTASSIUM_CHANNEL_COMPLEX | 0.0581 | -0.024 | 0.0259 |
| GO_EXTERNAL_SIDE_OF_PLASMA_MEMBRANE | 0.2254 | 0.1368 | -0.2198 |
| GO_SIDE_OF_MEMBRANE | 0.1986 | 0.075 | -0.1861 |
| GO_MHC_PROTEIN_COMPLEX | 0.1149 | 0.3768 | -0.3023 |
| GO_MHC_CLASS_II_PROTEIN_COMPLEX | 0.1142 | 0.3918 | -0.3192 |
| GO_LUMENAL_SIDE_OF_MEMBRANE | 0.1133 | 0.2306 | -0.2564 |
| GO_PHAGOCYTIC_VESICLE | 0.169 | 0.0807 | -0.2013 |
| GO_IMMUNOLOGICAL_SYNAPSE | 0.2326 | 0.1275 | -0.2823 |
| GO_ENDOCYTIC_VESICLE_MEMBRANE | 0.1425 | 0.0558 | -0.1546 |
| GO_T_CELL_RECEPTOR_COMPLEX | 0.1834 | 0.2965 | -0.3365 |
| GO_COLLAGEN_TRIMER | 0.2332 | 0.0043 | -0.114 |

| **Mean GSVA scores of upregulation of MF gene sets** | | | |
| --- | --- | --- | --- |
|  | **C1** | **C2** | **C3** |
| GO_COLLAGEN_BINDING | 0.3186 | -0.0487 | -0.1908 |
| GO_INTEGRIN_BINDING | 0.2407 | -0.0826 | -0.1322 |
| GO_CELL_ADHESION_MOLECULE_BINDING | 0.2143 | -0.0598 | -0.1151 |
| GO_CYTOKINE_BINDING | 0.2549 | 0.068 | -0.2156 |
| GO_GROWTH_FACTOR_BINDING | 0.2006 | -0.0651 | -0.1054 |
| GO_CYTOKINE_RECEPTOR_ACTIVITY | 0.2318 | 0.1241 | -0.2124 |
| GO_CHEMOKINE_RECEPTOR_BINDING | 0.1467 | 0.2632 | -0.2298 |
| GO_G_PROTEIN_COUPLED_CHEMOATTRACTANT_RECEPTOR_ACTIVITY | 0.2936 | 0.1762 | -0.2927 |
| GO_CORECEPTOR_ACTIVITY | 0.2331 | 0.0558 | -0.2084 |
| GO_CHEMOKINE_ACTIVITY | 0.1486 | 0.2795 | -0.216 |
| GO_OXIDOREDUCTASE_ACTIVITY_ACTING_ON_NAD_P_H_QUINONE_OR_SIMILAR_COMPOUND_AS_ACCEPTOR | -0.3029 | 0.2763 | -0.0232 |
| GO_STRUCTURAL_CONSTITUENT_OF_RIBOSOME | -0.2726 | 0.1859 | 0.0411 |
| GO_CCR_CHEMOKINE_RECEPTOR_BINDING | 0.16 | 0.297 | -0.278 |
| GO_ANTIGEN_BINDING | 0.1631 | 0.2091 | -0.2414 |
| GO_OXIDOREDUCTASE_ACTIVITY_ACTING_ON_NAD_P_H | -0.2043 | 0.2172 | -0.0437 |
| GO_THREONINE_TYPE_PEPTIDASE_ACTIVITY | -0.2439 | 0.3328 | -0.12 |
| GO_PEPTIDE_ANTIGEN_BINDING | 0.1251 | 0.2661 | -0.231 |
| GO_OXIDOREDUCTASE_ACTIVITY_ACTING_ON_A_HEME_GROUP_OF_DONORS | -0.2725 | 0.2173 | -0.0021 |
| GO_BITTER_TASTE_RECEPTOR_ACTIVITY | -0.0404 | -0.0408 | 0.1053 |
| GO_TASTE_RECEPTOR_ACTIVITY | -0.029 | -0.012 | 0.085 |
| GO_MICROTUBULE_MOTOR_ACTIVITY | -0.0689 | -0.1005 | 0.0806 |
| GO_OLFACTORY_RECEPTOR_ACTIVITY | -0.0254 | 0.1801 | 0.0959 |
| GO_STRUCTURAL_CONSTITUENT_OF_EYE_LENS | -0.0416 | 0.1168 | 0.0428 |
| GO_ANION_CATION_SYMPORTER_ACTIVITY | 0.061 | -0.0275 | 0.0475 |
| GO_MOTOR_ACTIVITY | -0.0182 | -0.0868 | 0.0582 |
| GO_INORGANIC_ANION_TRANSMEMBRANE_TRANSPORTER_ACTIVITY | 0.0358 | -0.0164 | 0.037 |
| GO_RETINOIC_ACID_BINDING | -0.009 | 0.0895 | 0.0928 |

| **Mean GSVA scores of downregulation of MF gene sets** | | | |
| --- | --- | --- | --- |
|  | **C1** | **C2** | **C3** |
| GO_STRUCTURAL_CONSTITUENT_OF_RIBOSOME | -0.2726 | 0.1859 | 0.0411 |
| GO_OXIDOREDUCTASE_ACTIVITY_ACTING_ON_NAD_P_H_QUINONE_OR_SIMILAR_COMPOUND_AS_ACCEPTOR | -0.3029 | 0.2763 | -0.0232 |
| GO_RRNA_BINDING | -0.2449 | 0.1322 | 0.0455 |
| GO_OXIDOREDUCTASE_ACTIVITY_ACTING_ON_A_HEME_GROUP_OF_DONORS | -0.2725 | 0.2173 | -0.0021 |
| GO_OXIDOREDUCTASE_ACTIVITY_ACTING_ON_NAD_P_H | -0.2043 | 0.2172 | -0.0437 |
| GO_NEUROPEPTIDE_RECEPTOR_BINDING | -0.0751 | 0.1203 | 0.0953 |
| GO_HORMONE_ACTIVITY | -0.0094 | 0.1272 | 0.0433 |
| GO_GLUCURONOSYLTRANSFERASE_ACTIVITY | 0.0068 | 0.0859 | 0.0599 |
| GO_THREONINE_TYPE_PEPTIDASE_ACTIVITY | -0.2439 | 0.3328 | -0.12 |
| GO_NEUROPEPTIDE_HORMONE_ACTIVITY | -0.0176 | 0.118 | 0.0704 |
| GO_BINDING_BRIDGING | 0.1319 | -0.0836 | -0.0646 |
| GO_EXTRACELLULAR_MATRIX_STRUCTURAL_CONSTITUENT | 0.2082 | -0.0278 | -0.0478 |
| GO_VOLTAGE_GATED_ION_CHANNEL_ACTIVITY | 0.0689 | -0.0145 | 0.0187 |
| GO_GATED_CHANNEL_ACTIVITY | 0.0776 | -0.0033 | 0.0131 |
| GO_SODIUM_ION_TRANSMEMBRANE_TRANSPORTER_ACTIVITY | 0.0783 | -0.0391 | 0.0384 |
| GO_VOLTAGE_GATED_CATION_CHANNEL_ACTIVITY | 0.0768 | -0.0042 | 0.0197 |
| GO_RHO_GUANYL_NUCLEOTIDE_EXCHANGE_FACTOR_ACTIVITY | 0.1642 | -0.2305 | 0.0014 |
| GO_GLUTAMATE_RECEPTOR_ACTIVITY | 0.0396 | -0.011 | 0.0698 |
| GO_CATION_CHANNEL_ACTIVITY | 0.0851 | -0.0081 | 0.0028 |
| GO_NEUROPEPTIDE_BINDING | 0.0245 | 0.0856 | 0.1014 |
| GO_ANTIGEN_BINDING | 0.1631 | 0.2091 | -0.2414 |
| GO_CHEMOKINE_RECEPTOR_BINDING | 0.1467 | 0.2632 | -0.2298 |
| GO_CHEMOKINE_ACTIVITY | 0.1486 | 0.2795 | -0.216 |
| GO_CYTOKINE_ACTIVITY | 0.1243 | 0.1692 | -0.1154 |
| GO_CCR_CHEMOKINE_RECEPTOR_BINDING | 0.16 | 0.297 | -0.278 |
| GO_CYTOKINE_RECEPTOR_BINDING | 0.1316 | 0.11 | -0.1214 |
| GO_COLLAGEN_BINDING | 0.3186 | -0.0487 | -0.1908 |
| GO_CYTOKINE_BINDING | 0.2549 | 0.068 | -0.2156 |
| GO_CYTOKINE_RECEPTOR_ACTIVITY | 0.2318 | 0.1241 | -0.2124 |
| GO_MHC_PROTEIN_BINDING | 0.2066 | 0.2297 | -0.305 |
