# Supplementary material for: Tumor Immunometabolism Characterization in Ovarian Cancer With Prognostic and Therapeutic Implications
Source: Front Oncol. 2021 Mar 16;11:622752. doi: 10.3389/fonc.2021.622752 (PMC8008085; doi:10.3389/fonc.2021.622752)
Supplement: Supplementary file 16 [file Table_7.doc]

**Supplementary Table S7: The prognostic value of immune and stromal cells enrichment scores in TCGA OV OS**

| **Immune Cells** | **HR (95%CI)** | **z** | ***P* value** | **Group** |
| --- | --- | --- | --- | --- |
| B cells memory | 1.0628(0.8095-1.3953) | 0.4382 | 0.6612 | C1 |
| B cells naive | 0.9268(0.7301-1.1765) | -0.6245 | 0.5323 | C1 |
| Dendritic cells activated | 1.0182(0.796-1.3025) | 0.144 | 0.8855 | C1 |
| Dendritic cells resting | 1.1301(0.8495-1.5034) | 0.8398 | 0.401 | C1 |
| Endothelial cells | 1.0244(0.8276-1.2681) | 0.2217 | 0.8246 | C1 |
| Eosinophils | 1.0548(0.7973-1.3955) | 0.3737 | 0.7087 | C1 |
| Fibroblasts | 1.2404(0.8392-1.8334) | 1.0806 | 0.2799 | C1 |
| Macrophages M0 | 1.0325(0.7847-1.3585) | 0.2285 | 0.8193 | C1 |
| Macrophages M1 | 0.947(0.7331-1.2234) | -0.4164 | 0.6771 | C1 |
| Macrophages M2 | 0.9629(0.739-1.2546) | -0.2803 | 0.7792 | C1 |
| Mast cells activated | 0.9556(0.7702-1.1857) | -0.4122 | 0.6802 | C1 |
| Mast cells resting | 0.9368(0.706-1.2432) | -0.4519 | 0.6513 | C1 |
| MDSC | 0.9648(0.7049-1.3204) | -0.2241 | 0.8227 | C1 |
| Monocytes | 0.9221(0.7284-1.1674) | -0.6736 | 0.5006 | C1 |
| Neutrophils | 1.0967(0.8835-1.3613) | 0.8368 | 0.4027 | C1 |
| NK cells activated | 0.9776(0.7742-1.2345) | -0.1901 | 0.8492 | C1 |
| NK cells resting | 0.8943(0.7-1.1426) | -0.8934 | 0.3716 | C1 |
| Plasma cells | 1.0661(0.8468-1.3422) | 0.5444 | 0.5862 | C1 |
| T cells CD4 memory activated | 0.9772(0.7796-1.2248) | -0.2004 | 0.8411 | C1 |
| T cells CD4 memory resting | 1.0167(0.8119-1.2731) | 0.144 | 0.8855 | C1 |
| T cells CD4 naive | 0.8438(0.6621-1.0754) | -1.3727 | 0.1699 | C1 |
| T cells CD8 | 0.9447(0.7601-1.1743) | -0.5124 | 0.6084 | C1 |
| T cells follicular helper | 0.9371(0.7586-1.1576) | -0.6022 | 0.547 | C1 |
| T cells gamma delta | 0.8271(0.6456-1.0597) | -1.5013 | 0.1333 | C1 |
| Tregs | 0.963(0.7597-1.2208) | -0.3113 | 0.7555 | C1 |
| B cells memory | 1.139(0.8381-1.5479) | 0.8317 | 0.4056 | C2 |
| B cells naive | 0.8846(0.6676-1.1721) | -0.8541 | 0.3931 | C2 |
| Dendritic cells activated | 0.7255(0.4693-1.1216) | -1.4437 | 0.1488 | C2 |
| Dendritic cells resting | 0.8755(0.572-1.3402) | -0.6119 | 0.5406 | C2 |
| Endothelial cells | 0.5116(0.3227-0.811) | -2.8511 | 0.0044 | C2 |
| Eosinophils | 0.8407(0.5814-1.2156) | -0.9224 | 0.3563 | C2 |
| Fibroblasts | 0.7405(0.5545-0.9889) | -2.0357 | 0.0418 | C2 |
| Macrophages M0 | 0.553(0.3663-0.8348) | -2.8195 | 0.0048 | C2 |
| Macrophages M1 | 0.595(0.3763-0.9408) | -2.2211 | 0.0263 | C2 |
| Macrophages M2 | 0.8587(0.5903-1.2492) | -0.7965 | 0.4257 | C2 |
| Mast cells activated | 1.0295(0.7603-1.394) | 0.188 | 0.8508 | C2 |
| Mast cells resting | 0.6633(0.4791-0.9183) | -2.4738 | 0.0134 | C2 |
| MDSC | 0.8169(0.5217-1.2792) | -0.8838 | 0.3768 | C2 |
| Monocytes | 0.862(0.6032-1.232) | -0.8148 | 0.4152 | C2 |
| Neutrophils | 1.0964(0.7195-1.6708) | 0.4282 | 0.6685 | C2 |
| NK cells activated | 0.8856(0.6506-1.2054) | -0.7722 | 0.44 | C2 |
| NK cells resting | 0.9732(0.7277-1.3016) | -0.1828 | 0.855 | C2 |
| Plasma cells | 0.9861(0.7455-1.3043) | -0.098 | 0.922 | C2 |
| T cells CD4 memory activated | 0.9701(0.6751-1.394) | -0.1639 | 0.8698 | C2 |
| T cells CD4 memory resting | 0.83(0.6149-1.1204) | -1.2173 | 0.2235 | C2 |
| T cells CD4 naive | 0.6807(0.4924-0.9409) | -2.3286 | 0.0199 | C2 |
| T cells CD8 | 0.9261(0.6259-1.3704) | -0.384 | 0.7009 | C2 |
| T cells follicular helper | 0.7059(0.511-0.975) | -2.1137 | 0.0345 | C2 |
| T cells gamma delta | 0.9581(0.724-1.2678) | -0.2995 | 0.7646 | C2 |
| Tregs | 0.8513(0.6104-1.1873) | -0.9485 | 0.3429 | C2 |
| B cells memory | 1.0286(0.8679-1.2191) | 0.3251 | 0.7451 | C3 |
| B cells naive | 0.8978(0.7591-1.0617) | -1.2602 | 0.2076 | C3 |
| Dendritic cells activated | 1.2361(0.9472-1.6129) | 1.5608 | 0.1186 | C3 |
| Dendritic cells resting | 1.2014(0.9495-1.5202) | 1.5281 | 0.1265 | C3 |
| Endothelial cells | 0.9169(0.7098-1.1844) | -0.6642 | 0.5066 | C3 |
| Eosinophils | 1.4049(1.0747-1.8366) | 2.4867 | 0.0129 | C3 |
| Fibroblasts | 1.0728(0.8563-1.344) | 0.611 | 0.5412 | C3 |
| Macrophages M0 | 1.1568(0.9385-1.4259) | 1.365 | 0.1723 | C3 |
| Macrophages M1 | 1.1716(0.8981-1.5284) | 1.1677 | 0.2429 | C3 |
| Macrophages M2 | 1.4995(1.1338-1.9833) | 2.84 | 0.0045 | C3 |
| Mast cells activated | 1.1572(0.9564-1.4002) | 1.5017 | 0.1332 | C3 |
| Mast cells resting | 1.2442(1.0126-1.5289) | 2.0793 | 0.0376 | C3 |
| MDSC | 1.3112(0.9937-1.7303) | 1.915 | 0.0555 | C3 |
| Monocytes | 1.1436(0.8599-1.5209) | 0.9227 | 0.3562 | C3 |
| Neutrophils | 1.2918(0.9839-1.696) | 1.8431 | 0.0653 | C3 |
| NK cells activated | 1.0446(0.8215-1.3284) | 0.3562 | 0.7217 | C3 |
| NK cells resting | 1.0736(0.8885-1.2973) | 0.7356 | 0.462 | C3 |
| Plasma cells | 0.8571(0.6667-1.102) | -1.2024 | 0.2292 | C3 |
| T cells CD4 memory activated | 0.9566(0.7932-1.1537) | -0.4642 | 0.6425 | C3 |
| T cells CD4 memory resting | 1.1261(0.9137-1.3879) | 1.1137 | 0.2654 | C3 |
| T cells CD4 naive | 1.0986(0.8997-1.3414) | 0.9229 | 0.356 | C3 |
| T cells CD8 | 1.0337(0.8096-1.32) | 0.2659 | 0.7903 | C3 |
| T cells follicular helper | 0.8688(0.6804-1.1095) | -1.127 | 0.2598 | C3 |
| T cells gamma delta | 1.0288(0.8587-1.2326) | 0.3077 | 0.7583 | C3 |
| Tregs | 1.2407(0.9987-1.5414) | 1.9479 | 0.0514 | C3 |
| T cells follicular helper | 0.7992(0.697-0.916) | -3.2092 | 0.0013 | all |
| Plasma cells | 0.8738(0.763-1) | -1.9585 | 0.0502 | all |
| B cells naive | 0.889(0.787-1.005) | -1.8837 | 0.0596 | all |
| T cells CD4 memory activated | 0.8916(0.784-1.014) | -1.7447 | 0.081 | all |
| Macrophages M1 | 0.8945(0.789-1.014) | -1.7378 | 0.0823 | all |
| T cells gamma delta | 0.8972(0.793-1.015) | -1.7244 | 0.0846 | all |
| Neutrophils | 1.1175(0.978-1.277) | 1.6268 | 0.1038 | all |
| T cells CD8 | 0.9175(0.801-1.05) | -1.2471 | 0.2124 | all |
| T cells CD4 naive | 0.9396(0.823-1.073) | -0.9223 | 0.3563 | all |
| T cells CD4 memory resting | 1.0595(0.928-1.209) | 0.8582 | 0.3908 | all |
| Eosinophils | 1.0553(0.925-1.204) | 0.8028 | 0.4221 | all |
| Dendritic cells activated | 0.9541(0.836-1.089) | -0.6952 | 0.4869 | all |
| NK cells activated | 0.9572(0.843-1.086) | -0.6783 | 0.4976 | all |
| Mast cells activated | 1.0406(0.918-1.18) | 0.6198 | 0.5354 | all |
| Dendritic cells resting | 1.0438(0.911-1.196) | 0.6186 | 0.5361 | all |
| Monocytes | 0.9585(0.835-1.1) | -0.6035 | 0.5462 | all |
| Fibroblasts | 1.0307(0.893-1.19) | 0.4128 | 0.6797 | all |
| Macrophages M2 | 1.024(0.901-1.164) | 0.3622 | 0.7172 | all |
| NK cells resting | 1.0217(0.897-1.164) | 0.3217 | 0.7477 | all |
| Macrophages M0 | 1.0183(0.892-1.162) | 0.2688 | 0.7881 | all |
| B cells memory | 1.0157(0.893-1.155) | 0.2376 | 0.8122 | all |
| MDSC | 1.0148(0.888-1.16) | 0.2156 | 0.8293 | all |
| Mast cells resting | 1.0142(0.888-1.158) | 0.209 | 0.8344 | all |
| Tregs | 0.9885(0.87-1.123) | -0.1774 | 0.8592 | all |
| Endothelial cells | 1.0099(0.872-1.17) | 0.1307 | 0.896 | all |
