# Supplementary material for: Tumor Immunometabolism Characterization in Ovarian Cancer With Prognostic and Therapeutic Implications
Source: Front Oncol. 2021 Mar 16;11:622752. doi: 10.3389/fonc.2021.622752 (PMC8008085; doi:10.3389/fonc.2021.622752)
Supplement: Supplementary file 17 [file Table_8.doc]

**Supplementary Table S8: The prognostic value of metabolic pathways in TCGA OV OS**

| **Metabolic Pathway** | **HR (95%CI)** | **z** | ***P* value** | **Group** |
| --- | --- | --- | --- | --- |
| alpha Linoleic Acid Metabolism | 0.8976(0.7045-1.1436) | -0.8743 | 0.382 | C1 |
| Biotin Metabolism | 0.8875(0.6916-1.1391) | -0.9371 | 0.3487 | C1 |
| Caffeine Metabolism | 0.9265(0.758-1.1325) | -0.7452 | 0.4561 | C1 |
| Cardiolipin Biosynthesis | 0.795(0.6133-1.0306) | -1.7325 | 0.0832 | C1 |
| Fatty Acid Degradation | 0.9526(0.7557-1.2007) | -0.4113 | 0.6808 | C1 |
| Folate One Carbon Metabolism | 0.9895(0.753-1.3002) | -0.0761 | 0.9393 | C1 |
| Gluconeogenesis | 1.0894(0.8516-1.3935) | 0.6815 | 0.4955 | C1 |
| Glycogen Biosynthesis | 1.3338(1.0259-1.7341) | 2.1506 | 0.0315 | C1 |
| Lipoic Acid Metabolism | 0.7084(0.5168-0.9709) | -2.1433 | 0.0321 | C1 |
| Lysine Degradation | 0.9675(0.7526-1.2439) | -0.2576 | 0.7968 | C1 |
| Oxidative Phosphorylation | 0.9682(0.7646-1.2261) | -0.2679 | 0.7888 | C1 |
| Pentose and Glucuronate Interconversions | 1.0487(0.7897-1.3926) | 0.3284 | 0.7426 | C1 |
| Phenylalanine Tyrosine and Tryptophan Biosynthesis | 0.9835(0.743-1.302) | -0.1161 | 0.9076 | C1 |
| Polyamine Biosynthesis | 0.8046(0.6391-1.0129) | -1.851 | 0.0642 | C1 |
| Primary Bile Acid Biosynthesis | 1.2521(0.9583-1.6358) | 1.6481 | 0.0993 | C1 |
| Propanoate Metabolism | 0.9605(0.7645-1.2067) | -0.346 | 0.7293 | C1 |
| Prostaglandin Biosynthesis | 1.062(0.8336-1.353) | 0.487 | 0.6263 | C1 |
| Pyruvate Metabolism | 1.0245(0.8175-1.2839) | 0.2104 | 0.8334 | C1 |
| Remethylation | 0.7499(0.5645-0.9961) | -1.9867 | 0.047 | C1 |
| Retinoid Metabolism | 1.0542(0.8343-1.332) | 0.4419 | 0.6586 | C1 |
| Riboflavin Metabolism | 0.9501(0.7462-1.2097) | -0.4153 | 0.6779 | C1 |
| Sirtuin Nicotinamide Metabolism | 0.8102(0.6168-1.0641) | -1.5134 | 0.1302 | C1 |
| Ubiquinone and other Terpenoid Quinone Biosynthesis | 1.2018(0.9486-1.5227) | 1.5228 | 0.1278 | C1 |
| Vitamin K | 1.4833(1.1262-1.9536) | 2.806 | 0.005 | C1 |
| alpha Linoleic Acid Metabolism | 0.9277(0.6876-1.2516) | -0.4911 | 0.6233 | C2 |
| Biotin Metabolism | 1.5546(1.1287-2.1411) | 2.7013 | 0.0069 | C2 |
| Caffeine Metabolism | 1.3962(1.0745-1.8143) | 2.4976 | 0.0125 | C2 |
| Cardiolipin Biosynthesis | 1.4976(1.1116-2.0178) | 2.6556 | 0.0079 | C2 |
| Fatty Acid Degradation | 1.4031(1.0021-1.9646) | 1.9724 | 0.0486 | C2 |
| Folate One Carbon Metabolism | 1.0651(0.7832-1.4485) | 0.4019 | 0.6877 | C2 |
| Gluconeogenesis | 1.0666(0.761-1.495) | 0.3744 | 0.7081 | C2 |
| Glycogen Biosynthesis | 0.832(0.5885-1.1763) | -1.0409 | 0.2979 | C2 |
| Lipoic Acid Metabolism | 1.3835(1.0076-1.8997) | 2.0066 | 0.0448 | C2 |
| Lysine Degradation | 0.6124(0.4584-0.8179) | -3.3206 | 9.00E-04 | C2 |
| Oxidative Phosphorylation | 1.4681(1.0169-2.1193) | 2.0497 | 0.0404 | C2 |
| Pentose and Glucuronate Interconversions | 1.2257(0.9234-1.6269) | 1.4086 | 0.159 | C2 |
| Phenylalanine Tyrosine and Tryptophan Biosynthesis | 0.6407(0.4296-0.9556) | -2.1829 | 0.029 | C2 |
| Polyamine Biosynthesis | 0.7432(0.5219-1.0585) | -1.6449 | 0.1 | C2 |
| Primary Bile Acid Biosynthesis | 1.3455(1.0048-1.8016) | 1.9922 | 0.0463 | C2 |
| Propanoate Metabolism | 1.3512(0.9683-1.8854) | 1.7706 | 0.0766 | C2 |
| Prostaglandin Biosynthesis | 1.5058(1.0795-2.1003) | 2.4108 | 0.0159 | C2 |
| Pyruvate Metabolism | 1.3804(1.018-1.872) | 2.0745 | 0.038 | C2 |
| Remethylation | 0.9302(0.6672-1.2968) | -0.427 | 0.6694 | C2 |
| Retinoid Metabolism | 1.0484(0.8076-1.3609) | 0.3547 | 0.7228 | C2 |
| Riboflavin Metabolism | 1.7149(1.2193-2.4119) | 3.0995 | 0.0019 | C2 |
| Sirtuin Nicotinamide Metabolism | 1.485(1.0352-2.1302) | 2.1481 | 0.0317 | C2 |
| Ubiquinone and other Terpenoid Quinone Biosynthesis | 1.4609(1.0369-2.0583) | 2.1672 | 0.0302 | C2 |
| Vitamin K | 1.0216(0.7252-1.4392) | 0.1222 | 0.9027 | C2 |
| alpha Linoleic Acid Metabolism | 0.8172(0.6818-0.9794) | -2.185 | 0.0289 | C3 |
| Biotin Metabolism | 0.9983(0.8238-1.2098) | -0.0173 | 0.9862 | C3 |
| Caffeine Metabolism | 1.19(0.994-1.4248) | 1.8942 | 0.0582 | C3 |
| Cardiolipin Biosynthesis | 1.0005(0.8265-1.2112) | 0.0055 | 0.9956 | C3 |
| Fatty Acid Degradation | 1.0797(0.9168-1.2716) | 0.9187 | 0.3583 | C3 |
| Folate One Carbon Metabolism | 1.2247(1.0139-1.4794) | 2.1034 | 0.0354 | C3 |
| Gluconeogenesis | 1.1737(1.0077-1.3671) | 2.0579 | 0.0396 | C3 |
| Glycogen Biosynthesis | 0.9945(0.8655-1.1427) | -0.0782 | 0.9377 | C3 |
| Lipoic Acid Metabolism | 0.9307(0.7245-1.1955) | -0.5622 | 0.574 | C3 |
| Lysine Degradation | 1.0901(0.8522-1.3943) | 0.6867 | 0.4923 | C3 |
| Oxidative Phosphorylation | 1.0303(0.8201-1.2944) | 0.2565 | 0.7975 | C3 |
| Pentose and Glucuronate Interconversions | 1.2686(1.0445-1.5409) | 2.399 | 0.0164 | C3 |
| Phenylalanine Tyrosine and Tryptophan Biosynthesis | 1.2641(1.0465-1.5268) | 2.4319 | 0.015 | C3 |
| Polyamine Biosynthesis | 1.2167(1.0294-1.4381) | 2.2999 | 0.0215 | C3 |
| Primary Bile Acid Biosynthesis | 1.0287(0.8565-1.2356) | 0.3028 | 0.7621 | C3 |
| Propanoate Metabolism | 1.221(1.0069-1.4806) | 2.0295 | 0.0424 | C3 |
| Prostaglandin Biosynthesis | 1.0318(0.8656-1.2299) | 0.3495 | 0.7267 | C3 |
| Pyruvate Metabolism | 1.1565(0.9699-1.379) | 1.6191 | 0.1054 | C3 |
| Remethylation | 1.1726(0.9878-1.3919) | 1.8195 | 0.0688 | C3 |
| Retinoid Metabolism | 0.793(0.6488-0.9693) | -2.2644 | 0.0236 | C3 |
| Riboflavin Metabolism | 0.9246(0.7667-1.1151) | -0.8201 | 0.4122 | C3 |
| Sirtuin Nicotinamide Metabolism | 0.9072(0.7527-1.0933) | -1.023 | 0.3063 | C3 |
| Ubiquinone and other Terpenoid Quinone Biosynthesis | 1.1081(0.9035-1.359) | 0.9855 | 0.3244 | C3 |
| Vitamin K | 1.1149(0.9207-1.35) | 1.1136 | 0.2655 | C3 |
| alpha Linoleic Acid Metabolism | 0.8587(0.754-0.978) | -2.3032 | 0.0213 | all |
| Lipoic Acid Metabolism | 0.8505(0.735-0.984) | -2.1745 | 0.0297 | all |
| Gluconeogenesis | 1.1321(0.999-1.282) | 1.9502 | 0.0512 | all |
| Caffeine Metabolism | 1.123(0.994-1.268) | 1.8691 | 0.0616 | all |
| Retinoid Metabolism | 0.9104(0.804-1.03) | -1.4855 | 0.1374 | all |
| Vitamin K | 1.1106(0.966-1.277) | 1.4731 | 0.1407 | all |
| Oxidative Phosphorylation | 0.9171(0.804-1.046) | -1.29 | 0.197 | all |
| Sirtuin Nicotinamide Metabolism | 0.9116(0.792-1.05) | -1.2872 | 0.198 | all |
| Prostaglandin Biosynthesis | 1.0752(0.943-1.226) | 1.0833 | 0.2787 | all |
| Propanoate Metabolism | 1.0698(0.937-1.222) | 0.9944 | 0.32 | all |
| Glycogen Biosynthesis | 1.0499(0.935-1.179) | 0.8246 | 0.4096 | all |
| Pyruvate Metabolism | 1.0478(0.924-1.188) | 0.7297 | 0.4655 | all |
| Pentose and Glucuronate Interconversions | 1.0424(0.917-1.185) | 0.6331 | 0.5267 | all |
| Lysine Degradation | 1.0461(0.909-1.204) | 0.6279 | 0.53 | all |
| Primary Bile Acid Biosynthesis | 1.0398(0.914-1.183) | 0.5914 | 0.5542 | all |
| Folate One Carbon Metabolism | 1.0327(0.905-1.179) | 0.476 | 0.634 | all |
| Phenylalanine Tyrosine and Tryptophan Biosynthesis | 0.9685(0.848-1.106) | -0.4718 | 0.6371 | all |
| Biotin Metabolism | 1.0295(0.901-1.176) | 0.4291 | 0.6679 | all |
| Remethylation | 1.0282(0.902-1.172) | 0.4174 | 0.6764 | all |
| Polyamine Biosynthesis | 0.9738(0.856-1.108) | -0.4021 | 0.6876 | all |
| Cardiolipin Biosynthesis | 0.9782(0.857-1.116) | -0.3276 | 0.7432 | all |
| Ubiquinone and other Terpenoid Quinone Biosynthesis | 0.983(0.865-1.117) | -0.2623 | 0.7931 | all |
| Fatty Acid Degradation | 1.0079(0.888-1.143) | 0.1221 | 0.9028 | all |
| Riboflavin Metabolism | 1.0039(0.877-1.149) | 0.0566 | 0.9549 | all |
