# Supplementary material for: Tumor Immunometabolism Characterization in Ovarian Cancer With Prognostic and Therapeutic Implications
Source: Front Oncol. 2021 Mar 16;11:622752. doi: 10.3389/fonc.2021.622752 (PMC8008085; doi:10.3389/fonc.2021.622752)
Supplement: Supplementary file 18 [file Table_9.doc]

**Supplementary Table S9: Association between characteristic metabolic pathways and MHC molecules**

| **Group: C1** | | | |
| --- | --- | --- | --- |
| **Pathway** | **Gene** | **Correlation** | ***P* value** |
| Amino Sugar and Nucleotide Sugar Metabolism | HLA-A | 0.36 | 2.98E-13 |
| Amino Sugar and Nucleotide Sugar Metabolism | HLA-C | 0.35 | 1.06E-12 |
| Amino Sugar and Nucleotide Sugar Metabolism | HLA-B | 0.37 | 4.77E-14 |
| Amino Sugar and Nucleotide Sugar Metabolism | TNF | 0.07 | 0.152469 |
| Amino Sugar and Nucleotide Sugar Metabolism | TAP2 | 0.09 | 0.05357 |
| Amino Sugar and Nucleotide Sugar Metabolism | TAP1 | 0.25 | 6.25E-07 |
| Amino Sugar and Nucleotide Sugar Metabolism | TAPBP | 0.06 | 0.221239 |
| Amino Sugar and Nucleotide Sugar Metabolism | IFNG | 0.18 | 0.000234 |
| Amino Sugar and Nucleotide Sugar Metabolism | B2M | 0.43 | 4.43E-19 |
| Arachidonic Acid Metabolism | HLA-A | 0.26 | 1.43E-07 |
| Arachidonic Acid Metabolism | HLA-C | 0.26 | 2.91E-07 |
| Arachidonic Acid Metabolism | HLA-B | 0.29 | 1.01E-08 |
| Arachidonic Acid Metabolism | TNF | 0.05 | 0.293594 |
| Arachidonic Acid Metabolism | TAP2 | 0.09 | 0.081238 |
| Arachidonic Acid Metabolism | TAP1 | 0.18 | 0.000287 |
| Arachidonic Acid Metabolism | TAPBP | 0.11 | 0.026163 |
| Arachidonic Acid Metabolism | IFNG | 0.24 | 1.64E-06 |
| Arachidonic Acid Metabolism | B2M | 0.38 | 2.24E-14 |
| Glycosaminoglycan Degradation | HLA-A | 0.27 | 9.22E-08 |
| Glycosaminoglycan Degradation | HLA-C | 0.3 | 1.35E-09 |
| Glycosaminoglycan Degradation | HLA-B | 0.32 | 7.01E-11 |
| Glycosaminoglycan Degradation | TNF | 0.04 | 0.414933 |
| Glycosaminoglycan Degradation | TAP2 | 0.19 | 0.000113 |
| Glycosaminoglycan Degradation | TAP1 | 0.27 | 9.44E-08 |
| Glycosaminoglycan Degradation | TAPBP | 0.32 | 2.34E-10 |
| Glycosaminoglycan Degradation | IFNG | 0.19 | 0.000184 |
| Glycosaminoglycan Degradation | B2M | 0.38 | 2.21E-14 |
| Glycosphingolipid Biosynthesis | HLA-C | 0.31 | 6.48E-10 |
| Glycosphingolipid Biosynthesis | HLA-B | 0.26 | 1.56E-07 |
| Glycosphingolipid Biosynthesis | TNF | 0.19 | 0.000155 |
| Glycosphingolipid Biosynthesis | TAP2 | 0.16 | 0.001868 |
| Glycosphingolipid Biosynthesis | TAP1 | 0.19 | 0.000152 |
| Glycosphingolipid Biosynthesis | TAPBP | 0.17 | 0.000794 |
| Glycosphingolipid Biosynthesis | IFNG | 0.03 | 0.521137 |
| Glycosphingolipid Biosynthesis | B2M | 0.37 | 3.09E-14 |
| Inositol Phosphate Metabolism | HLA-A | 0.02 | 0.563058 |
| Inositol Phosphate Metabolism | HLA-C | 0.08 | 0.110448 |
| Inositol Phosphate Metabolism | HLA-B | 0.11 | 0.026141 |
| Inositol Phosphate Metabolism | TNF | 0.13 | 0.011633 |
| Inositol Phosphate Metabolism | TAP2 | 0.26 | 1.84E-07 |
| Inositol Phosphate Metabolism | TAP1 | 0.17 | 0.000473 |
| Inositol Phosphate Metabolism | TAPBP | 0.34 | 5.02E-12 |
| Inositol Phosphate Metabolism | IFNG | 0.1 | 0.034578 |
| Inositol Phosphate Metabolism | B2M | 0.06 | 0.226222 |
| Nicotinate and Nicotinamide Metabolism | HLA-A | 0.27 | 6.24E-08 |
| Nicotinate and Nicotinamide Metabolism | HLA-C | 0.31 | 3.23E-10 |
| Nicotinate and Nicotinamide Metabolism | HLA-B | 0.33 | 3.12E-11 |
| Nicotinate and Nicotinamide Metabolism | TNF | 0.1 | 0.039436 |
| Nicotinate and Nicotinamide Metabolism | TAP2 | 0.21 | 3.47E-05 |
| Nicotinate and Nicotinamide Metabolism | TAP1 | 0.26 | 1.64E-07 |
| Nicotinate and Nicotinamide Metabolism | TAPBP | 0.13 | 0.006717 |
| Nicotinate and Nicotinamide Metabolism | IFNG | 0.14 | 0.003797 |
| Nicotinate and Nicotinamide Metabolism | B2M | 0.46 | 7.36E-22 |
| Other Glycan Degradation | HLA-A | 0.32 | 9.70E-11 |
| Other Glycan Degradation | HLA-C | 0.34 | 4.68E-12 |
| Other Glycan Degradation | HLA-B | 0.39 | 3.73E-15 |
| Other Glycan Degradation | TNF | 0.11 | 0.020691 |
| Other Glycan Degradation | TAP2 | 0.21 | 3.53E-05 |
| Other Glycan Degradation | TAP1 | 0.33 | 1.52E-11 |
| Other Glycan Degradation | TAPBP | 0.33 | 5.11E-11 |
| Other Glycan Degradation | IFNG | 0.17 | 0.000523 |
| Other Glycan Degradation | B2M | 0.41 | 3.30E-17 |

| **Group: C2** | | | |
| --- | --- | --- | --- |
| **Pathway** | **Gene** | **Correlation** | ***P* value** |
| ADP-Ribosylation | HLA-A | 0.46 | 1.38E-21 |
| ADP-Ribosylation | HLA-C | 0.45 | 4.46E-20 |
| ADP-Ribosylation | HLA-B | 0.51 | 2.07E-26 |
| ADP-Ribosylation | TNF | 0.15 | 0.003074 |
| ADP-Ribosylation | TAP2 | 0.61 | 5.72E-41 |
| ADP-Ribosylation | TAP1 | 0.57 | 1.25E-33 |
| ADP-Ribosylation | TAPBP | 0.52 | 2.88E-28 |
| ADP-Ribosylation | IFNG | 0.33 | 1.69E-11 |
| ADP-Ribosylation | B2M | 0.54 | 2.53E-30 |
| Amino Sugar and Nucleotide Sugar Metabolism | HLA-A | 0.36 | 2.98E-13 |
| Amino Sugar and Nucleotide Sugar Metabolism | HLA-C | 0.35 | 1.06E-12 |
| Amino Sugar and Nucleotide Sugar Metabolism | HLA-B | 0.37 | 4.77E-14 |
| Amino Sugar and Nucleotide Sugar Metabolism | TNF | 0.07 | 0.152469 |
| Amino Sugar and Nucleotide Sugar Metabolism | TAP2 | 0.09 | 0.05357 |
| Amino Sugar and Nucleotide Sugar Metabolism | TAP1 | 0.25 | 6.25E-07 |
| Amino Sugar and Nucleotide Sugar Metabolism | TAPBP | 0.06 | 0.221239 |
| Amino Sugar and Nucleotide Sugar Metabolism | IFNG | 0.18 | 2.34E-04 |
| Amino Sugar and Nucleotide Sugar Metabolism | B2M | 0.43 | 4.43E-19 |
| Drug Metabolism by other enzymes | HLA-A | 0.26 | 1.14E-07 |
| Drug Metabolism by other enzymes | HLA-C | 0.23 | 5.92E-06 |
| Drug Metabolism by other enzymes | HLA-B | 0.25 | 3.80E-07 |
| Drug Metabolism by other enzymes | TNF | 0.11 | 0.026241 |
| Drug Metabolism by other enzymes | TAP2 | 0.12 | 0.01894 |
| Drug Metabolism by other enzymes | TAP1 | 0.14 | 0.00411 |
| Drug Metabolism by other enzymes | TAPBP | 0.11 | 0.031211 |
| Drug Metabolism by other enzymes | IFNG | 0.15 | 0.002138 |
| Drug Metabolism by other enzymes | B2M | 0.3 | 1.94E-09 |
| Kynurenine Metabolism | HLA-C | 0.28 | 2.02E-08 |
| Kynurenine Metabolism | HLA-B | 0.34 | 3.80E-12 |
| Kynurenine Metabolism | TNF | 0.13 | 0.007281 |
| Kynurenine Metabolism | TAP2 | 0.17 | 6.35E-04 |
| Kynurenine Metabolism | TAP1 | 0.32 | 1.00E-10 |
| Kynurenine Metabolism | TAPBP | 0.08 | 0.109558 |
| Kynurenine Metabolism | IFNG | 0.29 | 1.04E-08 |
| Kynurenine Metabolism | B2M | 0.41 | 8.68E-17 |
| Lipoic Acid Metabolism | HLA-A | -0.08 | 0.162734 |
| Lipoic Acid Metabolism | HLA-C | -0.11 | 0.050684 |
| Lipoic Acid Metabolism | HLA-B | -0.12 | 0.022111 |
| Lipoic Acid Metabolism | TNF | -0.24 | 6.24E-06 |
| Lipoic Acid Metabolism | TAP2 | -0.18 | 6.33E-04 |
| Lipoic Acid Metabolism | TAP1 | -0.14 | 0.009558 |
| Lipoic Acid Metabolism | TAPBP | -0.32 | 6.82E-10 |
| Lipoic Acid Metabolism | IFNG | -0.16 | 0.003423 |
| Lipoic Acid Metabolism | B2M | -0.09 | 0.120242 |
| Nicotinate and Nicotinamide Metabolism | HLA-A | 0.27 | 6.24E-08 |
| Nicotinate and Nicotinamide Metabolism | HLA-C | 0.31 | 3.23E-10 |
| Nicotinate and Nicotinamide Metabolism | HLA-B | 0.33 | 3.12E-11 |
| Nicotinate and Nicotinamide Metabolism | TNF | 0.1 | 0.039436 |
| Nicotinate and Nicotinamide Metabolism | TAP2 | 0.21 | 3.47E-05 |
| Nicotinate and Nicotinamide Metabolism | TAP1 | 0.26 | 1.64E-07 |
| Nicotinate and Nicotinamide Metabolism | TAPBP | 0.13 | 0.006717 |
| Nicotinate and Nicotinamide Metabolism | IFNG | 0.14 | 0.003797 |
| Nicotinate and Nicotinamide Metabolism | B2M | 0.46 | 7.36E-22 |
| Oxidative Phosphorylation | HLA-A | -0.01 | 0.90935 |
| Oxidative Phosphorylation | HLA-C | -0.01 | 0.942494 |
| Oxidative Phosphorylation | HLA-B | -0.06 | 0.31743 |
| Oxidative Phosphorylation | TNF | -0.17 | 0.001006 |
| Oxidative Phosphorylation | TAP2 | -0.2 | 1.93E-04 |
| Oxidative Phosphorylation | TAP1 | -0.15 | 0.0043 |
| Oxidative Phosphorylation | TAPBP | -0.31 | 1.13E-09 |
| Oxidative Phosphorylation | IFNG | -0.09 | 0.09968 |
| Oxidative Phosphorylation | B2M | 0.06 | 0.18563 |
| Phenylalanine, Tyrosine and Tryptophan Biosynthesis | HLA-A | 0.28 | 1.57E-08 |
| Phenylalanine, Tyrosine and Tryptophan Biosynthesis | HLA-C | 0.26 | 1.78E-07 |
| Phenylalanine, Tyrosine and Tryptophan Biosynthesis | HLA-B | 0.31 | 4.13E-10 |
| Phenylalanine, Tyrosine and Tryptophan Biosynthesis | TNF | 0.27 | 8.35E-08 |
| Phenylalanine, Tyrosine and Tryptophan Biosynthesis | TAP2 | 0.29 | 4.75E-09 |
| Phenylalanine, Tyrosine and Tryptophan Biosynthesis | TAP1 | 0.31 | 3.42E-10 |
| Phenylalanine, Tyrosine and Tryptophan Biosynthesis | TAPBP | 0.31 | 2.56E-10 |
| Phenylalanine, Tyrosine and Tryptophan Biosynthesis | IFNG | 0.16 | 0.001356 |
| Phenylalanine, Tyrosine and Tryptophan Biosynthesis | B2M | 0.32 | 1.10E-10 |

| **Group: C3** | | | |
| --- | --- | --- | --- |
| **Pathway** | **Gene** | **Correlation** | ***P* value** |
| Epinephrine Biosynthesis | HLA-A | -0.15 | 0.003683 |
| Epinephrine Biosynthesis | HLA-C | -0.16 | 0.003361 |
| Epinephrine Biosynthesis | HLA-B | -0.23 | 1.18E-05 |
| Epinephrine Biosynthesis | TNF | -0.23 | 9.52E-06 |
| Epinephrine Biosynthesis | TAP2 | -0.28 | 6.68E-08 |
| Epinephrine Biosynthesis | TAP1 | -0.25 | 1.30E-06 |
| Epinephrine Biosynthesis | TAPBP | -0.32 | 5.27E-10 |
| Epinephrine Biosynthesis | IFNG | -0.2 | 1.05E-04 |
| Epinephrine Biosynthesis | B2M | -0.19 | 3.90E-04 |
| Lipoic Acid Metabolism | HLA-A | -0.08 | 0.162734 |
| Lipoic Acid Metabolism | HLA-C | -0.11 | 0.050684 |
| Lipoic Acid Metabolism | HLA-B | -0.12 | 0.022111 |
| Lipoic Acid Metabolism | TNF | -0.24 | 6.24E-06 |
| Lipoic Acid Metabolism | TAP2 | -0.18 | 6.33E-04 |
| Lipoic Acid Metabolism | TAP1 | -0.14 | 0.009558 |
| Lipoic Acid Metabolism | TAPBP | -0.32 | 6.82E-10 |
| Lipoic Acid Metabolism | IFNG | -0.16 | 0.003423 |
| Lipoic Acid Metabolism | B2M | -0.09 | 0.120242 |
