# Supplementary material for: Tumor Immunometabolism Characterization in Ovarian Cancer With Prognostic and Therapeutic Implications
Source: Front Oncol. 2021 Mar 16;11:622752. doi: 10.3389/fonc.2021.622752 (PMC8008085; doi:10.3389/fonc.2021.622752)
Supplement: Supplementary file 19 [file Table_10.doc]

**Table S10: Genes in the prognostic signature**

| **Gene** | **Description** | **Function** |
| --- | --- | --- |
| C5AR1 | Complement C5a Receptor 1 | Receptor activation stimulates chemotaxis, granule enzyme release, intracellular calcium release and superoxide anion production |
| CALM1 | Calmodulin 1 | Calmodulin mediates the control of a large number of enzymes, ion channels, aquaporins and other proteins through calcium-binding. Among the enzymes to be stimulated by the calmodulin-calcium complex are a number of protein kinases and phosphatases. |
| HLA-DOB | Major Histocompatibility Complex, Class II, DO Beta | Important modulator in the HLA class II restricted antigen presentation pathway by interaction with the HLA-DM molecule in B-cells. |
| PDP1 | Pyruvate Dehyrogenase Phosphatase Catalytic Subunit 1 | Catalyzes the dephosphorylation and concomitant reactivation of the alpha subunit of the E1 component of the pyruvate dehydrogenase complex. |
| PYGB | Glycogen Phosphorylase B | Glycogen phosphorylase that regulates glycogen mobilization is an important allosteric enzyme in carbohydrate metabolism |
| SIRT5 | Sirtuin 5 | SIRT5 target proteins are enriched in overlapping pathways including fatty acid β-oxidation, ketogenesis, and TCA cycle. |
| CERK | Ceramide Kinase | Catalyzes specifically the phosphorylation of ceramide to form ceramide 1-phosphate,CERK expression in human breast cancer is associated with an increased risk of recurrence within five years |
| LPCAT3 | Lysophosphatidylcholine Acyltransferase 3 | Acyltransferase which mediates the conversion of lysophosphatidylcholine into phosphatidylcholine |
| LPIN3 | Lipin 3 | Regulates fatty acid metabolism. Magnesium-dependent phosphatidate phosphatase enzyme which catalyzes the conversion of phosphatidic acid to diacylglycerol during triglyceride, phosphatidylcholine and phosphatidylethanolamine biosynthesis |
| PGM3 | Phosphoglucomutase 3 | Catalyzes the conversion of GlcNAc-6-P into GlcNAc-1-P during the synthesis of uridine diphosphate/UDP-GlcNAc, a sugar nucleotide critical to multiple glycosylation pathways including protein N- and O-glycosylation. |
| CLDN4 | Claudin 4 | Channel-forming tight junction protein that mediates paracellular chloride transport in the kidney. Plays a critical role in the paracellular reabsorption of filtered chloride in the kidney collecting ducts. |
| PC | Pyruvate Carboxylase | Pyruvate carboxylase catalyzes a 2-step reaction, involving the ATP-dependent carboxylation of the covalently attached biotin in the first step and the transfer of the carboxyl group to pyruvate in the second. |
| TRIM27 | Tripartite Motif Containing 27 | E3 ubiquitin-protein ligase that mediates ubiquitination of PIK3C2B and inhibits its activity; mediates the formation of 'Lys-48'-linked polyubiquitin chains; the function inhibits CD4 T-cell activation. |
| GALNT10 | Polypeptide N-Acetylgalactosaminyltransferase 10 | Catalyzes the initial reaction in O-linked oligosaccharide biosynthesis, the transfer of an N-acetyl-D-galactosamine residue to a serine or threonine residue on the protein receptor. Has activity toward Muc5Ac and EA2 peptide substrates. |
| GGCX | Gamma-Glutamyl Carboxylase | Mediates the vitamin K-dependent carboxylation of glutamate residues to calcium-binding gamma-carboxyglutamate (Gla) residues with the concomitant conversion of the reduced hydroquinone form of vitamin K to vitamin K epoxide. |
| PSMC1 | Proteasome 26S Subunit, ATPase 1 | Component of the 26S proteasome, a multiprotein complex involved in the ATP-dependent degradation of ubiquitinated proteins. This complex plays a key role in the maintenance of protein homeostasis by removing misfolded or damaged proteins, which could impair cellular functions, and by removing proteins whose functions are no longer required. |
| SIRT2 | Sirtuin 2 | NAD-dependent protein deacetylase, which deacetylates internal lysines on histone and alpha-tubulin as well as many other proteins such as key transcription factors |
| ECI2 | Enoyl-CoA Delta Isomerase 2 | Able to isomerize both 3-cis and 3-trans double bonds into the 2-trans form in a range of enoyl-CoA species. |
| GPAT4 | Glycerol-3-Phosphate Acyltransferase 4 | Converts glycerol-3-phosphate to 1-acyl-sn-glycerol-3-phosphate (lysophosphatidic acid or LPA) by incorporating an acyl moiety at the sn-1 position of the glycerol backbone |
| TPMT | Thiopurine S-Methyltransferase | Catalyzes the S-methylation of thiopurine drugs such as 6-mercaptopurine and 6-thioguanine using S-adenosyl-L-methionine as the methyl donor |
| DKK1 | Dickkopf WNT Signaling Pathway Inhibitor 1 | Antagonizes canonical Wnt signaling by inhibiting LRP5/6 interaction with Wnt and by forming a ternary complex with the transmembrane protein KREMEN that promotes internalization of LRP5/6 |
| GGT7 | Gamma-Glutamyltransferase 7 | Cleaves glutathione conjugates. |
| PCYT1A | Phosphate Cytidylyltransferase 1, Choline, Alpha | Controls phosphatidylcholine synthesis. |
| PPP3CA | Protein Phosphatase 3 Catalytic Subunit Alpha | Calcium-dependent, calmodulin-stimulated protein phosphatase which plays an essential role in the transduction of intracellular Ca(2+)-mediated signals |
| AKT1 | AKT Serine/Threonine Kinase 1 | AKT1 is one of 3 closely related serine/threonine-protein kinases (AKT1, AKT2 and AKT3) called the AKT kinase, and which regulate many processes including metabolism, proliferation, cell survival, growth and angiogenesis |
| MTMR14 | Myotubularin Related Protein 14 | Lipid phosphatase which efficiently dephosphorylates phosphatidylinositol 3-phosphate (PtdIns3P) and PtdIns(3,5)P2; inactive toward PtdIns4P, PtdIns(3,4)P2, PtdIns(4,5)P2 and PtdIns(3,4,5)P3. |
| TGFBR1 | Transforming Growth Factor Beta Receptor 1 | Transmembrane serine/threonine kinase forming with the TGF-beta type II serine/threonine kinase receptor, TGFBR2, the non-promiscuous receptor for the TGF-beta cytokines TGFB1, TGFB2 and TGFB3. |
